# Novel Inhibitory Actions of Neuroactive Steroid [3α,5α]-3-Hydroxypregnan-20-One on Toll-like Receptor 4-Dependent Neuroimmune Signaling

**DOI:** 10.3390/biom14111441

**Published:** 2024-11-13

**Authors:** Alejandro G. Lopez, Venkat R. Chirasani, Irina Balan, Todd K. O’Buckley, Makayla R. Adelman, A. Leslie Morrow

**Affiliations:** 1 Department of Biochemistry and Biophysics, University of North Carolina at Chapel Hill, Chapel Hill, NC 27599, USA; alopezsf@live.unc.edu (A.G.L.); venkatr@med.unc.edu (V.R.C.); 2 Bowles Center for Alcohol Studies, School of Medicine, University of North Carolina at Chapel Hill, 3027 Thurston Bowles Bldg., CB 7178, Chapel Hill, NC 27599, USA; 3 R. L. Juliano Structural Bioinformatics Core, School of Medicine, University of North Carolina at Chapel Hill, Chapel Hill, NC 27599, USA; 4 Department of Psychiatry, School of Medicine, University of North Carolina at Chapel Hill, Chapel Hill, NC 27599, USA; 5 Department of Pharmacology, School of Medicine, University of North Carolina at Chapel Hill, Chapel Hill, NC 27599, USA

**Keywords:** allopregnanolone, inflammation, toll-like receptor activation, TIRAP, lipid A

## Abstract

The endogenous neurosteroid (3α,5α)-3-hydroxypregnan-20-one (3α,5α-THP) modulates inflammatory and neuroinflammatory signaling through toll-like receptors (TLRs) in human and mouse macrophages, human blood cells and alcohol-preferring (P) rat brains. Although it is recognized that 3α,5α-THP inhibits TLR4 activation by blocking interactions with MD2 and MyD88, the comprehensive molecular mechanisms remain to be elucidated. This study explores additional TLR4 activation sites, including TIRAP binding to MyD88, which is pivotal for MyD88 myddosome formation, as well as LPS interactions with the TLR4:MD2 complex. Both male and female P rats (n = 8/group) received intraperitoneal administration of 3α,5α-THP (15 mg/kg; 30 min) or a vehicle control, and their hippocampi were analyzed using immunoprecipitation and immunoblotting techniques. 3α,5α-THP significantly reduces the levels of inflammatory mediators IL-1β and HMGB1, confirming its anti-inflammatory actions. We found that MyD88 binds to TLR4, IRAK4, IRAK1, and TIRAP. Notably, 3α,5α-THP significantly reduces MyD88-TIRAP binding (Males: −31 ± 9%, *t*-test, *p* < 0.005; Females: −53 ± 15%, *t*-test, *p* < 0.005), without altering MyD88 interactions with IRAK4 or IRAK1, or the baseline expression of these proteins. Additionally, molecular docking and molecular dynamic analysis revealed 3α,5α-THP binding sites on the TLR4:MD2 complex, targeting a hydrophobic pocket of MD2 usually occupied by Lipid A of LPS. Surface plasmon resonance (SPR) assays validated that 3α,5α-THP disrupts MD2 binding of Lipid A (Kd = 4.36 ± 5.7 μM) with an inhibition constant (Ki) of 4.5 ± 1.65 nM. These findings indicate that 3α,5α-THP inhibition of inflammatory mediator production involves blocking critical protein-lipid and protein-protein interactions at key sites of TLR4 activation, shedding light on its mechanisms of action and underscoring its therapeutic potential against TLR4-driven inflammation.

## 1. Introduction

Pro-inflammatory neuroimmune signaling is associated with numerous neurological, systemic, and psychiatric conditions, many of which lack widely accepted treatments. These conditions include alcohol and substance abuse disorders, depression, traumatic brain injury, and refractory epilepsies [1,2,3,4,5,6,7,8]. Generally, innate immune signaling is the first line of defense against infectious and toxic molecules that activate Toll-like receptor (TLR) pathways to initiate the immune response. Once activated, the signaling cascade upregulates the production of chemokines and cytokines which aid in mounting an immediate immune response to pathogens. However, these pathways can also be activated by cellular and environmental stressors, or may persist following infection, leading to systemic inflammation and neuroinflammation that have deleterious effects on all organs, including the brain. TLR proteins are transmembrane receptors that belong to the interleukin-1 receptor/Toll-like receptor superfamily and function as germline-encoded pattern-recognition receptors that initiate the inflammatory cascade leading to inflammatory diseases.

TLR4 activation is linked to many inflammatory states, and its activation mechanisms are well studied. TLR4 is associated with a wide array of systemic and central nervous system (CNS) disease-like states, such as sepsis, rheumatoid arthritis, chronic obstructive pulmonary disease, depression, schizophrenia, and bipolar disorder [1,9,10,11]. TLR4 exists in a monomeric state on the cell surface, bound to TLR4 co-receptor, myeloid differentiation factor 2 (MD-2). TLR4 activation is initiated at its extracellular domain, through the binding of lipopolysaccharide (LPS) to the TLR4:MD-2 complex. The next step is TLR4 dimerization which begins in the cytosol and is dependent upon the Toll/interleukin-1 (IL-1) receptor (TIR) domain of TLR4 [12,13]. The TIR domain containing an adaptor protein (TIRAP, previously known as MAL) is then responsible for the recruitment of myeloid differentiation primary response 88 (MyD88) to the TLR4 TIR domain to initiate myddosome complex formation [14,15,16]. TIRAP first binds membrane-bound Phosphatidylinositol 4,5-bisphosphate (PIP2) through its PIP2 binding motif near TLR4-rich membrane regions [17,18], then it attaches MyD88 to the TLR4 dimer, which serves as a scaffold that promotes the recruitment of additional key myddosome proteins, such as interleukin-1 receptor associated kinases (IRAKs) [19]. The formation of the complete heteromeric complex leads to pathway activation and the production of cytokines and chemokines, such as HMGB1 and IL-1β, propagating the inflammatory response.

In recent years, neurosteroids have gathered attention for their ability to modulate systemic and neuroimmune signaling [20,21]. Recent work has shown that the endogenous neurosteroid, (3α,5α)3-hydroxypregnan-20-one (allopregnanolone, 3α,5α-THP), decreases pro-inflammatory signaling through the inhibition of TLR4-dependent inflammatory responses in human and mouse macrophages, human blood cells, and alcohol preferring (P) rat brains [22,23,24,25]. In this work, coimmunoprecipitation studies revealed that MyD88 binding to TLR proteins is inhibited in the presence of 3α,5α-THP in P rat brains, providing some of the first insight into the mechanism behind 3α,5α-THP inhibitory actions [26]. Additionally, work in mouse macrophage RAW264.7 cells has shown that 3α,5α-THP affects TLR4 and MD-2 interactions, decreasing their binding in immunoprecipitation assays. However, since MD-2 is responsible for the binding of LPS to the TLR4 complex and MyD88 recruitment and myddosome formation depend upon the presence of TIRAP, the effects of 3α,5α-THP on LPS:MD-2 interactions and TIRAP:MyD88 binding need to be addressed.

Here, we investigate these potential sites of 3α,5α-THP action within the TLR4 signaling cascade. These studies were divided into two general areas of interest for TLR4 signaling: the events that occur extracellularly and intracellularly in the cytosol. As TLR4 signaling begins at the extracellular component through the agonist-induced LPS binding, we sought to probe the possibility of 3α,5α-THP binding to the TLR4:MD-2 complex. As previous drug discovery efforts have shown small molecule-induced disruption of LPS binding to the TLR4:MD-2 complex inhibits pro-inflammatory signaling [27,28], it is conceivable that 3α,5α-THP may hold similar properties. Consequentially, LPS binding triggers cytosolic TIRAP localization, MyD88 recruitment, and myddosome formation with IRAK 4/1/2 proteins. The inhibition of TIRAP binding to MyD88 by 3α,5α-THP was further explored as a mechanism that could inhibit myddosome formation and TLR pathway activation.

## 2. Materials and Methods

### 2.1. Animals

Alcohol-preferring (P) rats were chosen due to elevated innate immune signaling proteins such as TLR4 and the nuclear factor kappa-light-chain-enhancer of activated B cells (NF-κB) [29,30]. P rats (males n = 32; females n = 32) were obtained from the Alcohol Research Center, Indiana University School of Medicine. Rats were housed in Tecniplast cages, kept on a 12 h light-dark cycle, and fed ad libitum. All animal procedures were approved by the UNC Institutional Animal Care and Use Committee. P rats (3α,5α-THP males: n = 16; 3α,5α-THP females: n = 16; vehicle males: n = 16; vehicle females: n = 16) were randomly administered 3α,5α-THP (15 mg/kg) or vehicle (45% *w*/*v* 2-hydroxypropyl-β-cyclodextrin) in the morning to avoid circadian-induced neurosteroid fluctuations. Brain tissue was collected following 30 min post 3α,5α-THP treatment and brains were snap frozen and stored (−80 °C) until the extraction process. The hippocampal region was extracted on ice using a brain matrix and neuroanatomical landmarks established as referenced [31]. The hippocampus plays a central role in memory formation and emotional regulation processes critical to the development of alcohol use disorders (AUDs). Given its significance, our study targeted this brain region to delve into the molecular mechanisms modulating neuroinflammatory signaling. Additionally, we selected the hippocampus for its large size and abundance of available protein, making it ideal for co-immunoprecipitation studies.

### 2.2. Antibodies

Antibodies used in immunoblotting were commercially purchased, validated, and used following each manufacturer’s protocol. Proteins of interest were detected using primary antibodies which are listed in the provided Supplementary Materials (Supplementary Table S1).

### 2.3. Immunoblotting

Hippocampal tissues from vehicle- and 3α,5α-THP-treated animals were lysed using CelLytic MT (Sigma Aldrich, St. Louis, MO, USA, Cat. #C3228) supplemented with protease and phosphatase inhibitors (1:100 concentration; Protease Inhibitor Cocktail, Sigma Aldrich Cat #P8340; Phosphatase Inhibitor Cocktail, Sigma Aldrich Cat #P2850). Tissue lysate was sonicated twice for 30 s and centrifuged (14,000 rpm; 30 min). Total protein was determined by bicinchoninic assay (BCA, ThermoFisher Scientific, Waltham, MA, USA, Cat. #23228 and #1859078). To avoid freeze and thaw cycles, the extracted protein was distributed into one-time use aliquots of 500 µg total protein and stored (−80 °C). The proteins (40 μg/lane) were separated by SDS–polyacrylamide gel electrophoresis (SDS-PAGE), transferred to PVDF membrane, blocked (5% blotting grade milk or 5% Bovine Albumin Serum (BSA)), and exposed to primary antibody overnight with gentle agitation (4 °C), followed by horseradish peroxidase-labeled secondary antibodies. Immunoreactive bands were visualized with the Plus-ECL kit reagents and were imaged using ImageQuant 800 (Version 2.0.0) and analyzed using ImageQuant TL software (Version 8.1.0.0). Densitometric measurements were normalized by the corresponding β-actin densitometric measurement, where target proteins were normalized by corresponding lane’s normalization factor, and the results were expressed as the mean β-Actin-adjusted densitometric units ± SEM.

### 2.4. Immunoprecipitation

Co-immunoprecipitation of the myddosome was adapted from myddosome isolation in murine macrophages and modified to isolate myddosome proteins from the P rat brain [32]. CelLytic extracted protein lysates (500 µg) were incubated with goat anti-MyD88 antibody (6 µg: R&D Systems Cat. #AF3109) and incubated for 4 h (4 °C). Similarly, protein lysate was incubated with 6 µg of TLR4 antibody (Santa Cruz Biotechnology, Dallas, TX, USA, SC-25). Complexes were immunoprecipitated using Protein G Agarose resin (40 µL; Santa Cruz Biotechnology, #SC-2002) and left overnight on a rocker at 4 °C. To collect immunoprecipitated myddosome, samples were centrifuged at 3000× *g* for 30 s at 4 °C. The supernatant was delicately aspirated using a vacuumed Pasteur pipette, leaving roughly 10 µL volume reserve to not disrupt pelleted resin. The pellet was then washed through resuspension in 500 µL Pierce IP Lysis Buffer (Thermo Fisher Scientific, #87787), spun at 300× *g* for 30 s, vacuum aspirated, and repeated for a total of three washes. Once the final wash was completed, a fine-end gel loading pipette tip was carefully inserted into the resin bed and the remaining wash buffer was discarded. The resin was immediately resuspended in 4× Laemmli denaturing buffer (Bio-Rad, Hercules, CA, USA, Cat. #1610747) with 10% β-mercaptoethanol, boiled on a heat block for 5 min at 95 °C and resolved by SDS–polyacrylamide gel (SDS-PAGE) electrophoresis (NuPAGE™ 4–12%, Bis-Tris Gels Thermo Fisher Scientific, Waltham, MA, USA, #NP0322PK2).

### 2.5. Molecular Docking

TLR4:MD-2 structure (3FXI) was obtained from the Research Collaboratory for Structural Bioinformatics RCSB Protein Data Bank (RCSB PDB). Protein structure was visualized and prepared using BIOVIA Discovery Studios Visualizer software (Version 21.1.0.20298, Dassault Systems, San Diego, CA, USA). The ligand structure of 3α,5α-THP was obtained from the Zinc20 Chemical Database and converted to the PDBQT format compatible for docking using Open Babel GUI [33]. The TLR4:MD-2 complex was prepared by assigning appropriate polar hydrogen charges, Autodock vina compatible atom types, and Gasteiger charges in MGL Tools (Version 1.5.7). Additionally, torsible bonds of 3α,5α-THP were allowed to be free as assigned using MGL Tools. AutoDock Vina (Version 1.2.0) was then used for the docking TLR4:MD-2 and the calculation of docking scores [34]. The grid box dimensions for the docking study were set to 54 × 40 × 58 Å^3^, with the center coordinates at x = −4.658, y = 2.529, and z = −2.961. This grid box was designed to encompass the entire binding site of MD-2, ensuring comprehensive coverage of potential binding poses. The docked ligand and protein were imported into Discovery Studios Visualizer for analysis of ligand interactions. A total of 20 docking poses were generated, which were subsequently ranked based on binding energy, allowing for the selection of poses with a maximum energy difference of 3 kcal/mol and a minimum root-mean-square deviation (RMSD) threshold of 1.0 Å.

### 2.6. Surface Plasmon Resonance

SPR data were collected using OpenSPR-XT instrument (Nicoya, CR, Canada). Recombinant human MD-2 (R&D Systems Inc., Minneapolis, MN, USA, #1787-MD-050/CF) was covalently immobilized on High Sensitivity Carboxyl Sensors (Nicoya, Kitchener, ON, Canada) and 1:1 N-hydroxysuccinimide (NHS)/1-ethyl-3-(3-dimethylaminopropyl) carbodiimide hydrochloride (EDC) reactive coupling reactivity. Preconcentrated MD-2 (10 mM Acetate Buffer, pH 5.5) was injected over an activated carboxyl sensor at 30 µg/mL with a flow rate of 20 µL/min (1× PBS, 0.1% Tween-20), resulting in capturing roughly 4000 RU. The remaining active carboxyl sites were blocked using 1 M Ethanolamine (pH 8.5, Nicoya) for 300 s. Binding assays were performed using 1× PBS (0.05% Tween-20). Lipid A (AdipoGen Life Sciences, San Diego, CA, USA, IAX-100-004-M001) was prepared using the manufacturer’s protocol. Briefly, a stock solution of 1 mg/mL was heated to 40 degrees Celsius for 5 min before diluting to a working concentration in running buffer to mitigate Lipid A aggregation. Lipid A was serially diluted into 5 concentrations of two-fold dilutions (36–4.5 µM) in analyte running buffer (1× PBS + 0.05% Tween-20). All reported response curves were double referenced and subtracted from the reference flow cell and buffer blanks. Binding data were analyzed using TraceDrawer SPR evaluation software (Version 1.9.2, Uppsala, Sweden) and fitted with a 1:1 binding model. For Lipid A and 3α,5α-THP competition assays, hMD-2 was immobilized, and Lipid A prepared as previously described. The concentration of Lipid A was chosen based on the ability to provide an adequate response (RU) and 3α,5α-THP concentrations were selected after screening a range of concentrations that fit within physiologically relevant doses. 3α,5α-THP was serially diluted using two-fold dilutions and added to Lipid A just before beginning the assay.

### 2.7. Molecular Dynamics Simulations

Molecular dynamics simulations were performed to study the interactions between the MD-2 and 3α,5α-THP using GROMACS 2021.5 [35] with the CHARMM36 force field [36]. To prepare the simulation system, the MD-2 structure was solvated in a box filled with TIP3P water molecules, and NaCl was added to maintain a physiological ionic strength of 150 mM. The system underwent energy minimization using the steepest descent algorithm to eliminate unfavorable steric clashes. Following this, a two-step equilibration process was performed: first in the NVT ensemble for 5 ns, followed by NPT equilibration for 10 ns. The production simulations lasted 1000 ns, employing a constant temperature of 303.15 K and pressure of 1 bar, controlled by the v-rescale thermostat and C-rescale barostat [37], respectively. The LINCS algorithm was applied to constrain all hydrogen bonds [38], and long-range electrostatics were handled using the Particle Mesh Ewald (PME) method [39]. A cutoff of 1.2 nm was set for both van der Waals and electrostatic interactions.

### 2.8. Trajectory Analysis

Post-simulation analysis was conducted using built-in GROMACS tools and custom scripts. Root-mean-square deviation (RMSD) and root-mean-square fluctuation (RMSF) analyses were performed to assess the stability and flexibility of MD-2 over the simulation period. Additionally, non-bonded contacts between 3α,5α-THP and MD-2 were thoroughly analyzed. The protein–ligand interactions were visualized and analyzed using LigPlot+ [40] and trajectory movies were created using VMD [41] to illustrate the dynamic behavior of the MD-2 + 3α,5α-THP complex.

### 2.9. Statistics

Densitometric measurements of immunoblots using lysate of P rat brain tissue from groups treated with 3α,5α-THP or vehicle were analyzed using Shapiro–Wilk normality testing and then analyzed using a two-way ANOVA. Analysis of co-immunoprecipitation immunoblots, compared with 3α,5α-THP- and vehicle-treated groups, were analyzed using unpaired two-tail *t*-tests. All statistical analysis was performed using Graphpad Prism (Version 10.3.1. Graphpad Software, Boston, MA, USA).

## 3. Results

### 3.1. Effects of 3α,5α-THP on HMGB1 and IL-1β in P Rat Brain

Pro-inflammatory mediators such as HMGB1 and IL-1β play a crucial role in systemic and central nervous system immune regulation, specifically TLR4 signaling. TLR4 signaling was innately activated in P rat brains in comparison with the control, serving as an ideal animal model to study the effects of 3α,5α-THP on TLR4 signaling in the brain. TLR4 activation propagated pro-inflammatory immune responses through the adaptor protein, MyD88, then NF-κB was phosphorylated (activated) and upregulated pro-inflammatory cytokines such as IL-1β. The increase in cytokines prompted the release of HMGB1 from neurons, allowing further propagation of TLR4 and other TLRs [42,43].

**Figure 1:**
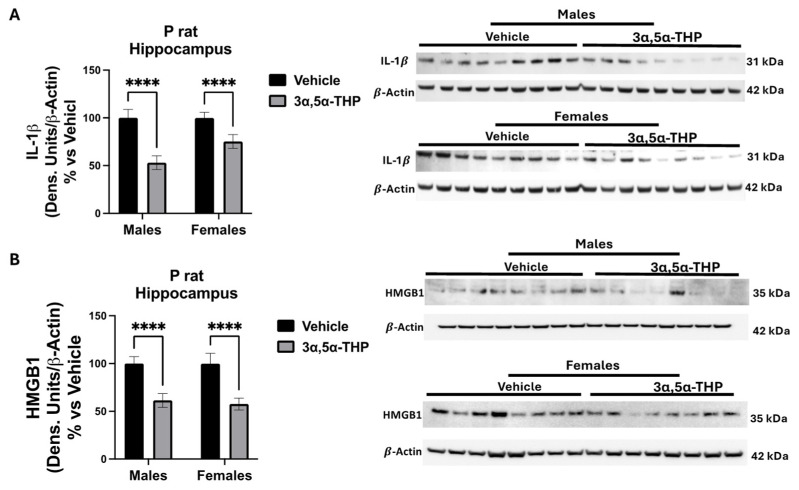
3α,5α-THP reduces HMGB1 and IL-1β protein levels in P rat hippocampus. Male and female alcohol-preferring (P) rats (n = 8/group) were treated with 3α,5α-THP (15 mg/kg; 30 min) or the vehicle (45% *w*/*v* 2-hydroxypropyl-β-cyclodextrin; 30 min) and hippocampus lysate was examined for IL-1β and HMGB1 protein expression. Data were collected from two separate P rat cohorts and protein expression was analyzed via immunoblotting with data expressed as the percent change from the control (vehicle) in each sex. (**A**) IL-1β expression was decreased in both male P rats by 46.95% ± 11.37 (2-way ANOVA: F(1,69) = 23, *p* < 0.0001 ****, n = 18/vehicle and n = 18/3α,5α-THP) and female P rats by 24.68% ± 9.23 (2-way ANOVA: F(1,69) = 23, *p* < 0.0001 ****, n = 18/vehicle and n = 18/3α,5α-THP). (**B**) The expression of hippocampal HMGB1 was also tested in male and female P rats. HMGB1 was decreased in males by 38.60% ± 10.12 (2-way ANOVA: F(1,66) = 25.85, *p* < 0.0001 ****, n = 18/vehicle and n = 18/3α,5α-THP) and 42.36% ± 12.58 (2-way ANOVA: F(1,66) = 25.85, *p* < 0.0001 ****, n = 18/vehicle and n = 17/3α,5α-THP) in females. Western blot original images are in the Supplementary Materials.

To test the effects of 3α,5α-THP on HMGB1 and IL-1β production in the P rat hippocampus, both male and female P rats were administered 3α,5α-THP (15 mg/kg) or a vehicle control (45% *w*/*v* 2-hydroxypropyl-β-cyclodextrin) for 30 min (n = 9/group). We found that IL-1β expression was reduced in male P rat hippocampus by 46.95% ± 11.37 (two-way ANOVA: F(1,69) = 23, *p* < 0.0001, n = 18/vehicle and n = 18/3α,5α-THP) and female P rats by 24.68% ± 9.232 (two-way ANOVA: F(1,69) = 23, *p* < 0.0001, n = 18/vehicle and n = 18/3α,5α-THP). Additionally, the expression of HMGB1 was reduced in P rat males by 38.60% ± 10.12 (two-way ANOVA: F(1,66) = 25.85, *p* < 0.0001, n = 18/vehicle and n = 18/3α,5α-THP) and in females by 42.36% ± 12.58 (two-way ANOVA: F(1,66) = 25.85, *p* < 0.0001, n = 18/vehicle and n = 17/3α,5α-THP), respectively (Figure 1A,B). Throughout this study, male and female P rats were analyzed separately and the effects of 3α,5α-THP compared by two-way ANOVA because sex differences were previously reported in human monocyte-derived macrophages treated with different neurosteroid compounds [22]. However, no effect of sex was found for the IL-1β or HMGB1 expressions or the effects of 3α,5α-THP on HMGB1 and IL-1β levels in this study.

### 3.2. Effects of 3α,5α-THP on TIRAP:MyD88 Binding in P Rat Hippocampus

**Figure 2:**
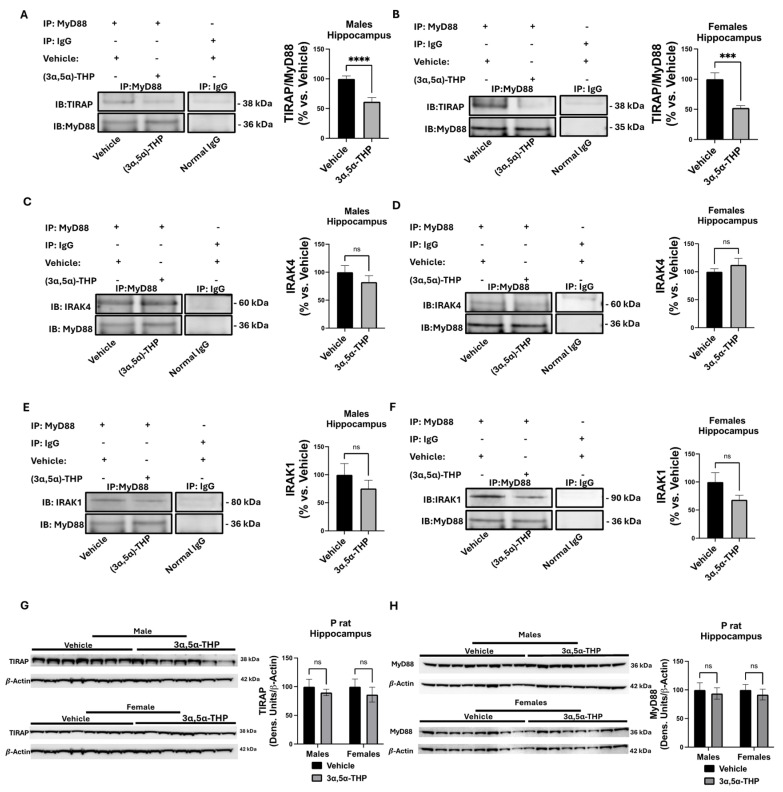
3α,5α-THP inhibits TIRAP binding to MyD88 in P rat male (**A**) and female (**B**) hippocampi. MyD88 immunoprecipitation of various components of the myddosome complex was conducted in male and female, vehicle- (45% *w*/*v* 2-hydroxypropyl-β-cyclodextrin; 30 min) and 3α,5α-THP-treated (15 mg/kg; 30 min) P rats (**A**,**B**). Densiometric comparison of the effect of 3α,5α-THP on MyD88 immunoprecipitation of TIRAP (**A**,**B**), IRAK4 (**C**,**D**) and IRAK1 (**E**,**F**) in female and male hippocampi. Immunoblots and densiometric measurements of extracted hippocampal TIRAP (**G**) and MyD88 (**H**) in vehicle- and 3α,5α-THP-treated animals. Western blot original images are in the Supplementary Materials. *** *p* < 0.005, **** *p* < 0.001, ns: no significant difference.

Previous work has shown that 3α,5α-THP disrupts binding between MyD88 and TLR4 in RAW264.7 cells and P rat amygdala and nucleus accumbens [26]. However, studies on macrophages and mouse embryonic fibroblasts have shown that MyD88 function in TLR4 signaling is induced upon TIRAP localization to the cell membrane, in proximity to activated TLR4 [18]. Coimmunoprecipitation studies were conducted to assess MyD88 interactions with TIRAP, IRAK4, and IRAK1 in male and female P rat hippocampal tissues following vehicle or 3α,5α-THP administration. MyD88 antibody was used for immunoprecipitation to isolate the complex, and antibodies against TIRAP, IRAK4, and IRAK1 were used for immunoblotting to detect these proteins. MyD88 immunoprecipitated TIRAP, and the administration of 3α,5α-THP decreased TIRAP binding to MyD88 by 38.49% ± 8.28% in males (*t*-test, *p* < 0.0001, n = 10/group) and by 47.65% ± 11.13% in females (*t*-test, *p* = 0.0005, n = 10/group) (Figure 2A,B).

The two additional key myddosome proteins, IRAK4 and IRAK1, were recruited to the TLR4:TIRAP:MyD88 complex during TLR4 activation, and once recruited formed the initial component of the myddosome. We found that both IRAK4 and IRAK1 immunoprecipitated with MyD88, however the binding of the IRAK proteins was not altered in the hippocampus of male and female P rats following 3α,5α-THP administration (Figure 2C,D). Additionally, 3α,5α-THP did not alter MyD88 (two-way ANOVA: F(1,27) = 0.49, *p* = 0.49, n = 8/group) or TIRAP (two-way ANOVA: F(1,27) = 1.1, *p* = 0.3025, n = 8/group) levels in both the male and female P rat hippocampi (Figure 2G,H).

### 3.3. 3α,5α-THP Does Not Alter the Expression of TLR4 and Key MyD88-Associated Proteins

Since 3α,5α-THP inhibited the binding of MyD88 to TIRAP, it was essential to determine if it altered the expression of TLR4 and the other MyD88-associated proteins. Bruton’s Tyrosine Kinase (BTK) and endosome-microtubule linker protein 170 (CLIP-170) have been observed to play pivotal roles in the function of TIRAP. BTK is responsible for the tyrosine phosphorylation of TIRAP during TLR4 activation, which is required for binding to MyD88 and TLR4, and thus the propagation of immune signaling. CLIP170 has been observed to negatively regulate TLR4 signaling through CLIP170-mediated ubiquitination and consequentially the degradation of TIRAP. Additionally, previous studies have shown that the precursor of 3α,5α-THP, pregnenolone, induces the ubiquitination and degradation of TIRAP, thus suppressing TLR4 signaling in microglial cells [44]. Expression levels were measured via immunoblotting hippocampal lysate from vehicle- and (3α,5α)-THP-treated male and female P rats and are described in Table 1. No effects of 3α,5α-THP on basal levels of these proteins were observed (Table 1).

### 3.4. 3α,5α-THP Has No Effect on TLR4:MD2 Binding in P Rat Hippocampus

**Figure 3:**
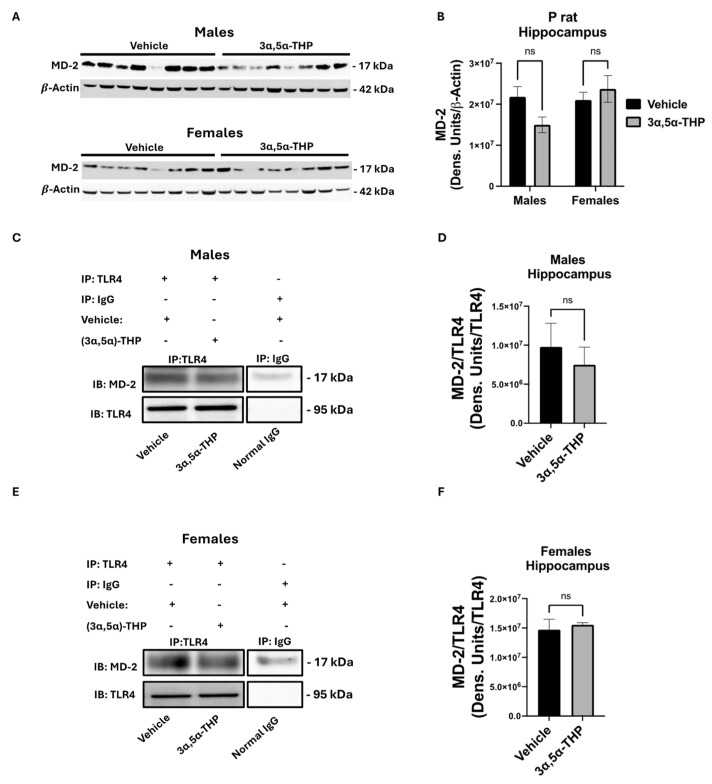
3α,5α-THP has no effect on TLR4 binding to MD-2 in the hippocampus of male and female P rats. (**A**,**B**) Hippocampus whole lysate was immunoblotted for the presence of MD-2 in vehicle- (45% *w*/*v* 2-hydroxypropyl-β-cyclodextrin; 30 min) and 3α,5α-THP-treated (15 mg/kg; 30 min) P rats and densiometric comparison of the effect of 3α,5α-THP on MD-2 expression. (**C**,**E**) Immunoprecipitation of TLR4/MD-2 and densiometric comparison of MD2 between vehicle and 3α,5α-THP-treated P rats (male: *t*-test, *p* = 0.56, n = 4/group female: (*t*-test, *p* = 0.46, n = 4/group) (**D**,**F**). Western blot original images are in the Supplementary Materials. ns: no significant difference.

Previous studies showed that 3α,5α-THP blocks TLR4 activation in mouse macrophage RAW264.7 cells by inhibiting the binding of MD2 to TLR4 in the presence of LPS [23]. To determine if this mechanism is applicable in the P rat brain, we first checked whether MD-2 was present in the hippocampus of the P rats. Our results show that MD-2 is expressed in both the male and female P rat hippocampus and 3α,5α-THP had no effect on its levels (two-way ANOVA: F(1,29) = 0.65, *p* = 0.43, n = 8/group) (Figure 3A,B). We then used the TLR4 antibody to immunoprecipitate the TLR4:MD-2 complex in the hippocampus and measured the binding of MD2. 3α,5α-THP had no effect on TLR4 immunoprecipitation of MD-2 in the male (*t*-test, *p* = 0.56, n = 4/group) or female (*t*-test, *p* = 0.46, n = 4/group) P rats (Figure 3C–F).

### 3.5. 3α,5α-THP Docks in MD-2 Pocket and Prevents Lipid A Binding

**Figure 4:**
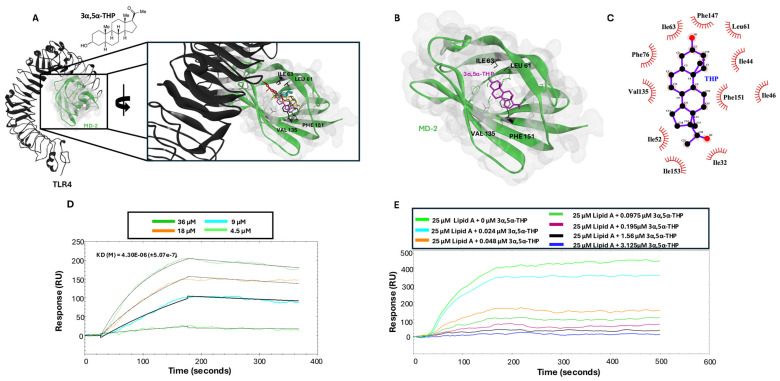
3α,5α-THP docks TLR4-bound MD-2 and inhibits Lipid A binding to MD-2 in SPR studies. (**A**) Molecular docking shows that 3α,5α-THP favors binding to MD-2, with multiple docking poses of 3α,5α-THP found within the MD-2 binding pocket. (**B**) The top pose of 3α,5α-THP in MD-2 shows multiple π-alkyl bonds formed with key MD-2 residues. (**C**) Two-dimensional representation of 3α,5α-THP binding MD-2, depicting the amino acid interactions. (**D**) SPR studies show Lipid A binds immobilized MD-2 with a K_D_ = 4.3 ± 0.5 µM (k_a_ = 1.5 × 10^2^ M^−1^S^−1^, k_d_ = 6.78 × 10^−4^ S^−1^). (**E**) SPR competition assay showing that 3α,5α-THP competitively binds MD2, as increasing concentrations of 3α,5α-THP decrease Lipid A binding (**D**).

To test potential binding sights of the 3α,5α-THP inhibition of TLR4 signaling, we used molecular docking to screen for possible inhibitory sites on the TLR4:MD-2 complex. Initially, we used a blind docking approach of the entire TLR4:MD-2 complex. Top docking poses of 3α,5α-THP showed favorable docking into the hydrophobic pocket of MD-2 (Figure 4). We then re-docked 3α,5α-THP, focusing the grid coordinates on the area of the TLR4:MD-2:LPS dimerization interface. Predicated poses showed affinities over a range of −8.6–7.6 kcal/mol, with the top poses having values of −8.6–7.8 kcal/mol. Of particular interest, MD-2 residues VAL 135, LEU61, ILE 63, and PHE151 were shown to interact with the C and D rings of the 3α,5α-THP steroidal backbone.

Since the TLR4:MD-2 complex is required for activation by the well-studied TLR4 agonist, lipopolysaccharide (LPS), we considered the possibility that (3α,5α)-THP may prevent binding of LPS to the TLR4:MD-2 complex. Specifically, point mutations at VAL135 have been shown to reduce MD-2 binding of the CD14-transported LPS [45].The large LPS molecule can be segmented into three regions: O-antigen, core sugars (both outer and inner core), and Lipid A. The hydrophobic pocket of MD-2 accommodates the hydrophobic fatty acyl chains of Lipid A [46], but direct binding of Lipid A to MD2 has not previously been demonstrated. To address this gap in our knowledge and to determine if (3α,5α)-THP inhibits the binding of Lipid A to MD-2, we conducted Surface Plasmon Resonance (SPR) assays. SPR analysis shows that Lipid A binds MD-2, providing a binding affinity of KD = 4.30 ± 0.5 μM. This binding affinity is within reason, as previous SPR data have described LPS binding MD-2 with an affinity of 2.3 µM [47]. We then conducted SPR competition assays to examine the potential inhibition of Lipid A binding to MD-2 in the presence of 3α,5α-THP. We found that 3α,5α-THP (3.125 µM–240 nM) inhibited the binding of Lipid A to MD-2 in a dose-dependent manner, providing an inhibitory constant (Ki) of 4.5 ± 1.7 nM.

### 3.6. MD-2 Maintains Stability During Simulations

**Figure 5:**
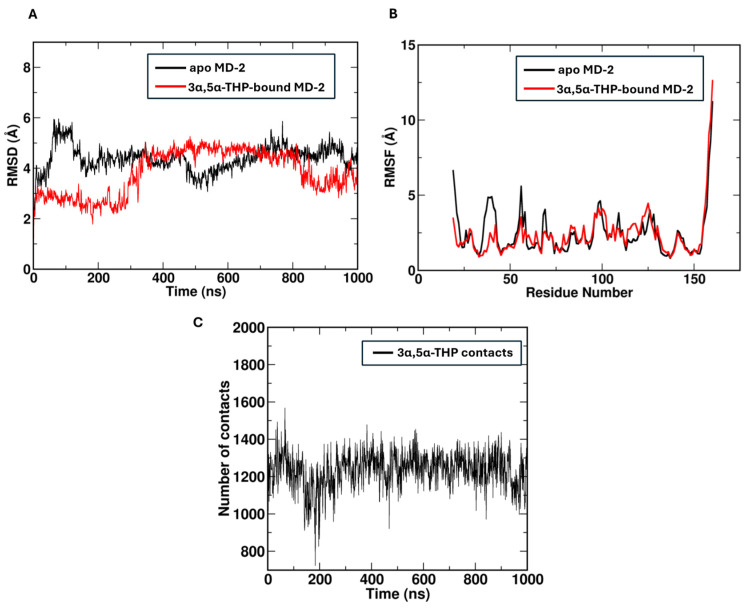
Structural and interaction analysis of MD-2 with 3α,5α-THP binding. Panels a–c present the key findings from MD simulations and trajectory analysis of the MD-2 complexed with the neuroactive steroid (3α,5α)-3-hydroxypregnan-20-one (3α,5α-THP), performed using GROMACS with the CHARMM36 force field. (**A**) The root-mean-square deviation (RMSD) of the backbone atoms of MD-2 relative to its initial structure is plotted over the 1000 ns simulation trajectory for both the 3α,5α-THP-bound (red) and apo (black) states. (**B**) The root-mean-square fluctuation (RMSF) of individual Cα atoms of MD-2 is plotted over the 1000 ns simulation trajectory for both the 3α,5α-THP-bound (red) and apo (black) states. (**C**) The average number of non-bonded contacts between 3α,5α-THP and MD-2, calculated across the simulation. To calculate the non-bonded contacts between 3α,5α-THP and MD-2, the gmx mindist tool was used. A cutoff distance of 5 Å was applied, which defines contacts as any non-bonded interactions where the distance between atoms of 3α,5α-THP and MD-2 is 5 Å or less. On average, approximately 1200 contacts were observed between 3α,5α-THP and MD-2, indicative of stable interactions within the binding pocket.

The root-mean-square deviation (RMSD) analysis of MD-2 indicated stable conformational behavior throughout the simulations, both in the presence and absence of 3α,5α-THP (Figure 5A). In the presence of 3α,5α-THP, MD-2 demonstrated increased structural stability, particularly evident when comparing the initial and final 200 ns segments of the simulation. During these periods, the MD-2 + 3α,5α-THP complex maintained a lower RMSD than apo-MD-2, suggesting that 3α,5α-THP binding reinforced the structural integrity of MD-2 over time. This added stability may have enhanced MD-2’s capacity to bind with a higher affinity to its interaction partners, as 3α,5α-THP appeared to help maintain the structural conformation necessary for effective binding.

### 3.7. 3α,5α-THP Binding Enhances Residue Stability

In the root-mean-square fluctuation (RMSF) analysis, notable increases in fluctuation were observed in the residues spanning positions 19 to 70 when 3α,5α-THP was absent (Figure 5B). This trend indicates that the binding of 3α,5α-THP may play a critical role in enhancing the stability of specific regions within MD-2. The increased flexibility of these residues without 3α,5α-THP binding suggests that they are more prone to conformational changes, which could influence MD-2’s interactions with other proteins or ligands. The stabilization effect of 3α,5α-THP highlights its potential role as a modulator of MD-2 dynamics, which is particularly relevant for understanding the protein’s function in the immune response.

### 3.8. 3α,5α-THP Demonstrates Strong Interaction Stability

The contact analysis revealed an average of approximately 1200 interactions between THP and MD-2, indicating a robust interaction stability within the binding pocket (Figure 5C). This high number of contacts underscores the significant role that THP plays in stabilizing the binding pocket, which is crucial for the effective modulation of MD-2 function.

### 3.9. 3α,5α-THP Binding Restricts Conformational Fluctuations

Visual analysis of the MD-2 + 3α,5α-THP complex further elucidated the stabilizing effect of 3α,5α-THP on the receptor structure. The binding of 3α,5α-THP appeared to restrict conformational fluctuations, providing a more rigid structure to the receptor (Supplementary Figures, Supplementary Movies M1 and M2). This observation suggests that 3α,5α-THP binding not only enhances the stability of MD-2 but may also contribute to its ability to engage effectively with downstream signaling partners in the immune response.

## 4. Discussion

This study provides new mechanistic insights into the inhibitory effects of the neurosteroid 3α,5α-THP on TLR4 signaling, potentially opening new avenues for modulating neuroimmune responses in conditions characterized by chronic inflammation, such as alcohol use disorder, depression, and neurodegenerative diseases [1,2,3,4,5,6,7,8,9,10,11,12]. We demonstrated that 3α,5α-THP interacts with the TLR4:MD-2 complex, specifically targeting the hydrophobic pocket of MD-2 which is also crucial for LPS binding. Our results showed that 3α,5α-THP competes with LPS, specifically its Lipid A moiety, for binding at overlapping sites on MD-2, effectively inhibiting the subsequent signaling cascade necessary for pro-inflammatory cytokine production.

This competitive inhibition was substantiated through molecular docking and molecular dynamic analysis as well as SPR competition assays, revealing that 3α,5α-THP binds to the same sites as the Lipid A moiety of LPS, with much higher affinity for MD-2 than Lipid A (nanomolar vs. micromolar). The present molecular docking results indicate that amino acids VAL 135, LEU61, ILE 63, and PHE151 are involved in 3α,5α-THP’s interaction with MD-2. However, we suggest that future mutational studies should be conducted for validation of these findings. Additionally, molecular dynamics results indicate that 3α,5α-THP binding significantly influences the structural dynamics of MD-2, enhancing its stability and potentially its functional capacity. This understanding of 3α,5α-THP:MD-2 binding dynamics contribute to our broader knowledge of the molecular mechanisms underlying TLR4 modulation and its functional implications in immune signaling pathways.

Previous studies have also provided a relevant comparison, demonstrating that synthetic agonists like neoseptin-3 can target the TLR4:MD-2 complex independent of traditional ligands [48]. Neoseptin-3 was shown to bind as a dimer within the same hydrophobic pocket of MD-2, contacting residues crucial for LPS interaction, and inducing a similar conformational change as LPS. Additionally, studies focusing on MD-2 as a druggable target supports the plausibility that this is the primary site of 3α,5α-THP action to inhibit TLR activation and underscores the therapeutic potential of targeting these molecular interactions [28,49,50].

In addition, we demonstrated that 3α,5α-THP does not significantly disrupt the interaction between TLR4 and MD-2 in the hippocampus of P rats. This observation could be attributed to several brain-specific mechanisms and the unique cellular environment of the hippocampus. Notably, in the brain, GABA_A_ receptor α2 subunit proteins are key ligands for TLR4 activation, which leads to the production of inflammatory mediators [29,51]. Interestingly, 3α,5α-THP inhibits the interaction between the α2 subunit and TLR4 in the P rat brain [23], suggesting that 3α,5α-THP might interact with TLR4/MD-2 differently in the brain compared with peripheral tissues. This could be due to distinct binding dynamics or modulatory effects, as well as possible variations in the expression levels or structural modifications of TLR4 and MD-2 within hippocampal cells. Moreover, although Lipid A has been found to cross the blood–brain barrier in rat models [52], we cannot be certain that Lipid A is present in the hippocampus of P rats. It appears that 3α,5α-THP inhibits the interaction between TLR4 and MD-2 only in RAW264.7 macrophage cells pretreated with LPS, but not without LPS [23].To further understand these complex interactions, more detailed studies are necessary.

Additionally, our study explored the effects of 3α,5α-THP on downstream TLR4 signaling components, particularly TIRAP and MyD88. We found that 3α,5α-THP disrupts the binding of TIRAP to MyD88, a critical step in myddosome assembly and subsequent NF-κB activation. Interestingly, while 3α,5α-THP inhibited TIRAP:MyD88 binding, it did not affect the interaction of MyD88 with other myddosome proteins such as IRAK4 or IRAK1, nor did it alter their expression levels. Additionally, 3α,5α-THP had no effect on the expression levels of other MyD88:TIRAP-associated proteins such as BTK and CLIP-170. This selective modulation highlights 3α,5α-THP’s capacity for targeted interference within TLR signaling pathways, potentially allowing controlled immune response modulation with minimized systemic side effects.

Currently, there are efforts underway in targeting TIRAP as a therapeutic approach for modulating TLR signaling. Drawing parallels with the o-vanillin study, which demonstrated selective TLR2 inhibition by covalently binding to the TIR domain of TIRAP, our findings with 3α,5α-THP indicate that small molecules can specifically disrupt critical protein–protein interactions within TLR pathways, potentially preventing key steps in signal propagation [53]. This activity might be mediated through direct binding or conformational changes in key signaling proteins, warranting further exploration using advanced techniques such as NMR spectroscopy and X-ray crystallography. Previous studies have demonstrated that 3α,5α-THP disrupts the binding of MyD88 to TLR4 without affecting the interaction between TRIF and TLR4 [26], suggesting a possible direct interaction of 3α,5α-THP with TIRAP. Other research groups have shown that directly targeting TIRAP has been an effective way of decreasing TLR4 signaling. For example, a decoy peptide, targeted for the TLR4 TIR domain was found to bind TIRAP and inhibited TIRAP-involved TLR signaling. These studies provide further insight into the possibility of directing compounds, such as 3α,5α-THP towards the cytosolic component of TLR4 signaling [54]. Thus, further investigation of 3α,5α-THP or other neurosteroid interactions with TIRAP may lead to novel treatments for inflammatory conditions. In addition, the necessity of TLR4 TIR domain dimerization for stable TIRAP binding underscores the significance of TLR4 dimerization in TIRAP-related signaling pathways [14,15,17,55]. Therefore, disruption of TLR4 dimerization through inhibition of LPS binding, as described above, may be the primary cause of the observed inhibition of TIRAP:MyD88 binding.

The significant reduction in key inflammatory mediators HMGB1 and IL-1β within the hippocampus of P rats, as observed in our experiments following administration of 3α,5α-THP, offers profound insights into its anti-inflammatory mechanisms in neuro-inflammatory conditions. By substantially decreasing levels of these cytokines, which are critical in the propagation of inflammation through TLR4 signaling pathways, 3α,5α-THP demonstrates its capability to modulate neuroimmune responses. The reduction in HMGB1 and IL-1β production, along with the inhibition of both TIRAP:MyD88 and Lipid A:MD-2 binding by 3α,5α-THP, underscores its potential as a therapeutic agent in conditions where TLR4-mediated neuroinflammation plays a central role. The multiple effects of 3α,5α-THP on TLR4 signaling pathways suggest that further development of neurosteroids could be a promising strategy for mitigating excessive inflammatory responses while preserving essential innate immune functions necessary for pathogen defense.

## 5. Conclusions

This study demonstrates a novel mechanism of 3α,5α-THP action on TLR-4 mediated inflammation. 3α,5α-THP interacts with the TLR4:MD-2 complex by high affinity binding within the hydrophobic pocket of MD-2 which is also crucial for LPS binding. 3α,5α-THP competes with LPS, specifically its lipid A moiety, for binding at overlapping sites on MD-2, stabilizing the molecule and effectively inhibiting the subsequent TLR4 signaling cascade that produces pro-inflammatory modulators. This work highlights 3α,5α-THP as a promising candidate for modulating neuroimmune responses through selective inhibition of key protein–protein and protein–pathogen interactions within the TLR4 signaling pathway. Future studies should focus on further characterizing the precise molecular mechanisms by which 3α,5α-THP exerts its effects and establishing the structural requirements for these interactions, enhancing its therapeutic application.

## Figures and Tables

**Table 1:** **Comparison of protein expression in Vehicle- and 3α,5α-THP-treated P rats.** 3α,5α-THP does not alter the expression of key TLR4-associated proteins. To further clarify the effects of 3α,5α-THP on TLR4 signaling, various proteins involved in the TLR4 pathway were tested. Male and female P rats were treated with 3α,5α-THP (15 mg/kg; 30 min) or vehicle (45% *w*/*v* 2-hydroxypropyl-β-cyclodextrin; 30 min), as previously described. There were no significant sex or treatment effects in protein expression in the hippocampi of P rats of the listed proteins.

Protein	Sex	Vehicle (Mean O.D. Normalized to *β*-Actin)	3α,5α-THP (Mean O.D. Normalized to *β*-Actin)	Mean Difference (×10^6^ ± SEM)	Treatment *p* Value (2-Way ANOVA)
**TLR4**	Male	4.42 × 10^6^	3.59 × 10^6^	−0.83 ± 0.93	0.0941 (ns)
	Female	3.97 × 10^6^	3.19 × 10^6^	−0.79 ± 0.37	
**IRAK4**	Male	5.80 × 10^6^	5.38 × 10^6^	−0.41 ± 0.16	0.2136 (ns)
	Female	4.78 × 10^6^	3.86 × 10^6^	−0.92 ± 0.45	
**IRAK1**	Male	2.04 × 10^6^	2.21 × 10^6^	0.17 ± 0.61	0.8825 (ns)
	Female	1.58 × 10^6^	1.34 × 10^6^	−0.24 ± 0.28	
**BTK**	Male	5.92 × 10^6^	6.12 × 10^6^	0.19 ± 0.52	0.2640 (ns)
	Female	1.52 × 10^5^	1.25 × 10^5^	−0.03 ± 0.03	
**CLIP170**	Male	4.29 × 10^6^	4.90 × 10^6^	0.61 ± 0.27	0.0717 (ns)
	Female	5.63 × 10^5^	5.03 × 10^5^	−0.06 ± 0.03	

## Data Availability

Data are available upon reasonable request.

## Supplementary Materials

The following supporting information can be downloaded at: https://www.mdpi.com/article/10.3390/biom14111441/s1, Table S1: Antibodies, Raw data for western blots, Molecular Dynamics Movie M1, Molecular Dynamics Movie M2, Molecular dynamics movie legends (ppt).
