# Supplementary material for: Novel Inhibitory Actions of Neuroactive Steroid [3α,5α]-3-Hydroxypregnan-20-One on Toll-like Receptor 4-Dependent Neuroimmune Signaling
Source: Biomolecules. 2024 Nov 13;14(11):1441. doi: 10.3390/biom14111441 (PMC11591752; doi:10.3390/biom14111441)
Supplement: Supplementary file 1 [file biomolecules-14-01441-s001.zip › 241106_Supplement_Table.pdf]

Supporting information

Target	Catalog No.	Supplier	Clonality	Host	Dilution
**IL-1β**	RM1009	Abcam. Waltham, MA, USA	Multiclonal	Rabbit	1:1000
**HMGB1**	SC-56698	Santa Cruz Biotechnology. Santa Cruz ,CA,USA	Monoclonal	Mouse	1:800
**MyD88**	AF3109	R&D Systems. Minneapolis, MN, USA	Polyclonal	Goat	1:200
**MyD88**	4283	Cell Signaling Technology. Danvers, MA, USA	Polyclonal	Rabbit	1:500
**TIRAP**	LS-C747701	LSBio. Shirley, MA, USA	Polyclonal	Rabbit	1:500
**IRAK4**	EPR24506-71	Abcam. Waltham, MA, USA	Monoclonal	Rabbit	1:500
**IRAK1**	SC-5288	Santa Cruz Biotechnology. Santa Cruz ,CA,USA	Monoclonal	Mouse	1:1000
**CLIP170**	8977	Cell Signaling Technology. Danvers, MA, USA	Polyclonal	Rabbit	1:500
**BTK**	SC-28387	Santa Cruz Biotechnology. Santa Cruz ,CA,USA	Monoclonal	Mouse	1:500
**TLR4**	SC-293072	Santa Cruz Biotechnology. Santa Cruz ,CA,USA	Monoclonal	Mouse	1:800
**TLR7**	NBP2-24906SS	Novus Biologicals. Centennial, CO,USA	Polyclonal	Rabbit	1:500
**MD-2**	GTX85517	GeneTex. Irvine, CA, USA	Polyclonal	Rabbit	1:500
**B-Actin**	6609-1-1g	Proteintech Group. Rosemont, IL, USA	Monoclonal	Mouse	1:5,000

Supplement Table S1. Table contains primary antibodies and information regarding their use in co-immunoprecipitation and immunoblotting.
