# Supplementary material for: Novel Inhibitory Actions of Neuroactive Steroid [3α,5α]-3-Hydroxypregnan-20-One on Toll-like Receptor 4-Dependent Neuroimmune Signaling
Source: Biomolecules. 2024 Nov 13;14(11):1441. doi: 10.3390/biom14111441 (PMC11591752; doi:10.3390/biom14111441)

Figure 1 Western Blots

Figure 1A Males: **IL-1β** & **β-actin**

IL-1β

Vehicle **3α,5α-THP**

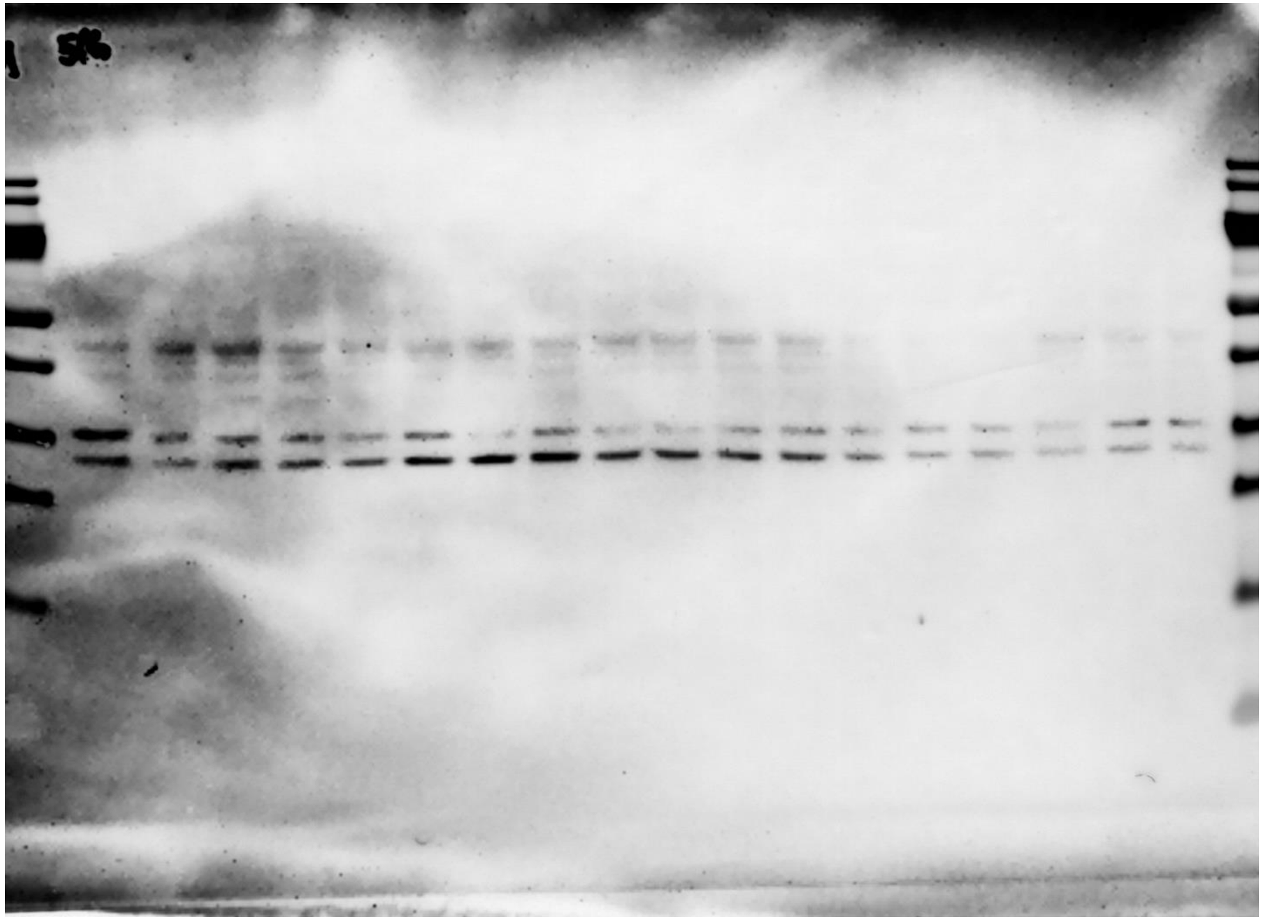

β-actin

Vehicle **3α,5α-THP**

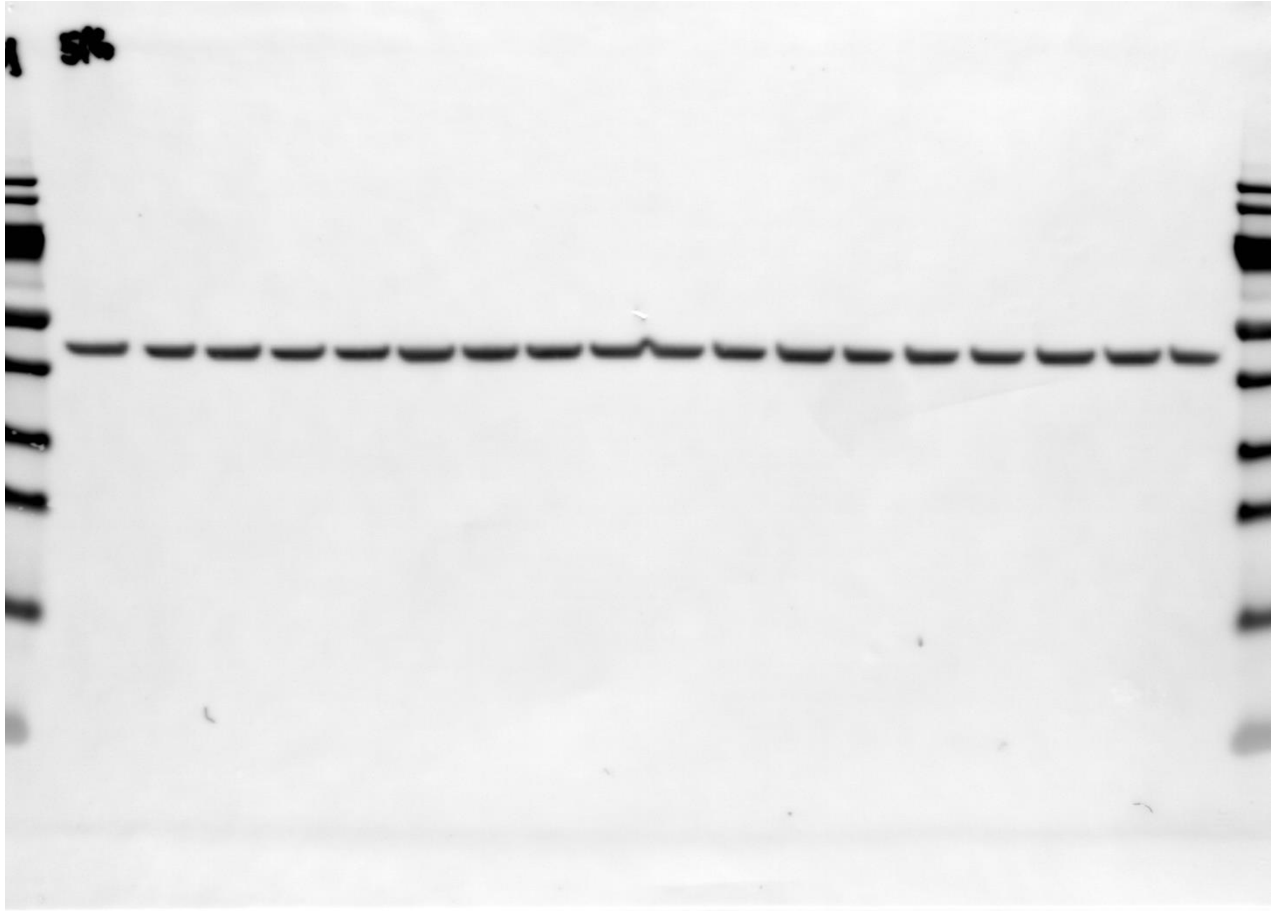

Figure 1A Females: **IL-1 β** & **β-actin**

IL-1β

Vehicle **3α,5α-THP**

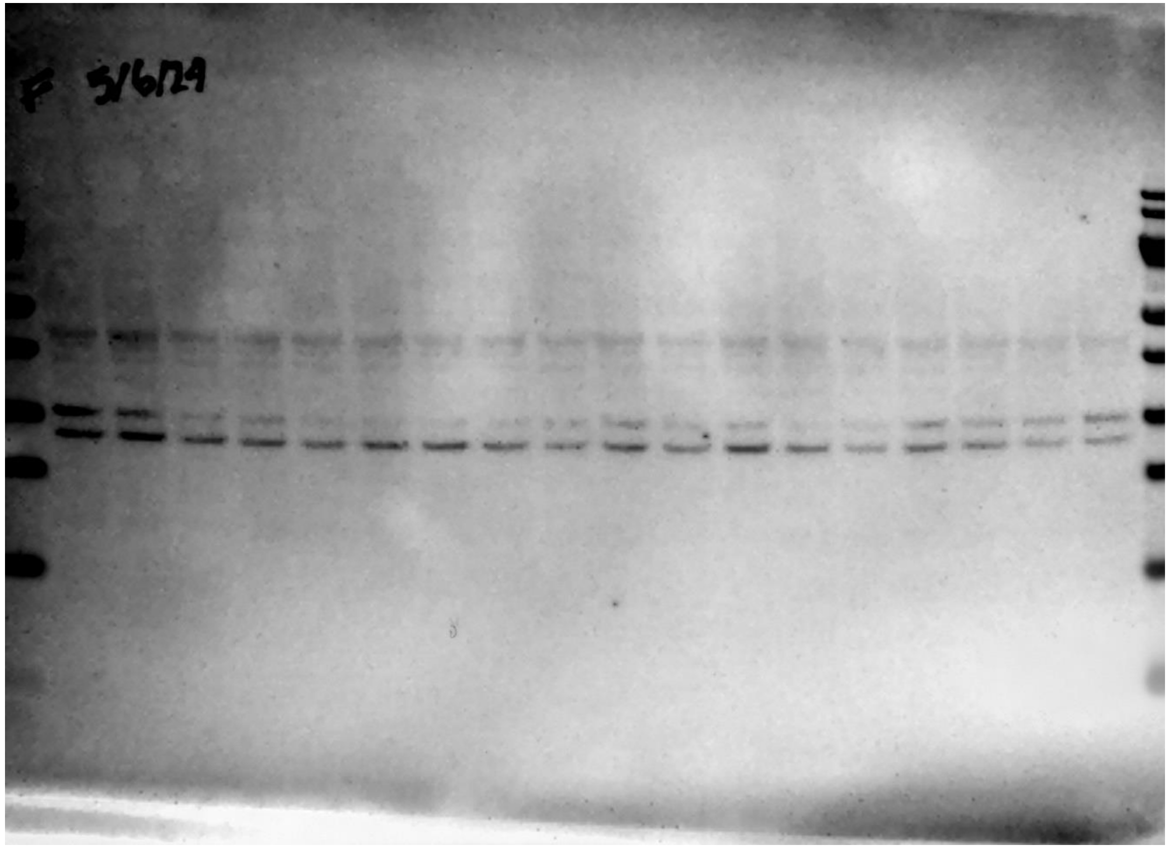

β-actin

Vehicle **3α,5α-THP**

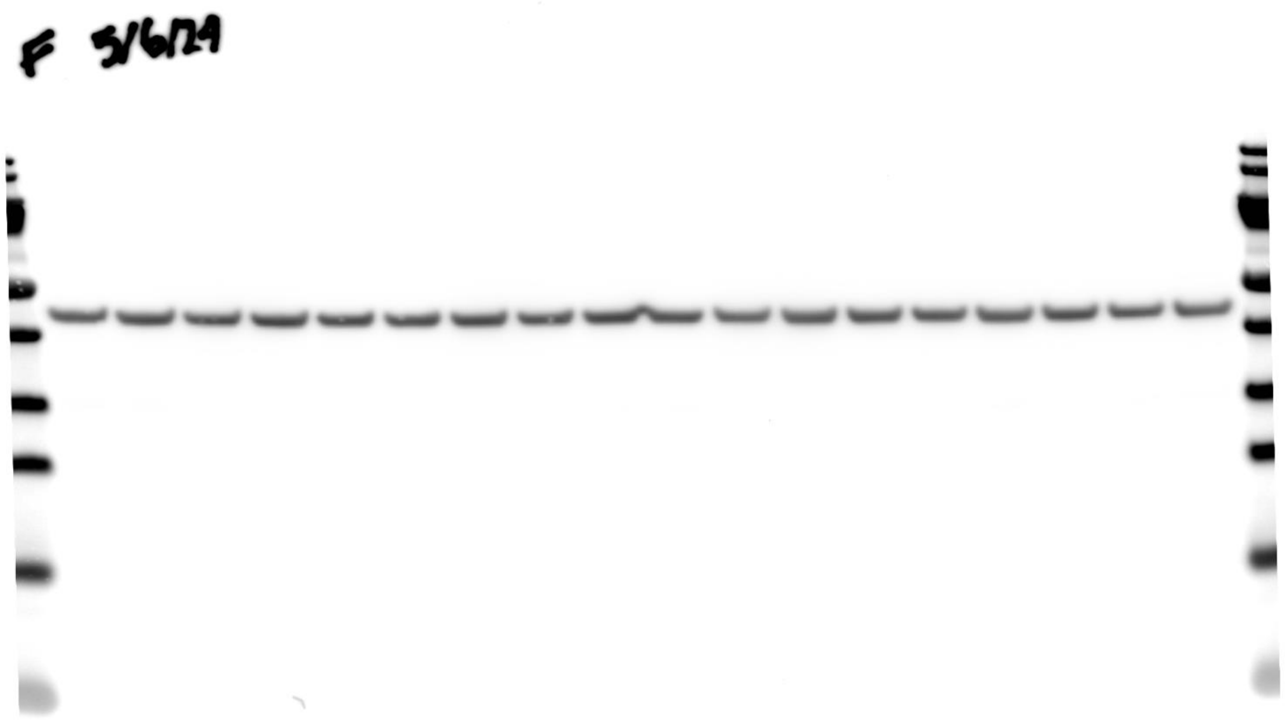

Figure 1B Males: **HMGB1** & **β-actin**

HMGB1

Vehicle | 3α,5α-THP

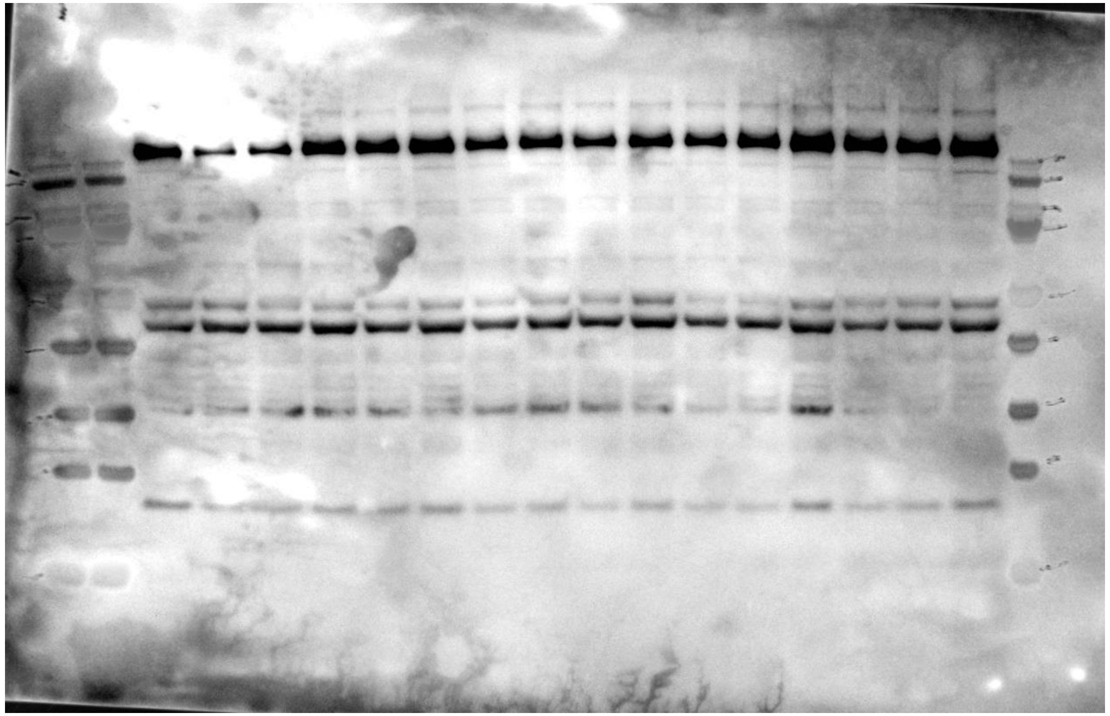

β-actin

Vehicle | 3α,5α-THP

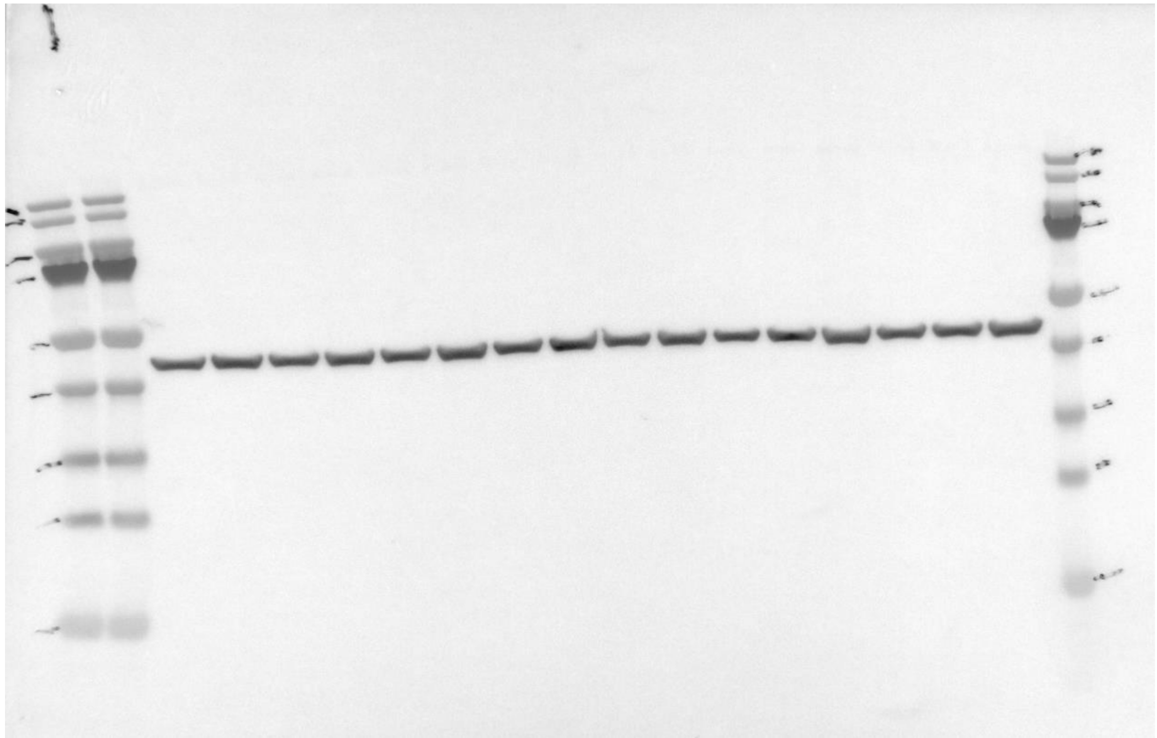

Figure 1B Females: **HMGB1** & **β-actin**

HMGB1

Vehicle **3α,5α-THP**

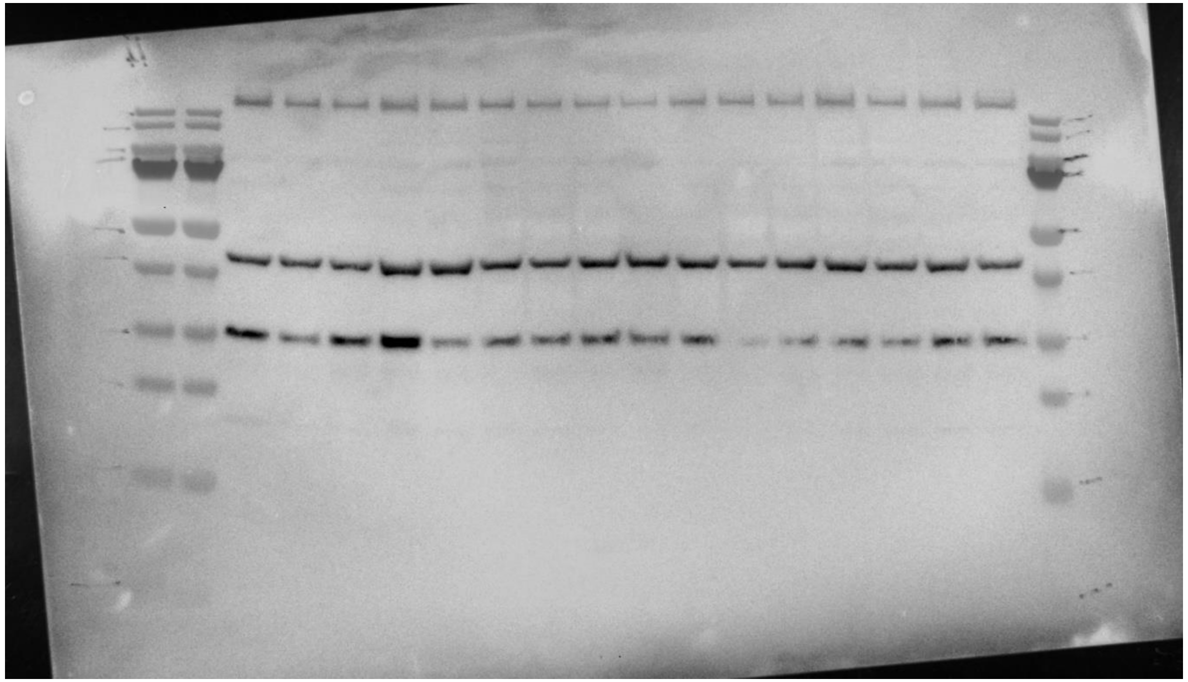

β-actin

Vehicle **3α,5α-THP**

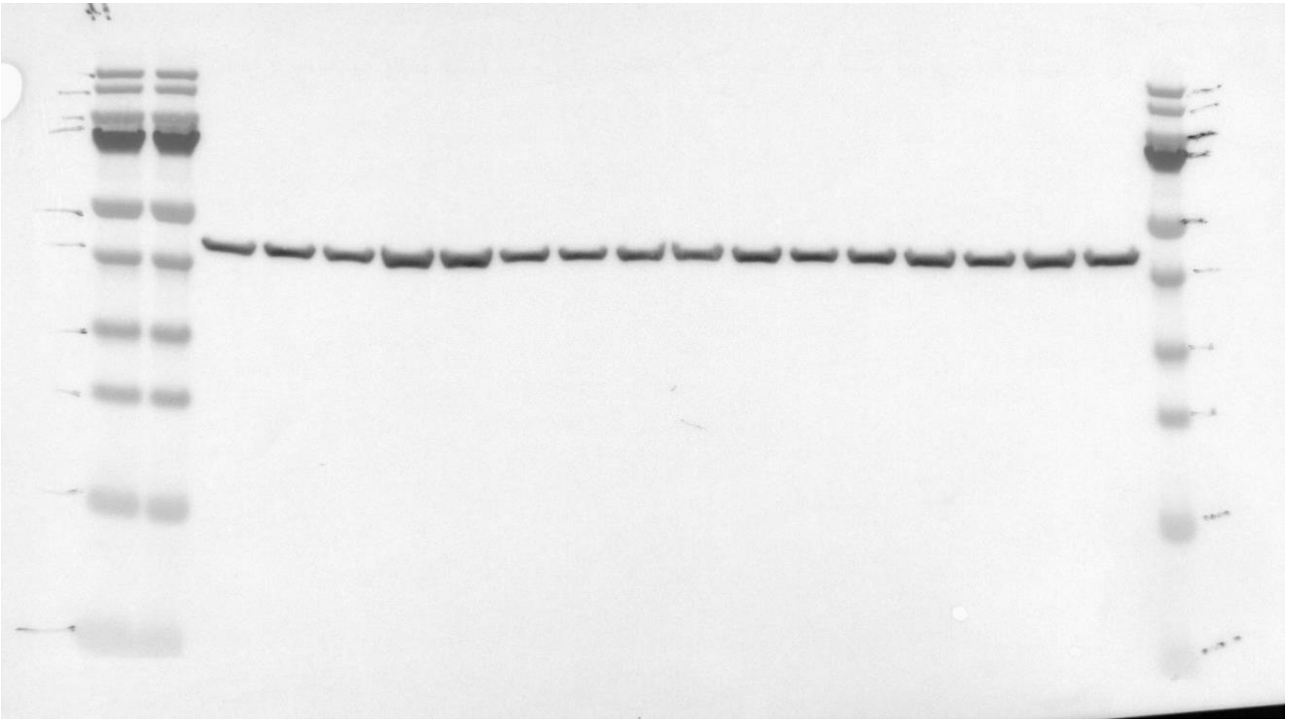

Figure 2 Western Blots

Figure 2A Males: Co-IP **TIRAP** and **MyD88**

TIRAP

Vehicle **3α,5α-THP**

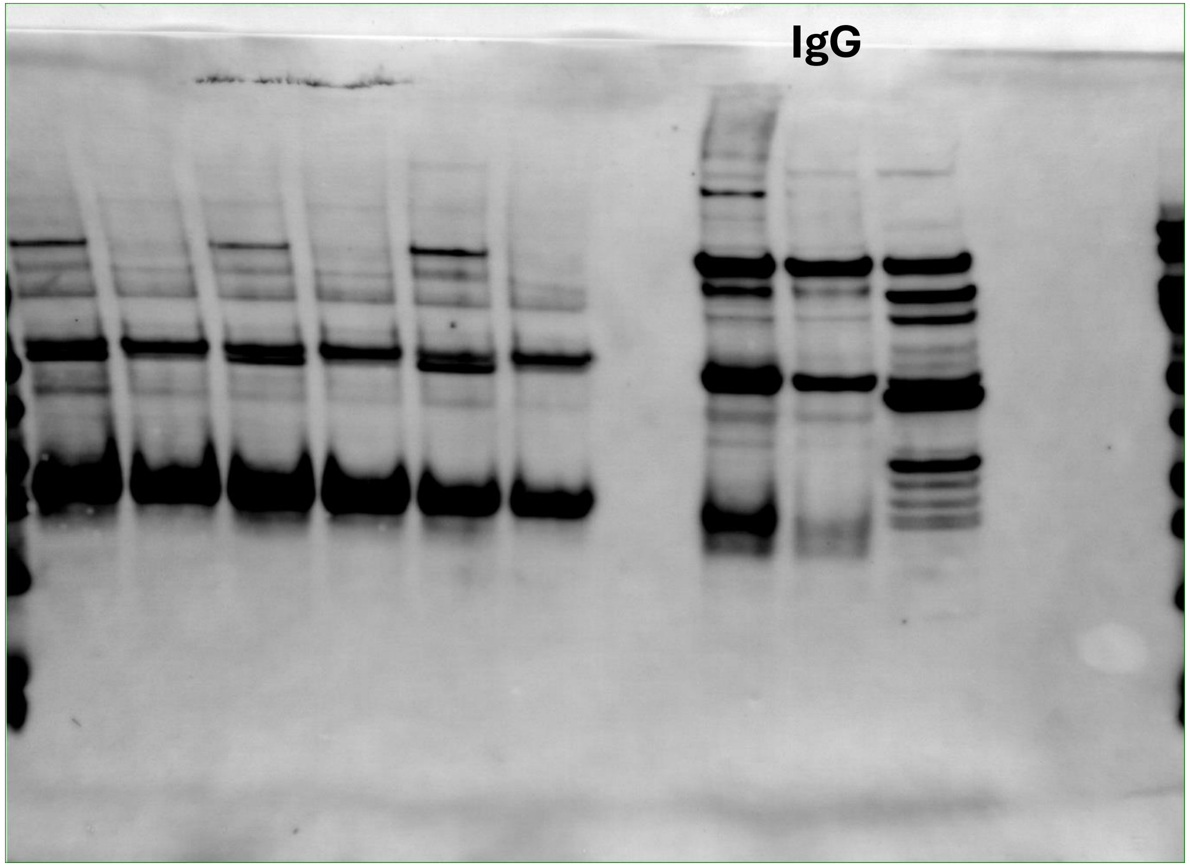

MyD88

Vehicle **3α,5α-THP**

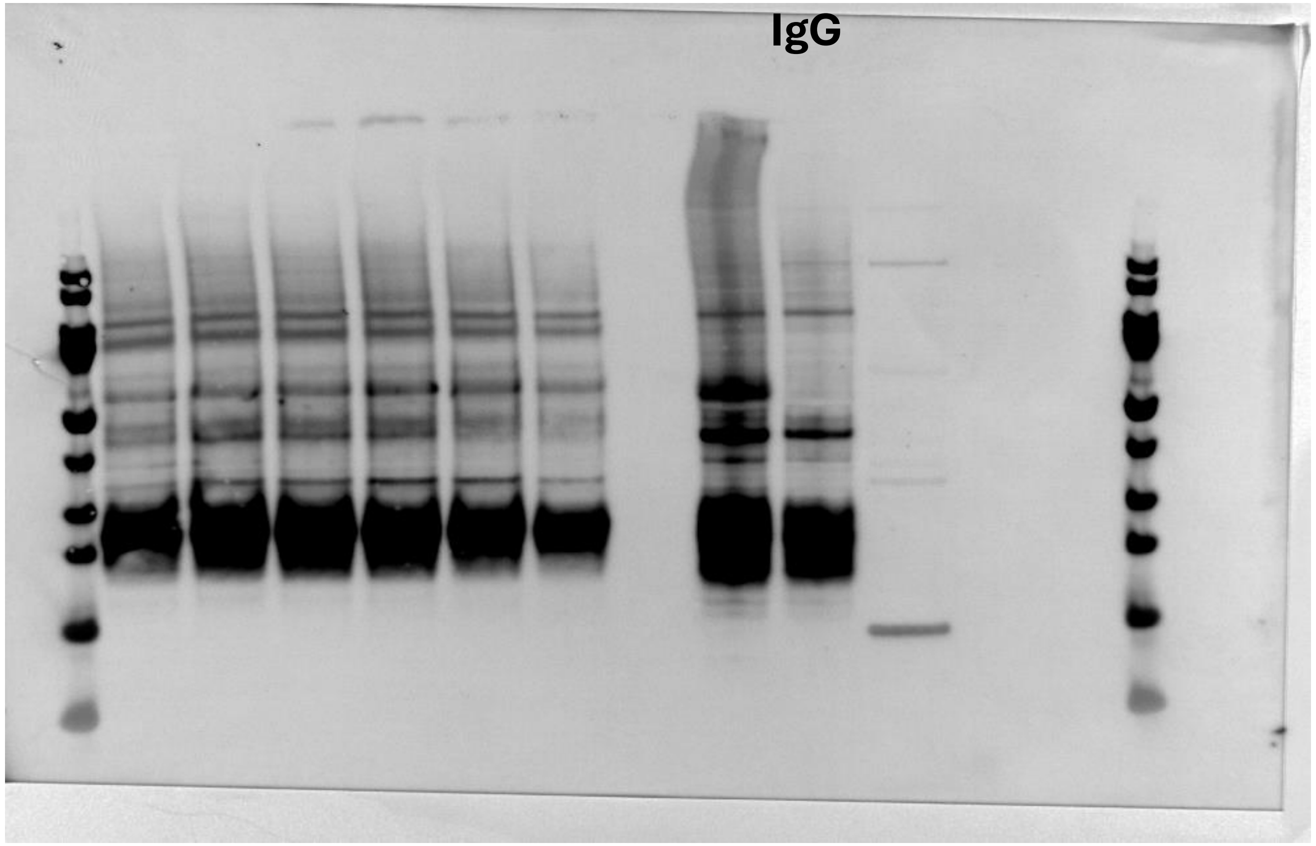

Figure 2C Males: Co-IP **IRAK4** and **MyD88**

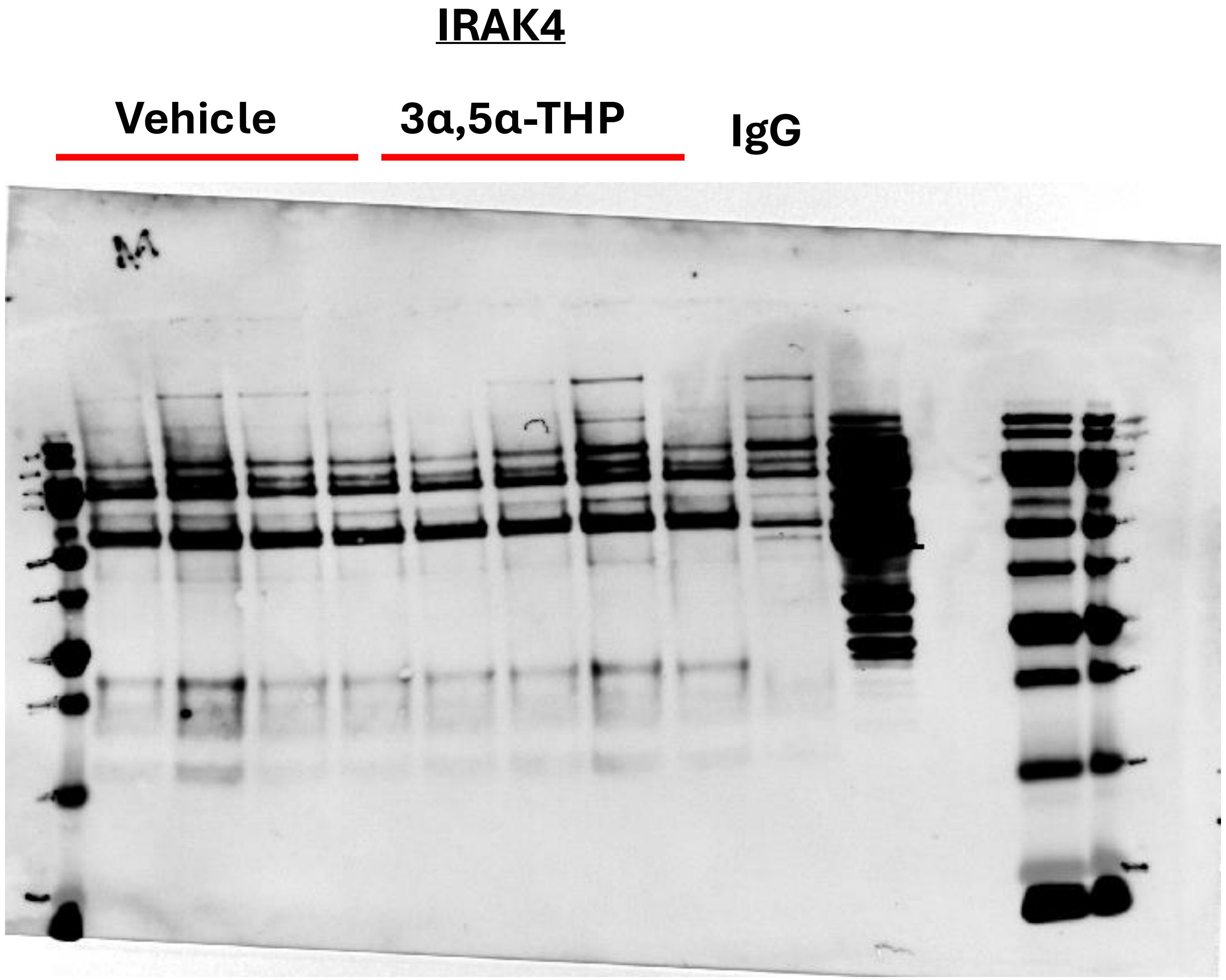

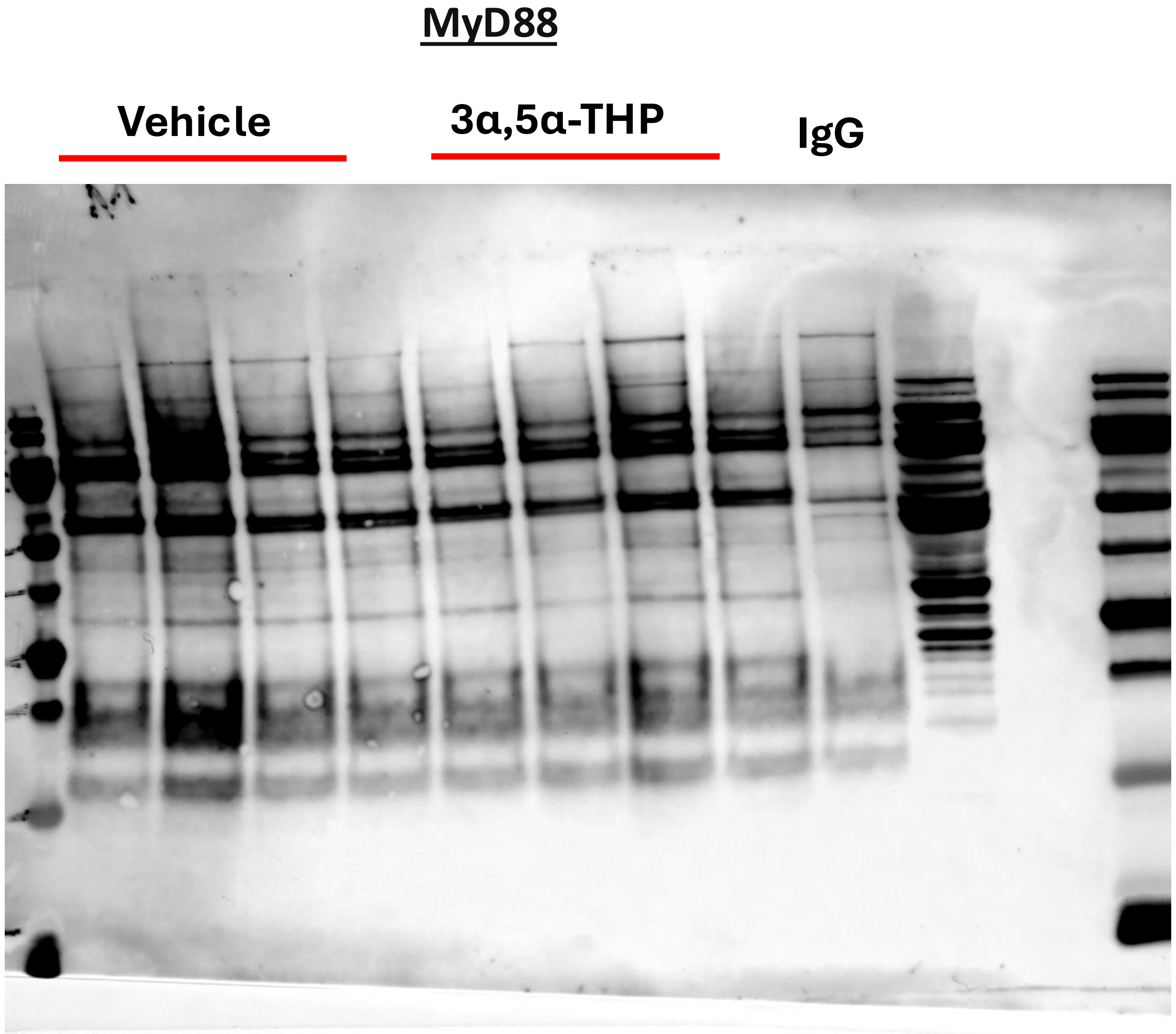

Figure 2E Males: Co-IP **IRAK1** and **MyD88**

IRAK1

Vehicle **3α,5α-THP** **IgG**

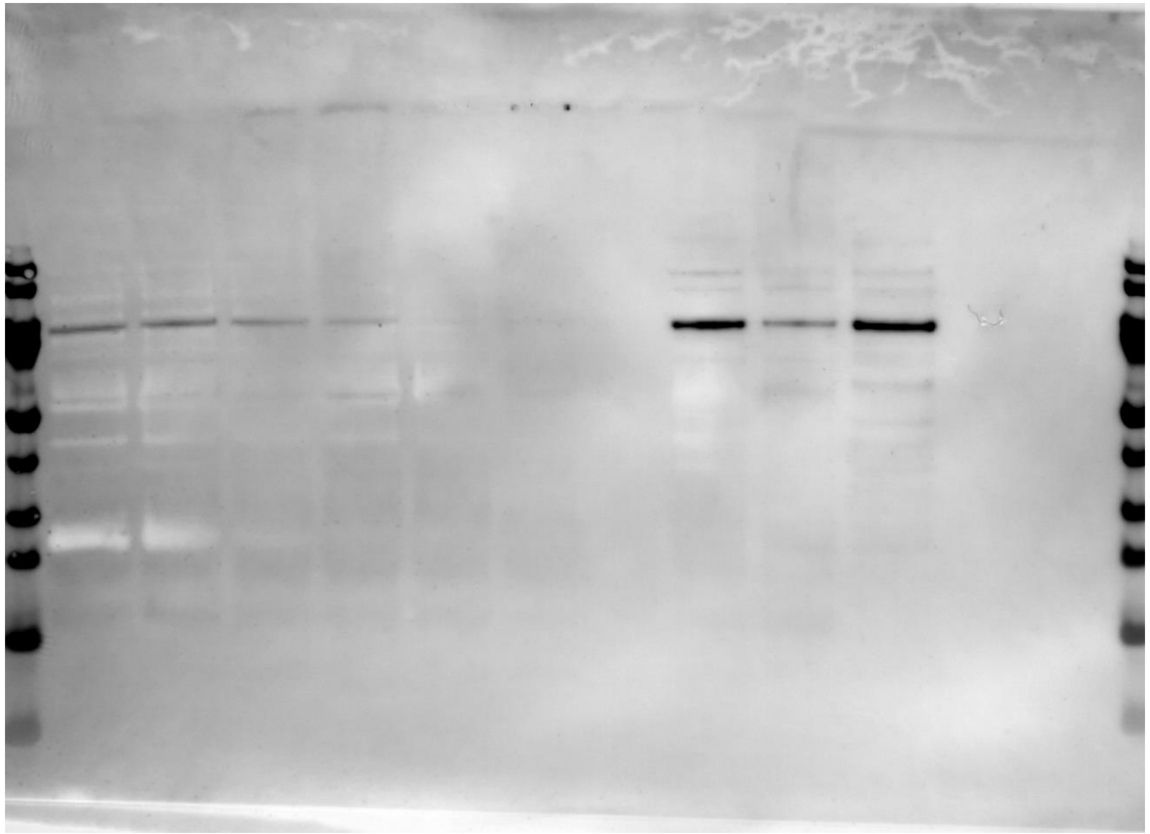

MyD88

Vehicle **3α,5α-THP** **IgG**

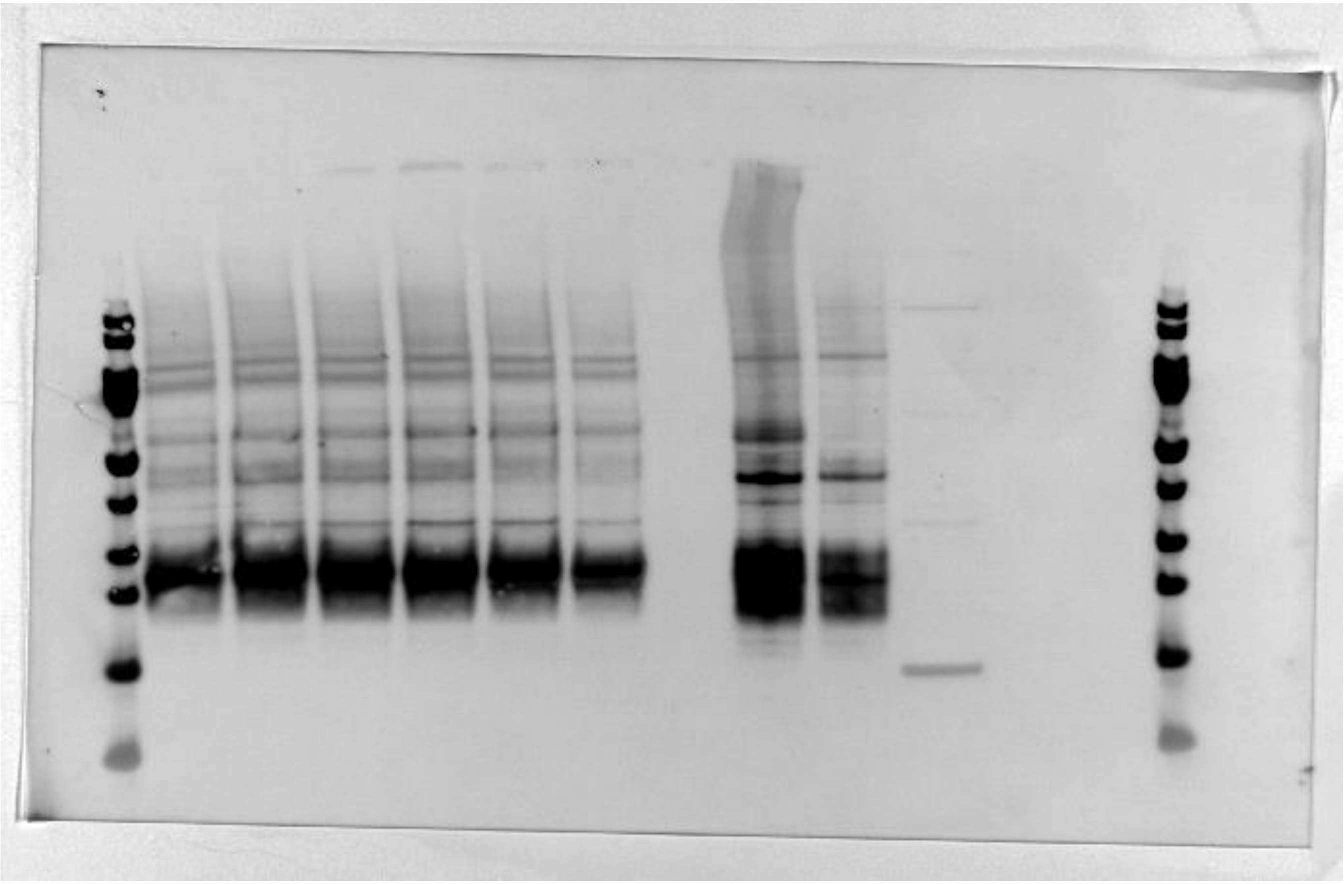

Figure 2B Females: Co-IP **TIRAP** and **MyD88**

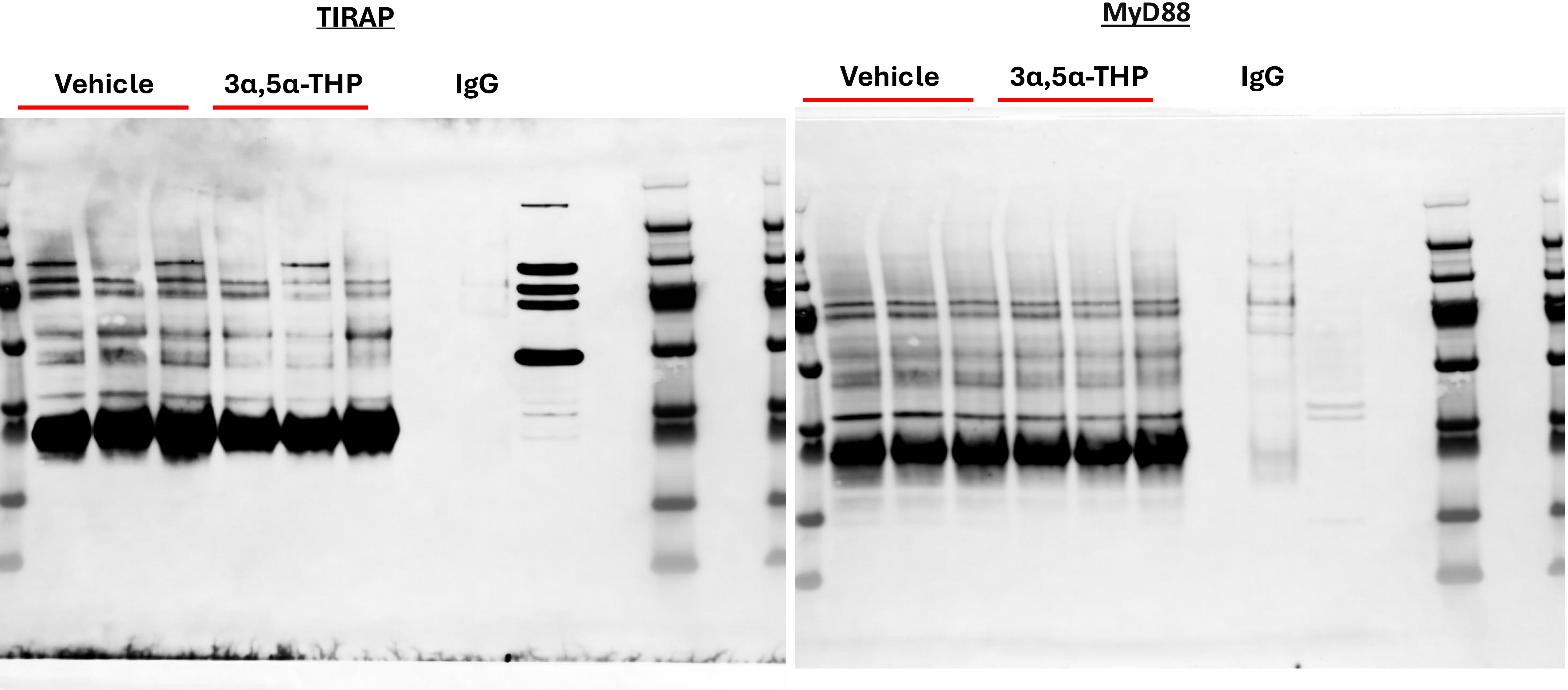

Figure 2D Females: Co-IP **IRAK4** and **MyD88**

IRAK4

Vehicle **3α,5α-THP** **IgG**

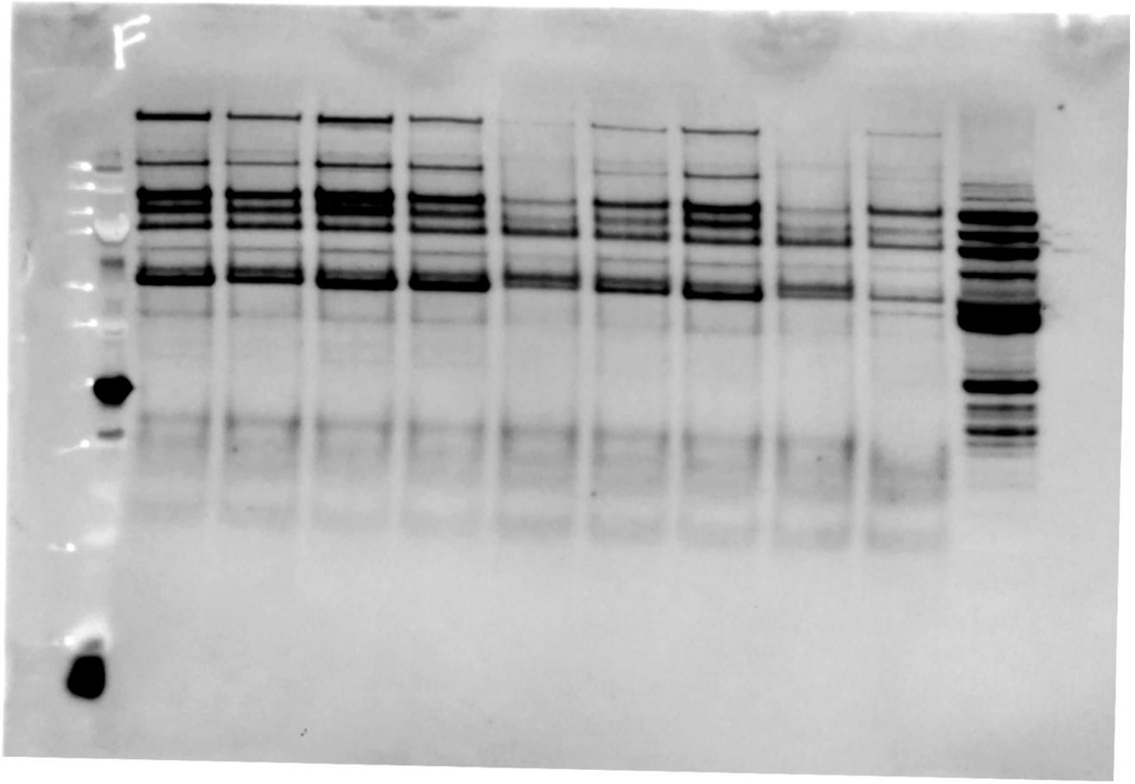

MyD88

Vehicle **3α,5α-THP** **IgG**

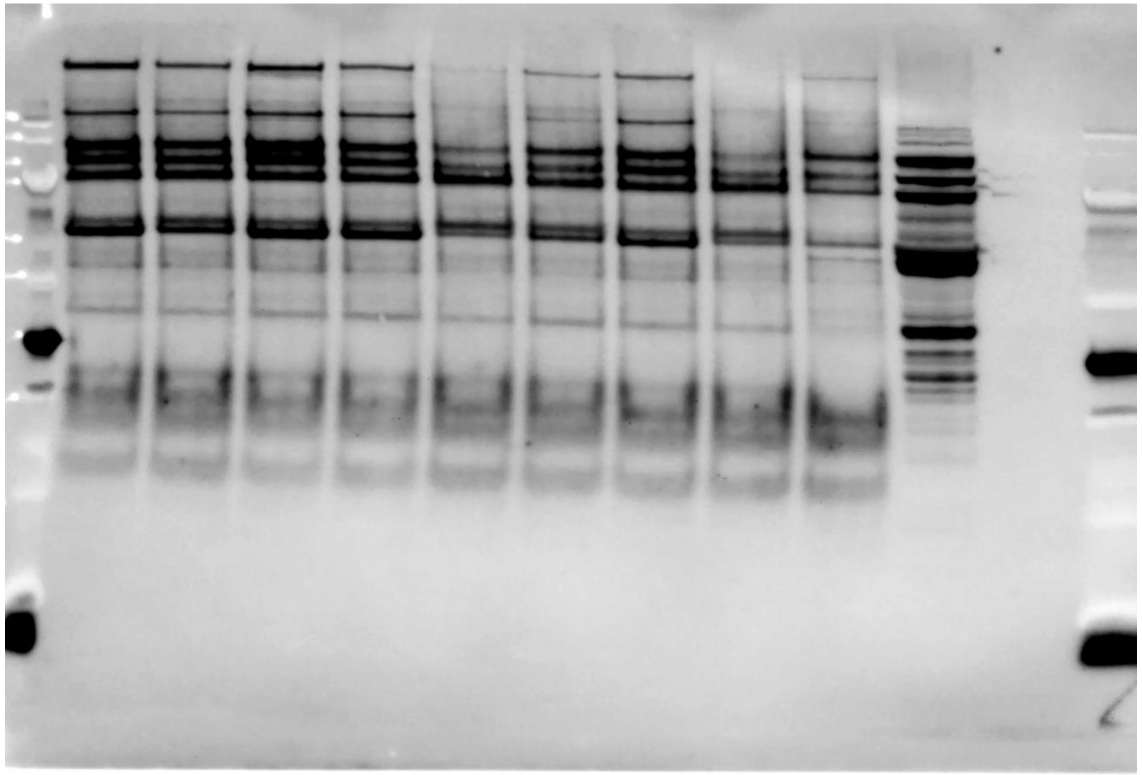

Figure 2E Females: Co-IP **IRAK1** and **MyD88**

IRAK1

Vehicle 3α,5α-THP IgG

MyD88

Vehicle 3α,5α-THP IgG

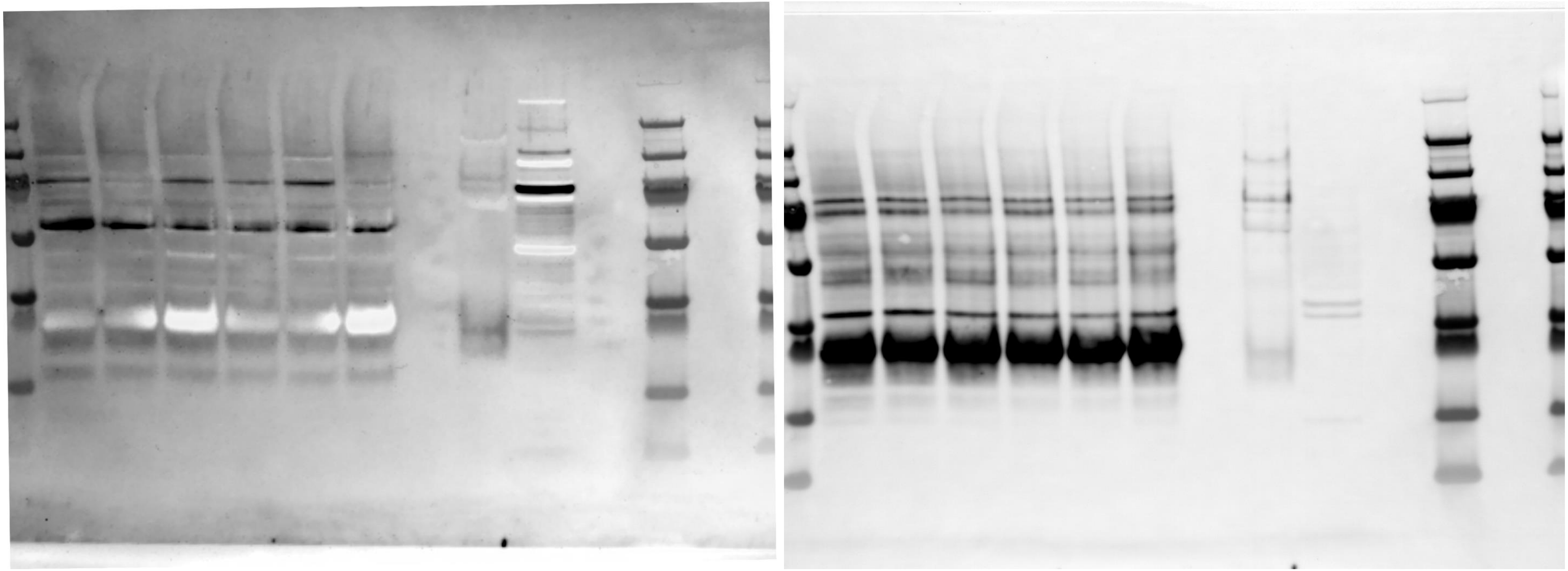

Figure 2G Males: **MyD88** & **β-actin**

MyD88

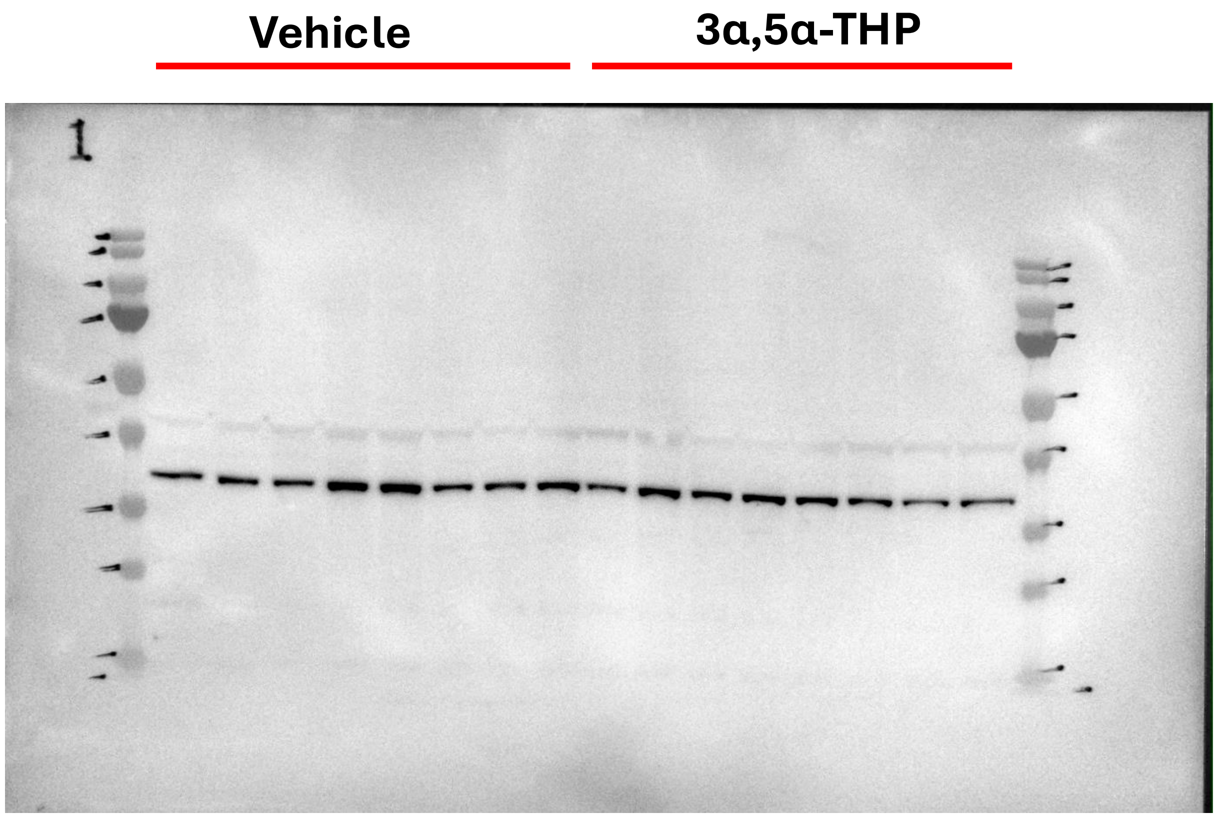

β-actin

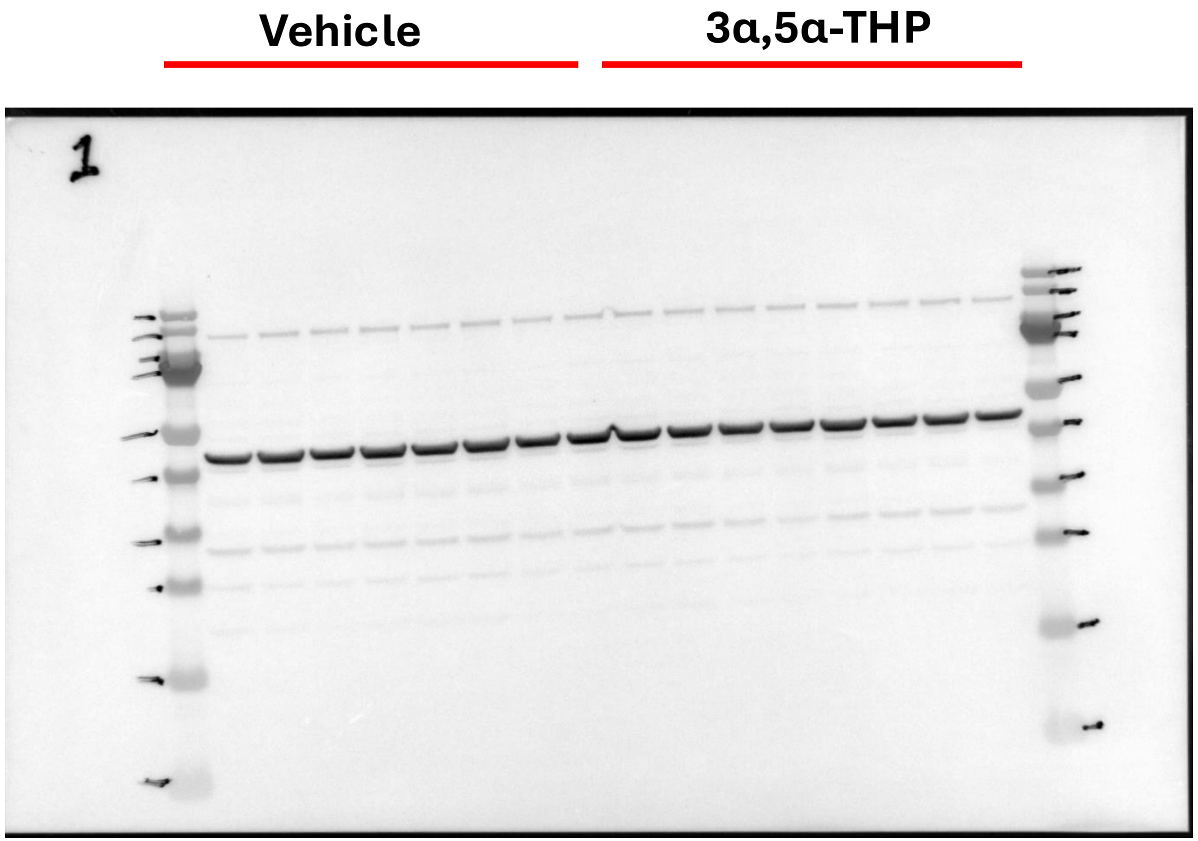

Figure 2G Females: **MyD88** & **β-actin**

MyD88

Vehicle **3α,5α-THP**

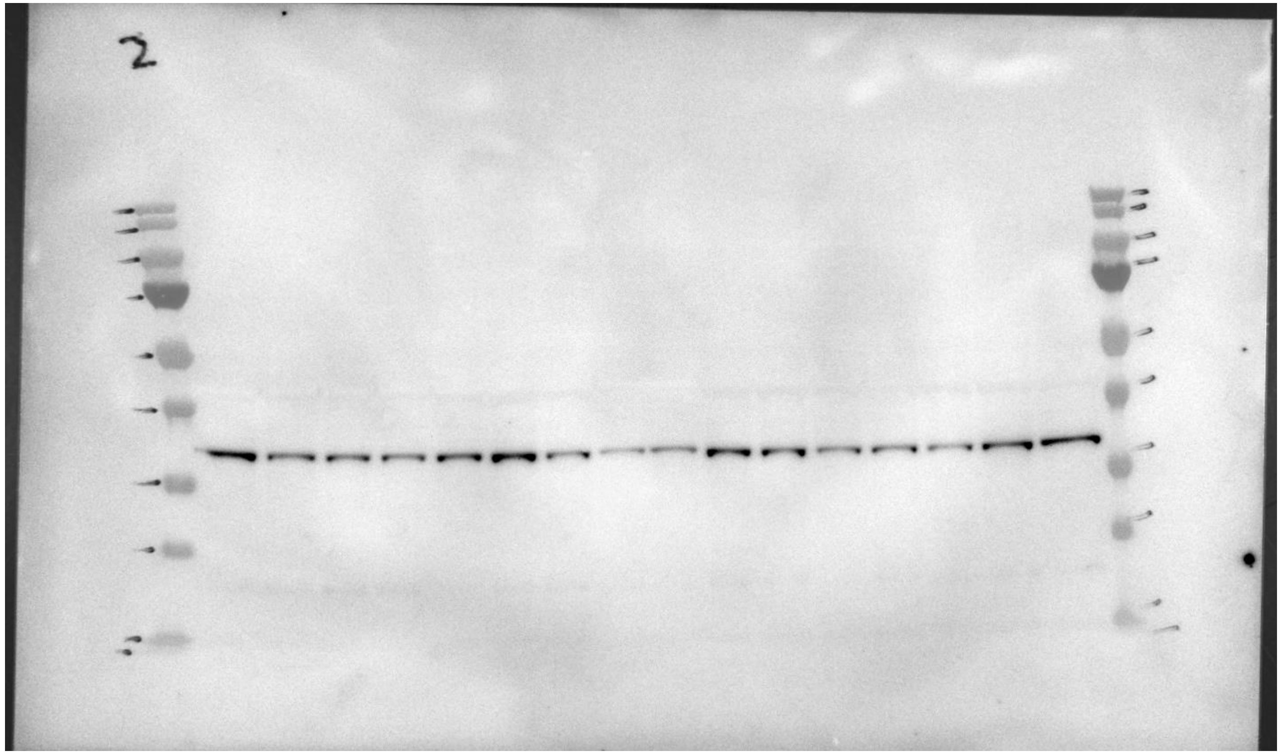

β-actin

Vehicle **3α,5α-THP**

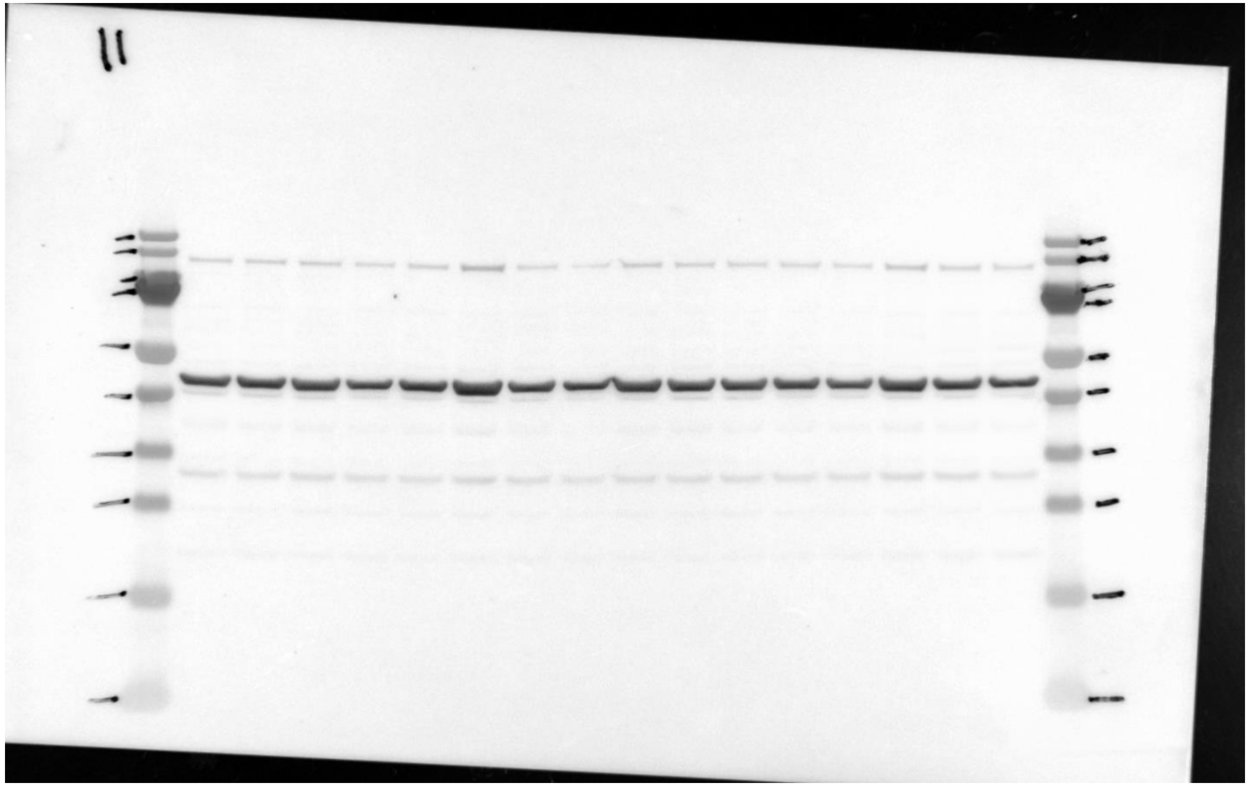

Figure 2H Males: **TIRAP** & **β-actin**

TIRAP

Vehicle **3α,5α-THP**

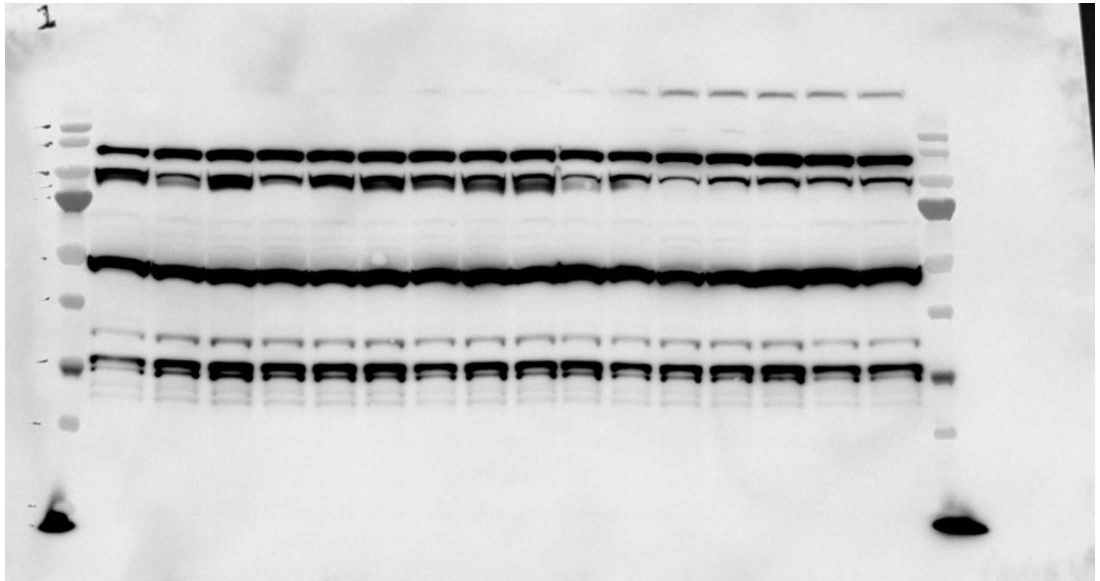

β-actin

Vehicle **3α,5α-THP**

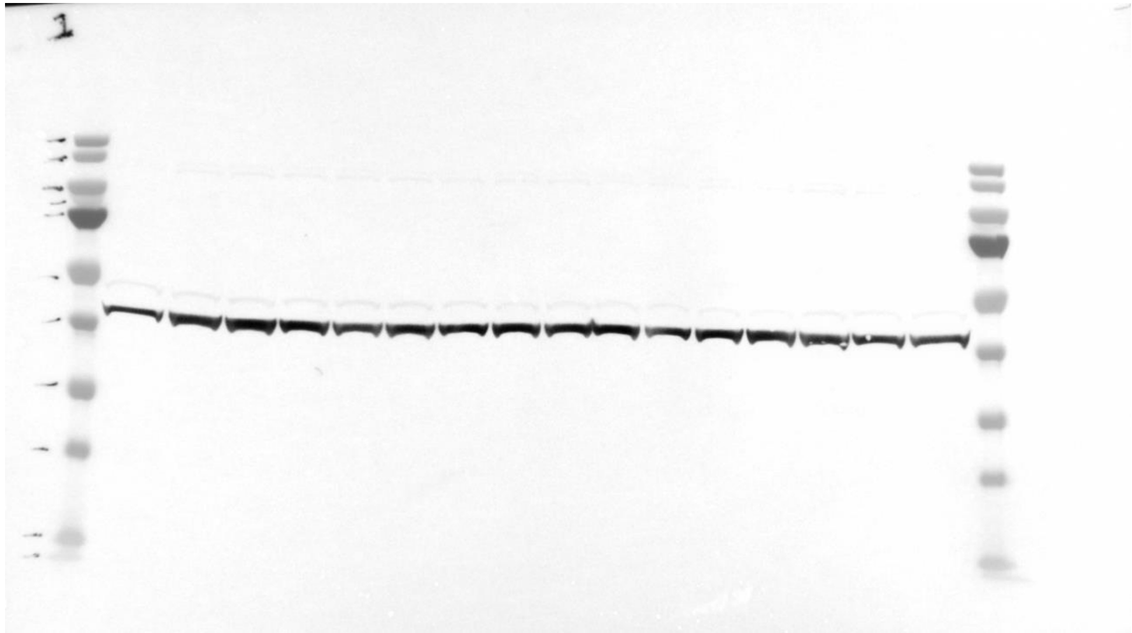

Figure 2H Females **TIRAP** & **β-actin**

TIRAP

Vehicle **3α,5α-THP**

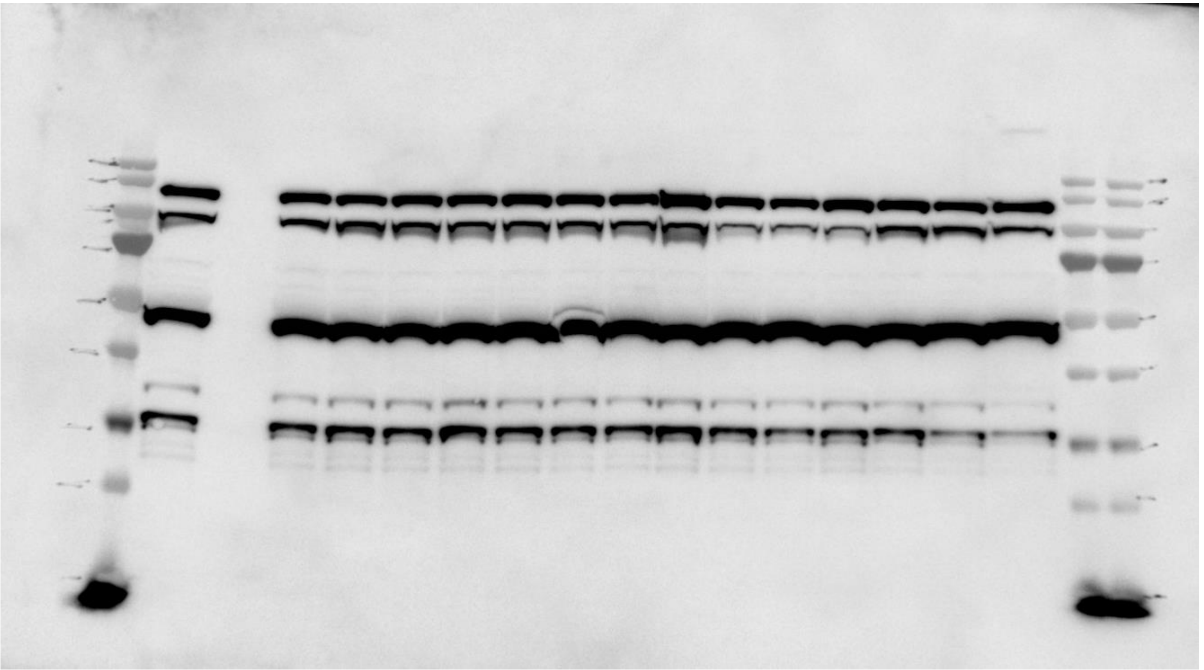

β-actin

Vehicle **3α,5α-THP**

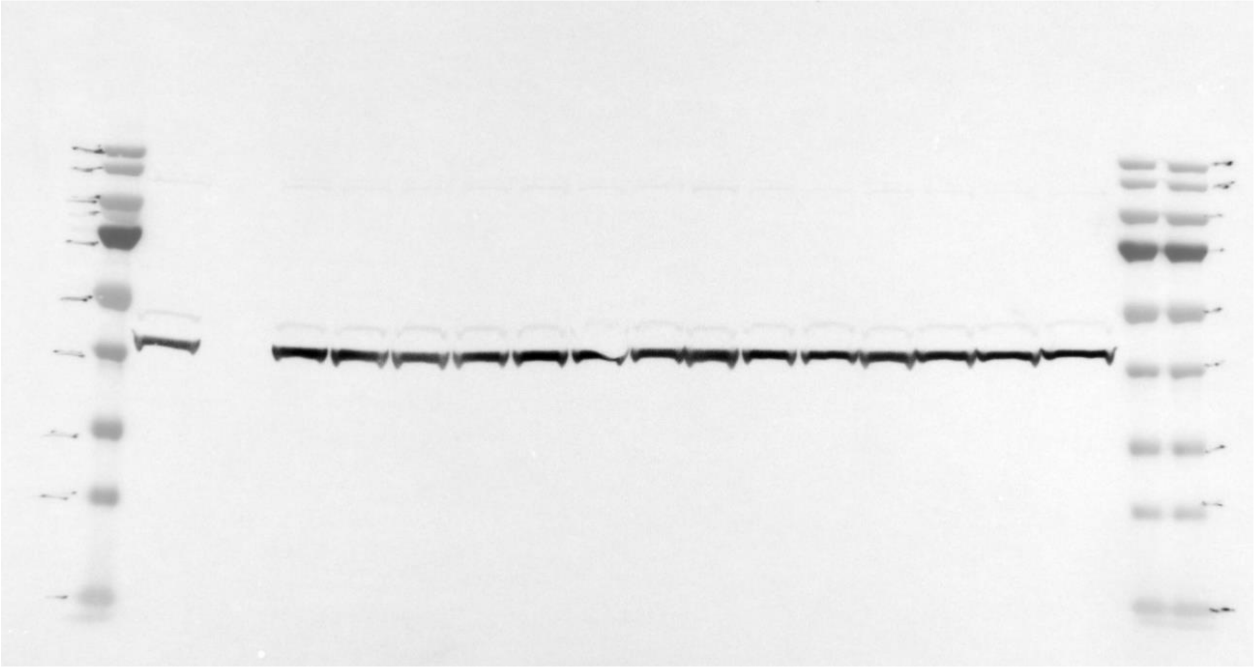

Table 1 Western Blots

Females: TLR4 & β-actin

TLR4

Vehicle **3α,5α-THP**

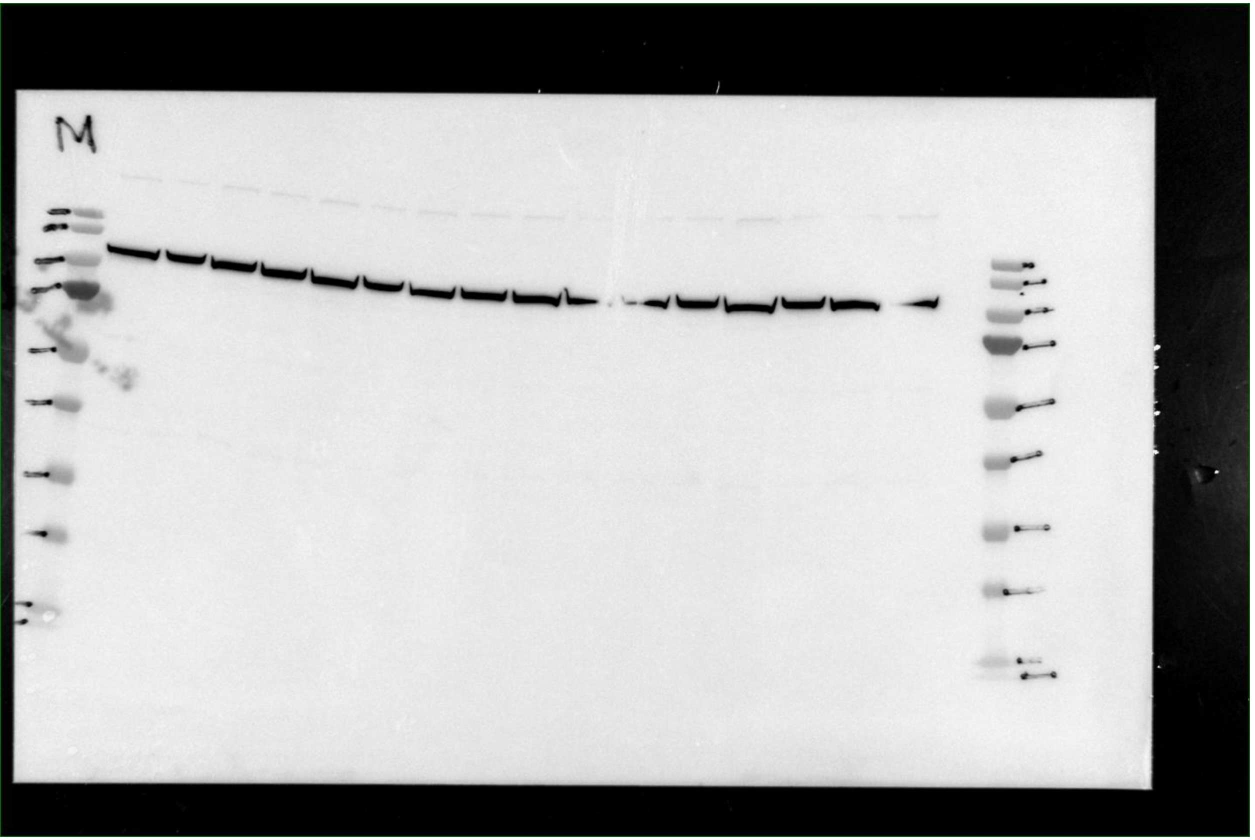

β-actin

Vehicle **3α,5α-THP**

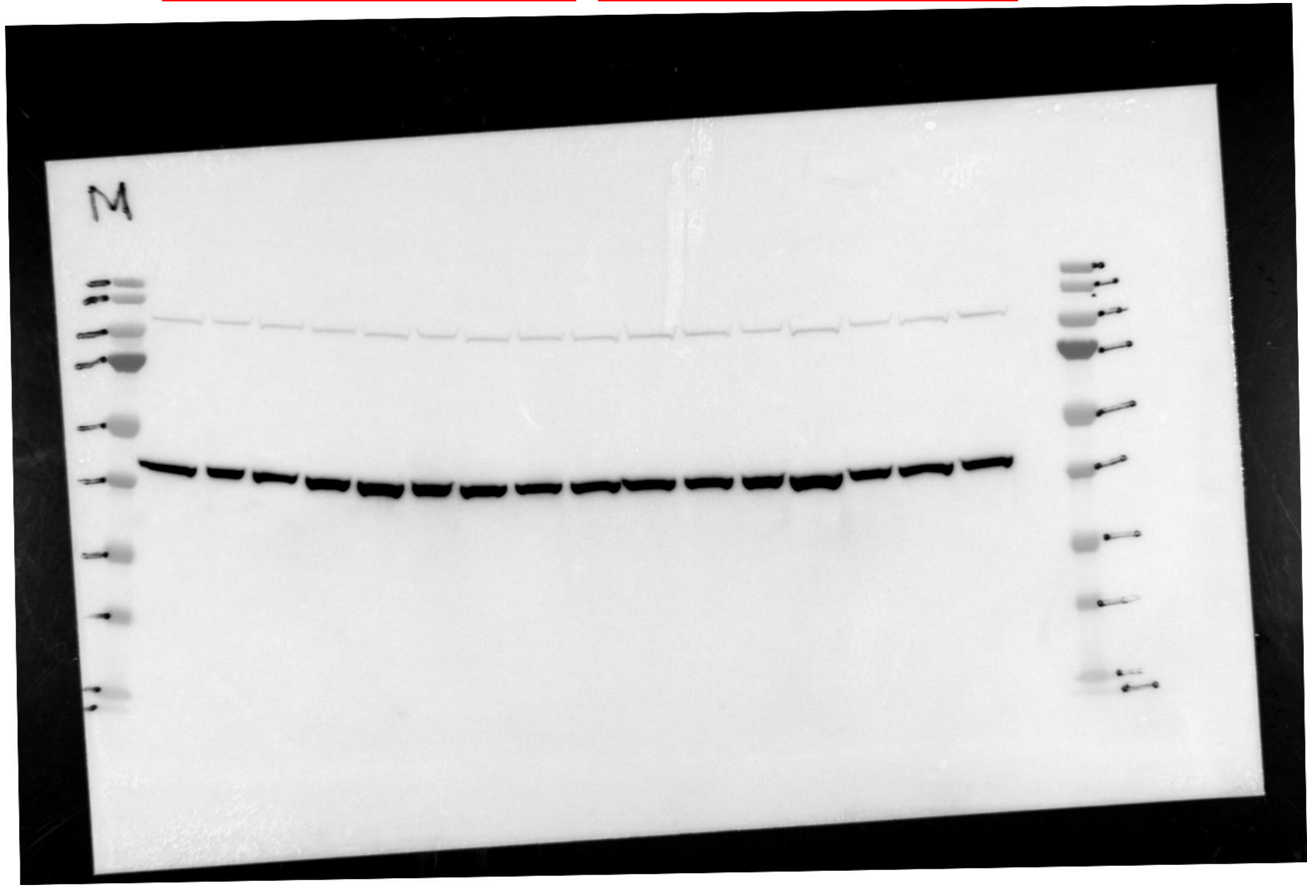

Females: TLR4 & β-actin

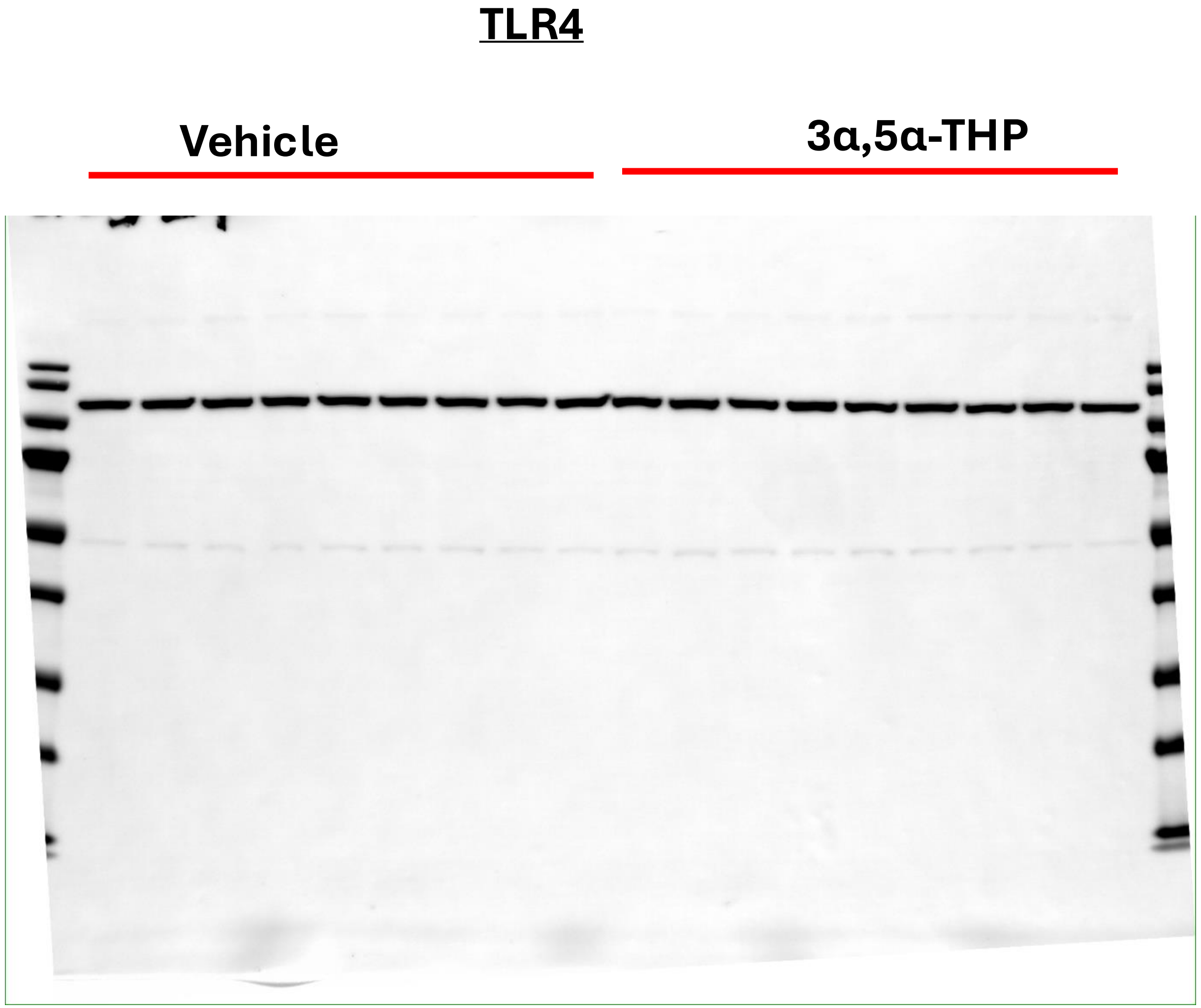

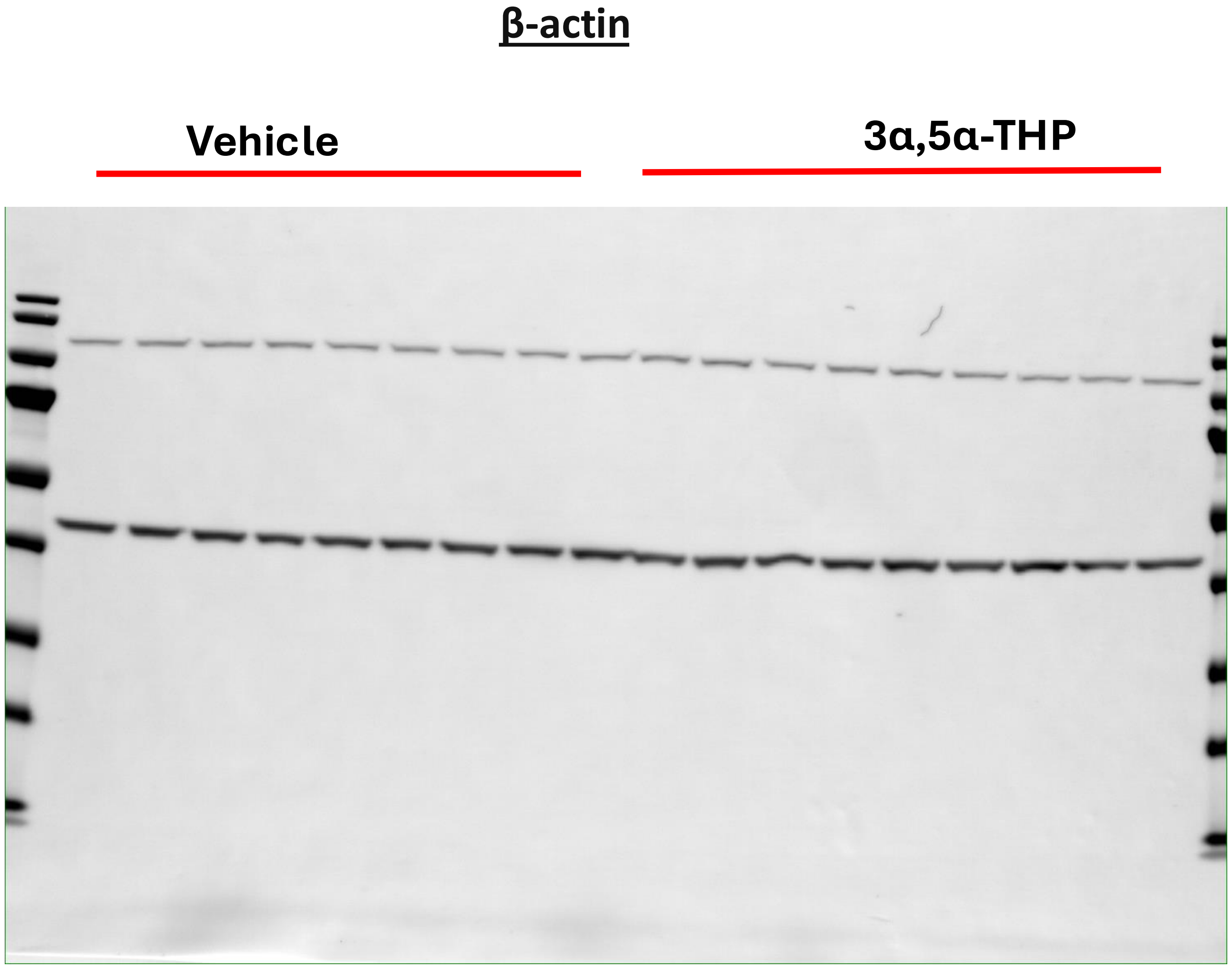

Males: IRAK4 & β-actin

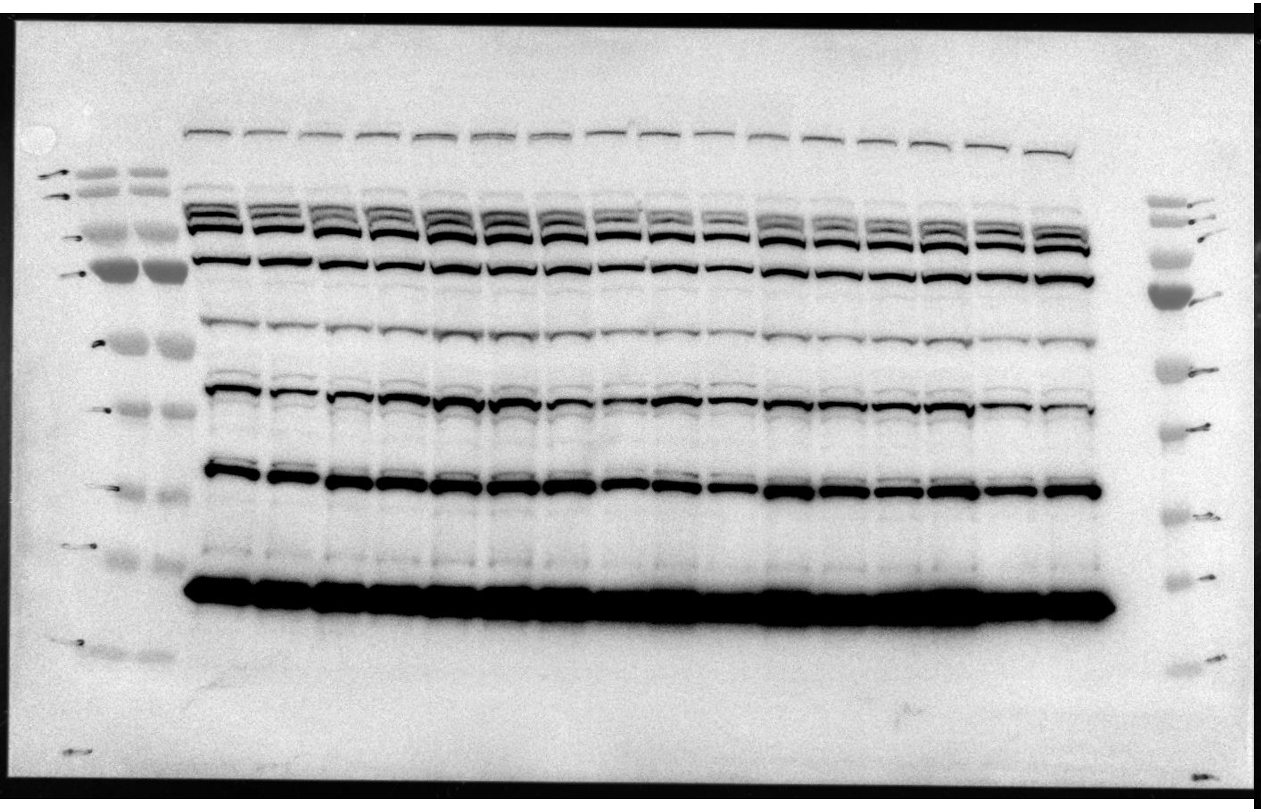

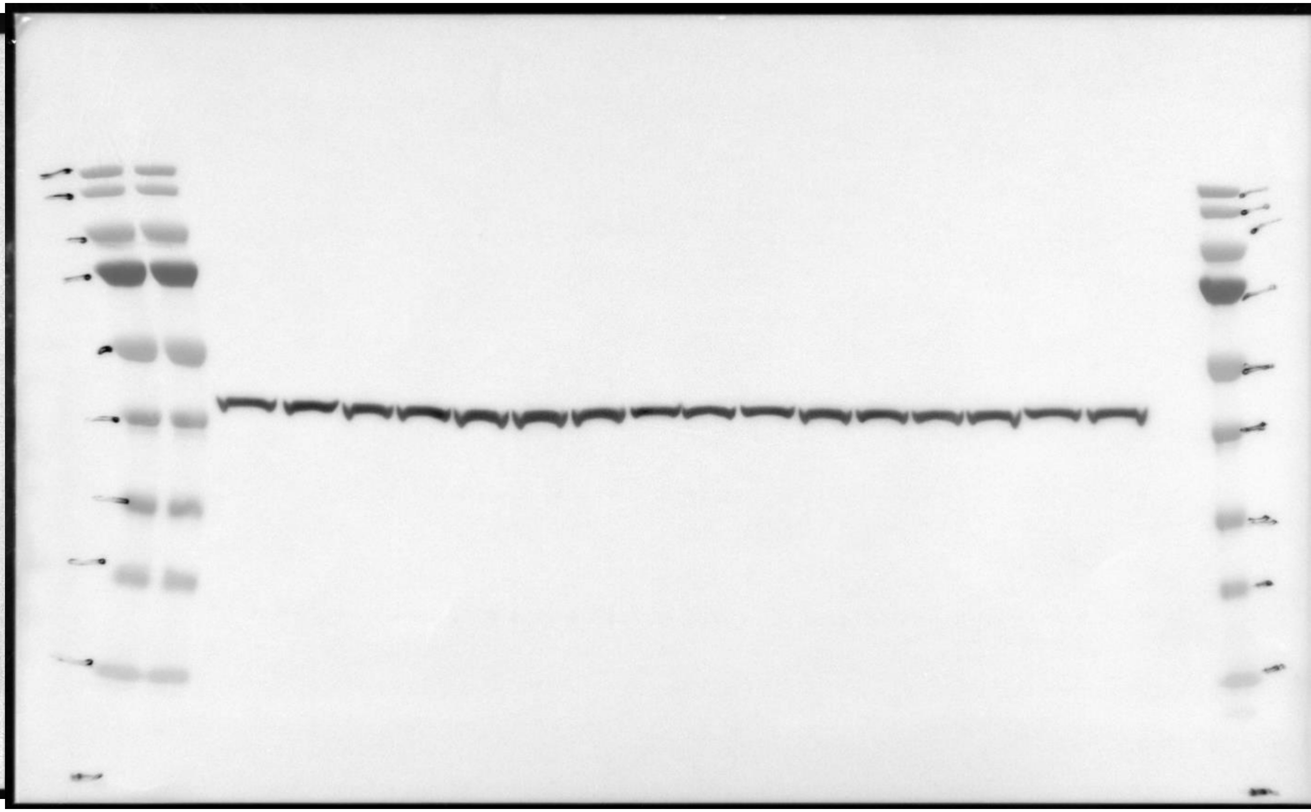

Females: IRAK4 & β-actin

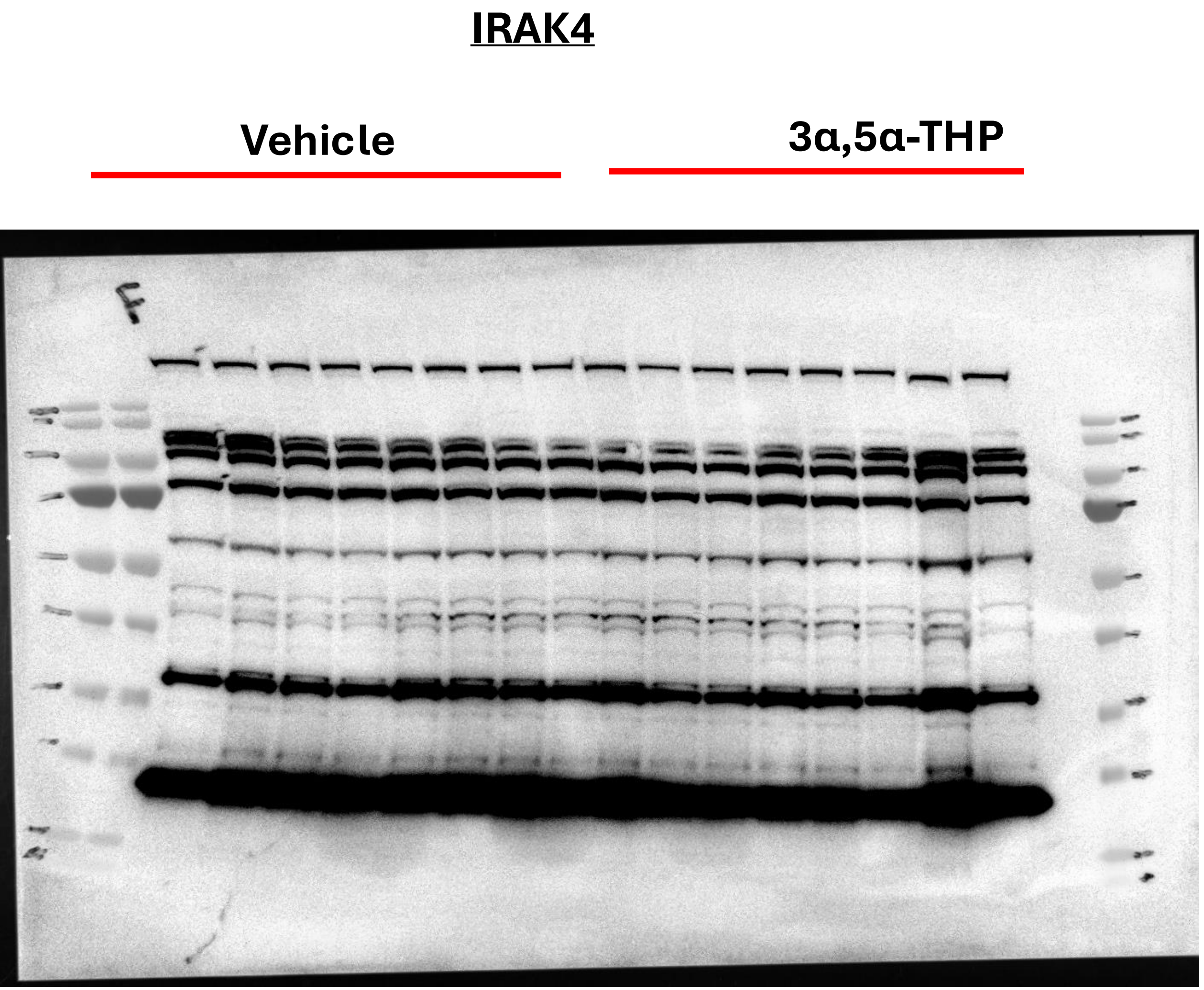

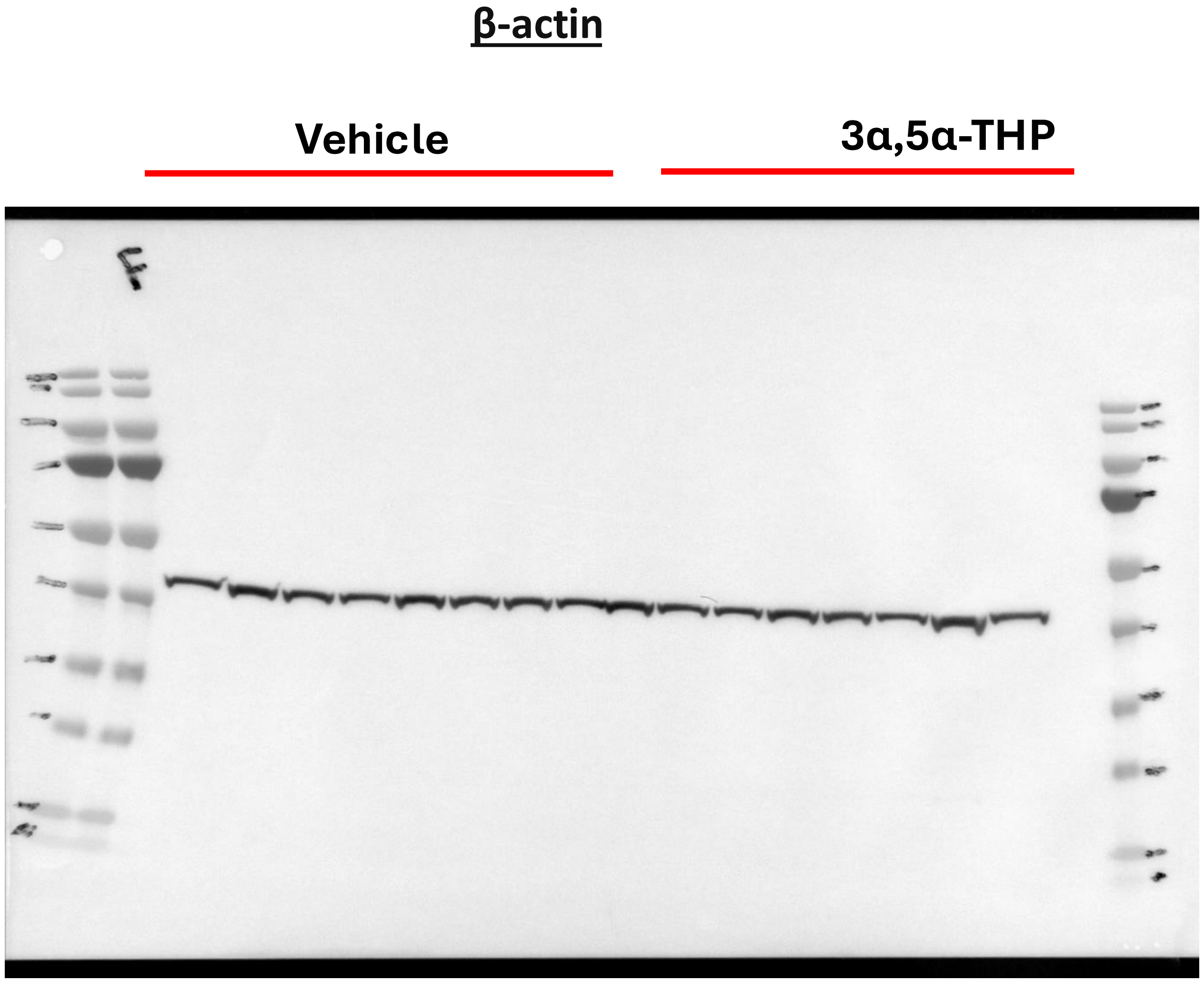

Males:CLIP170 & β-actin

CLIP170

Vehicle 3α,5α-THP

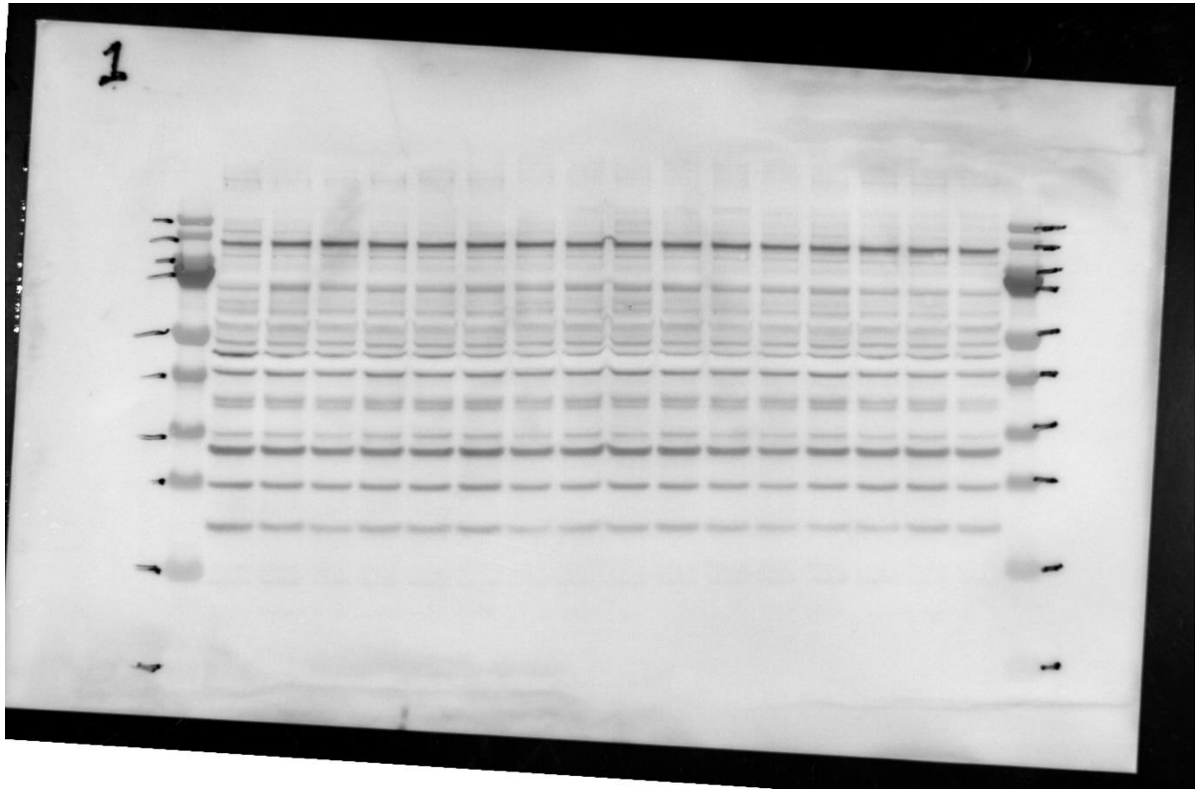

β-actin

Vehicle 3α,5α-THP

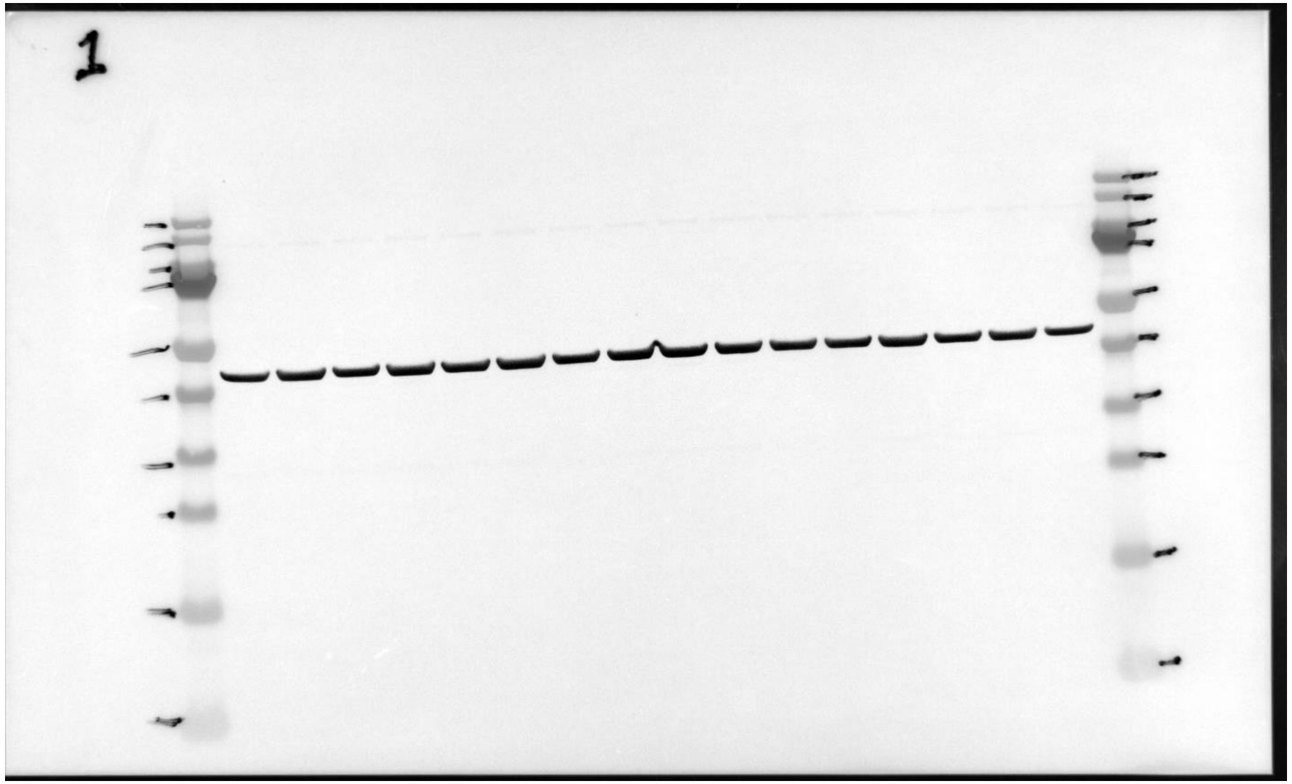

Females:CLIP170 & β-actin

CLIP170

Vehicle **3α,5α-THP**

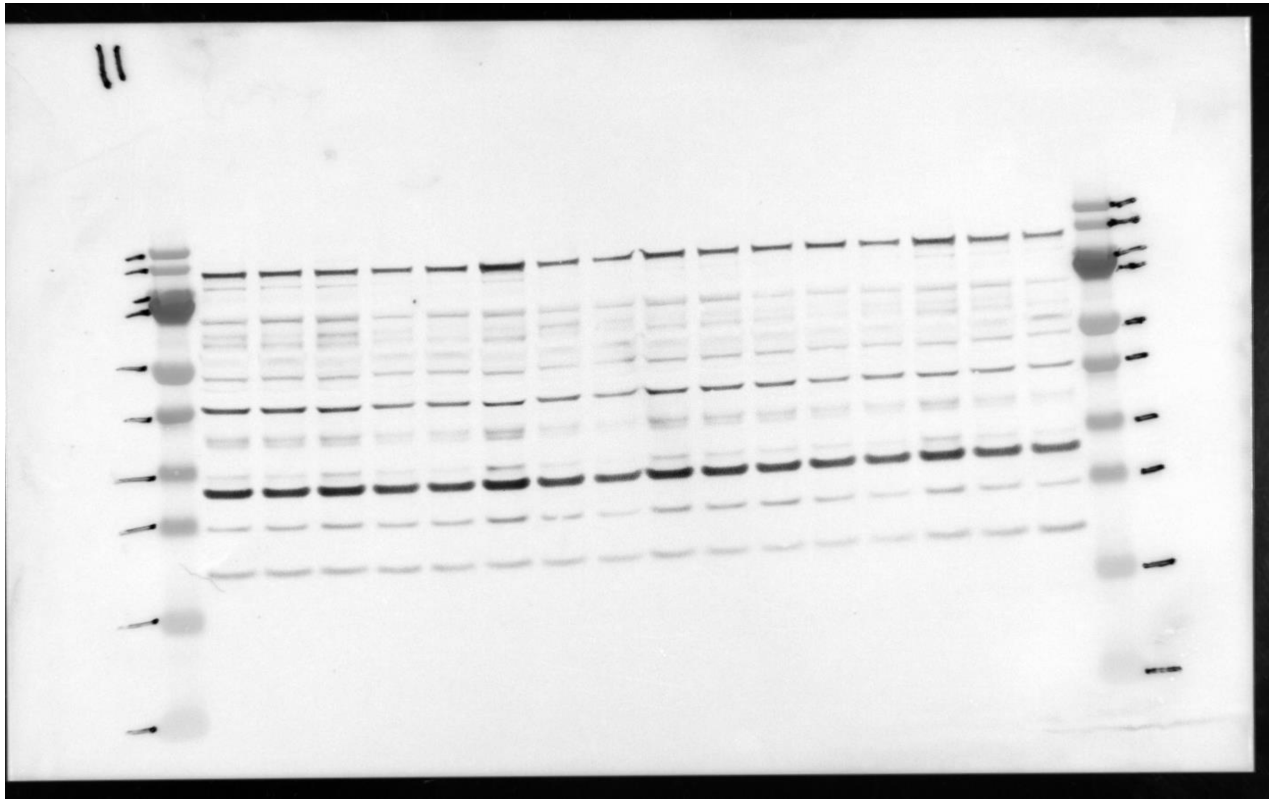

β-actin

Vehicle **3α,5α-THP**

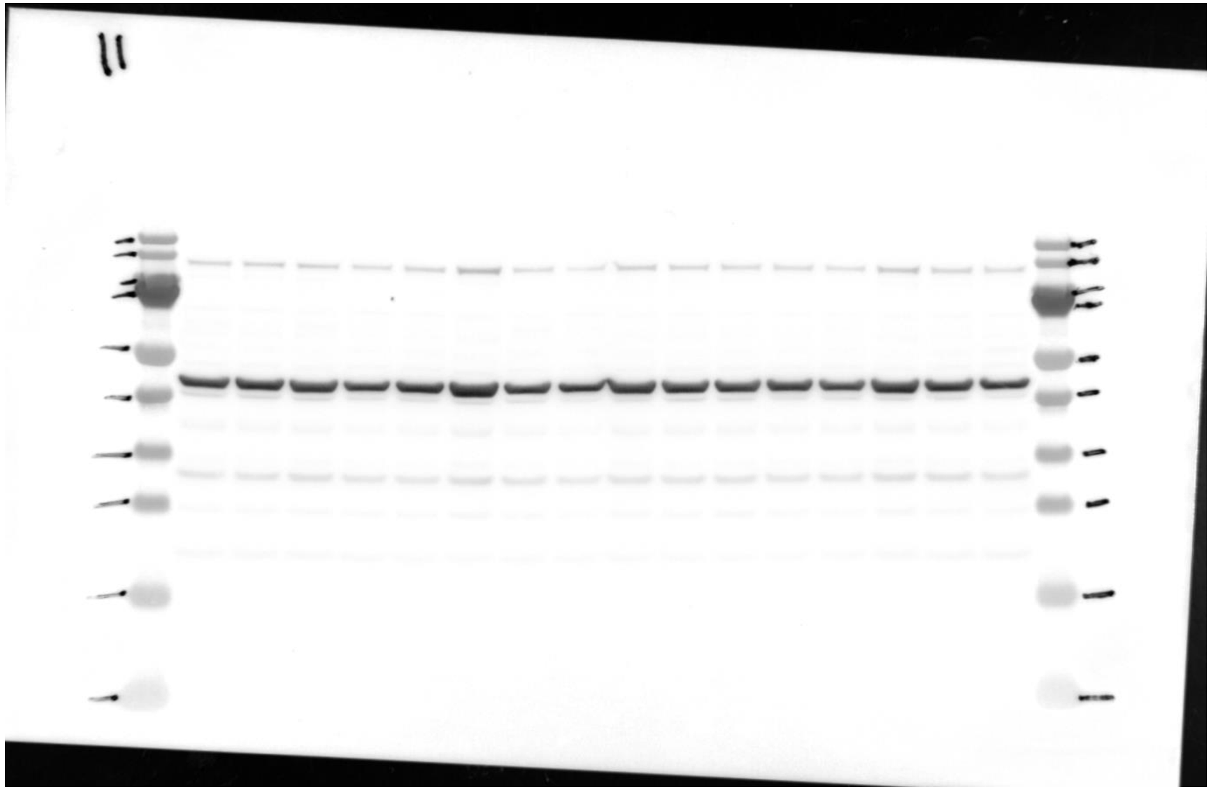

Males: BTK & β-actin

BTK

Vehicle **3α,5α-THP**

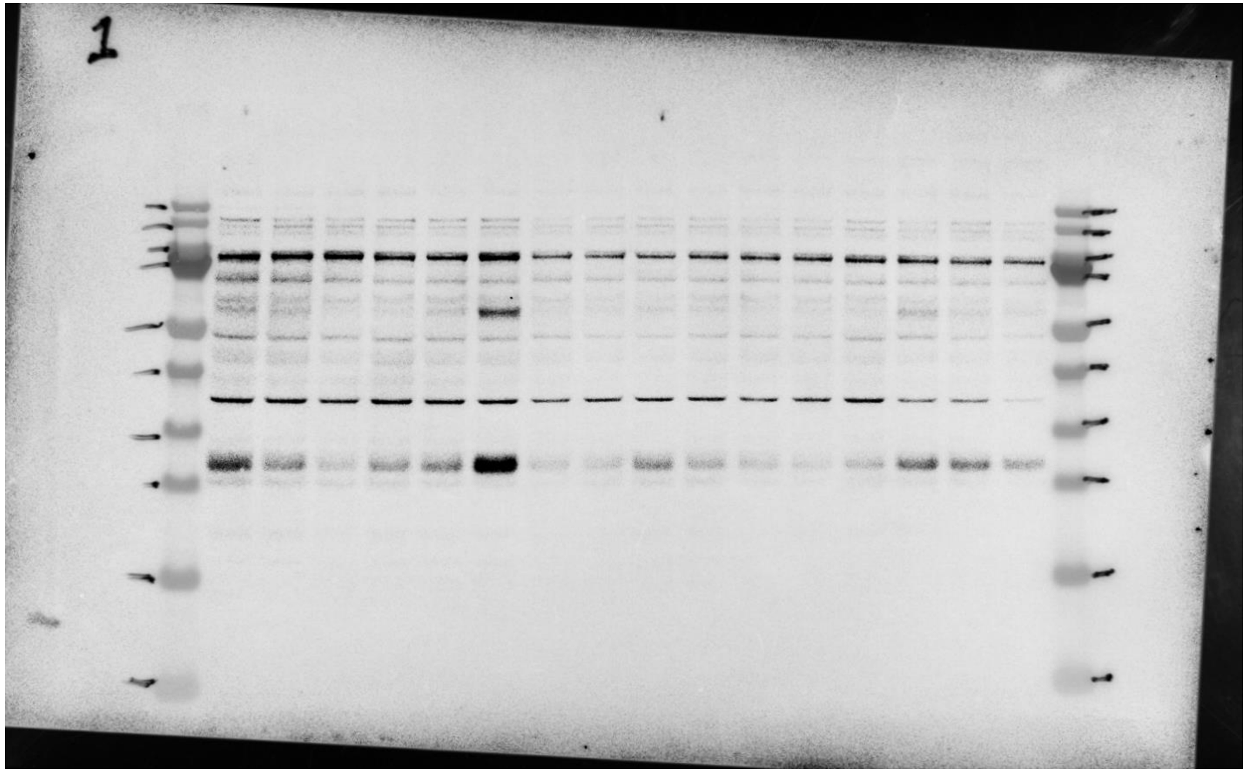

β-actin

Vehicle **3α,5α-THP**

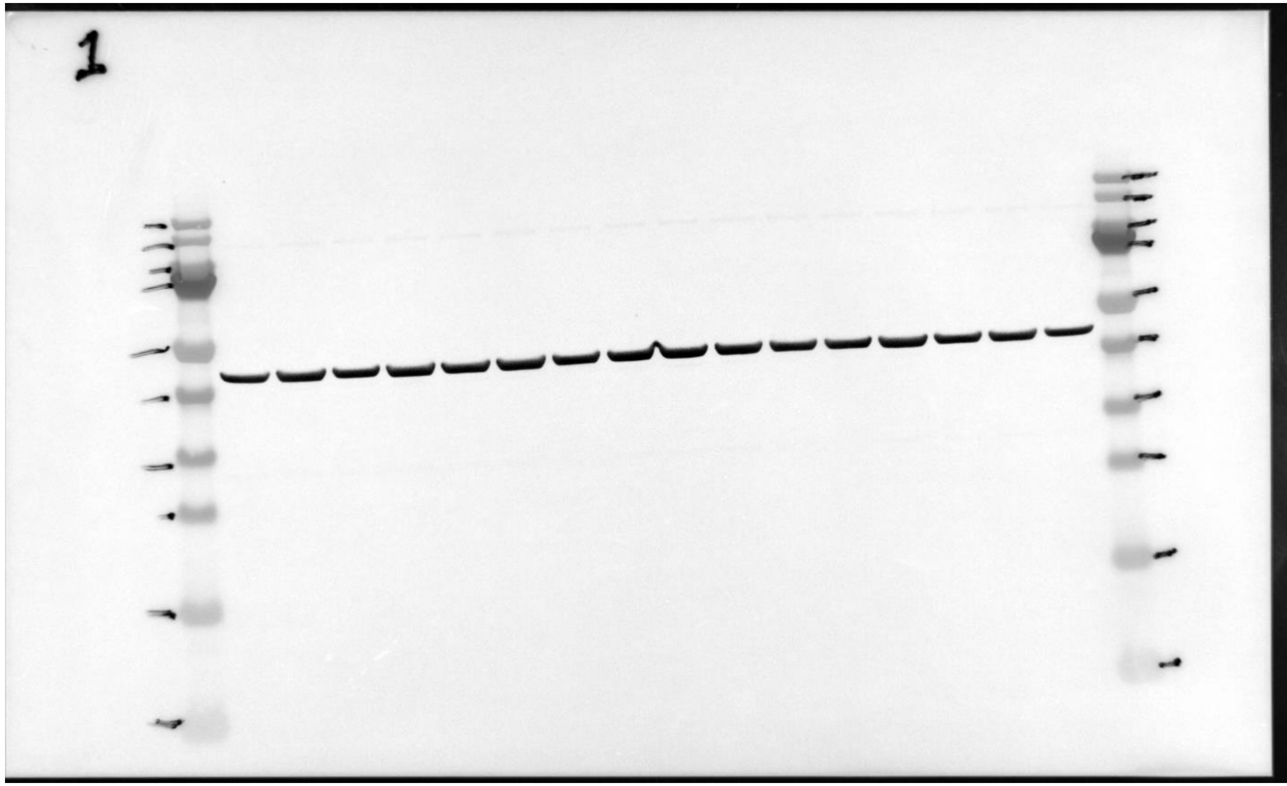

Females: BTK & β-actin

BTK

Vehicle **3α,5α-THP**

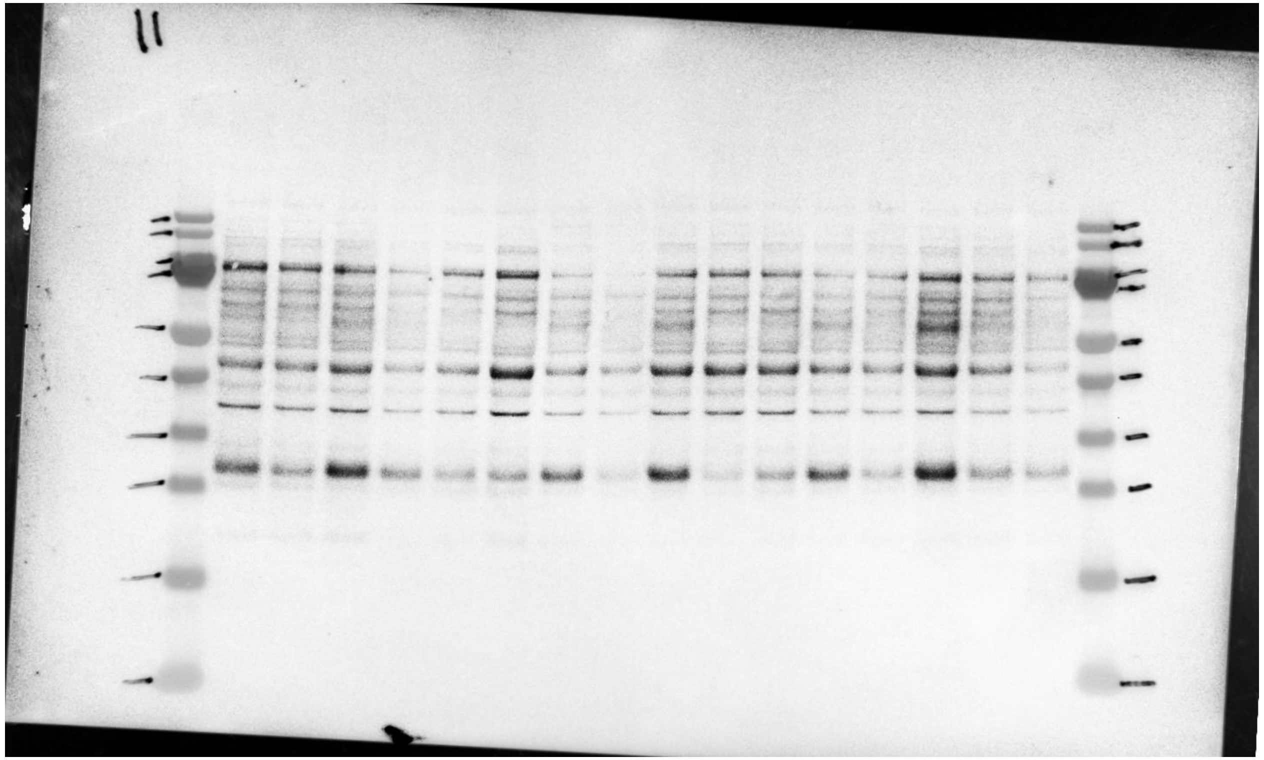

β-actin

Vehicle **3α,5α-THP**

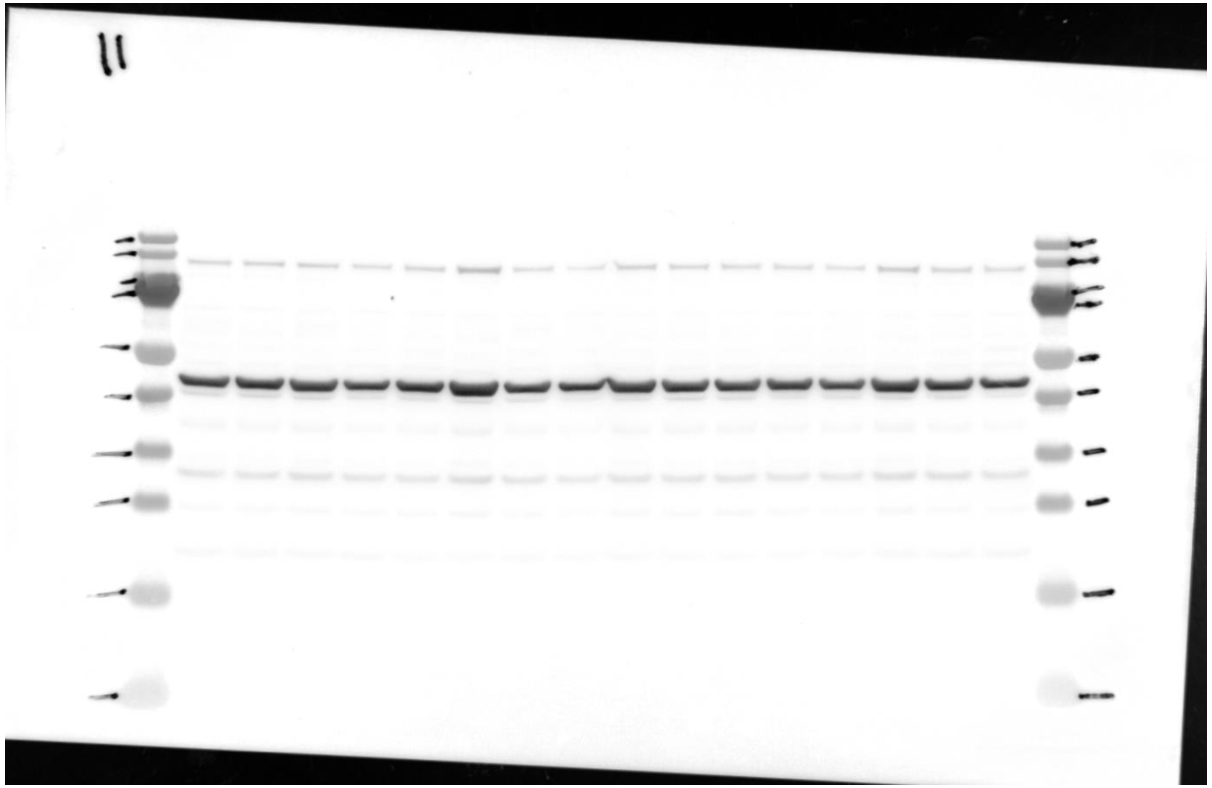

Males: IRAK1 & β-actin

IRAK1

Vehicle 3α,5α-THP

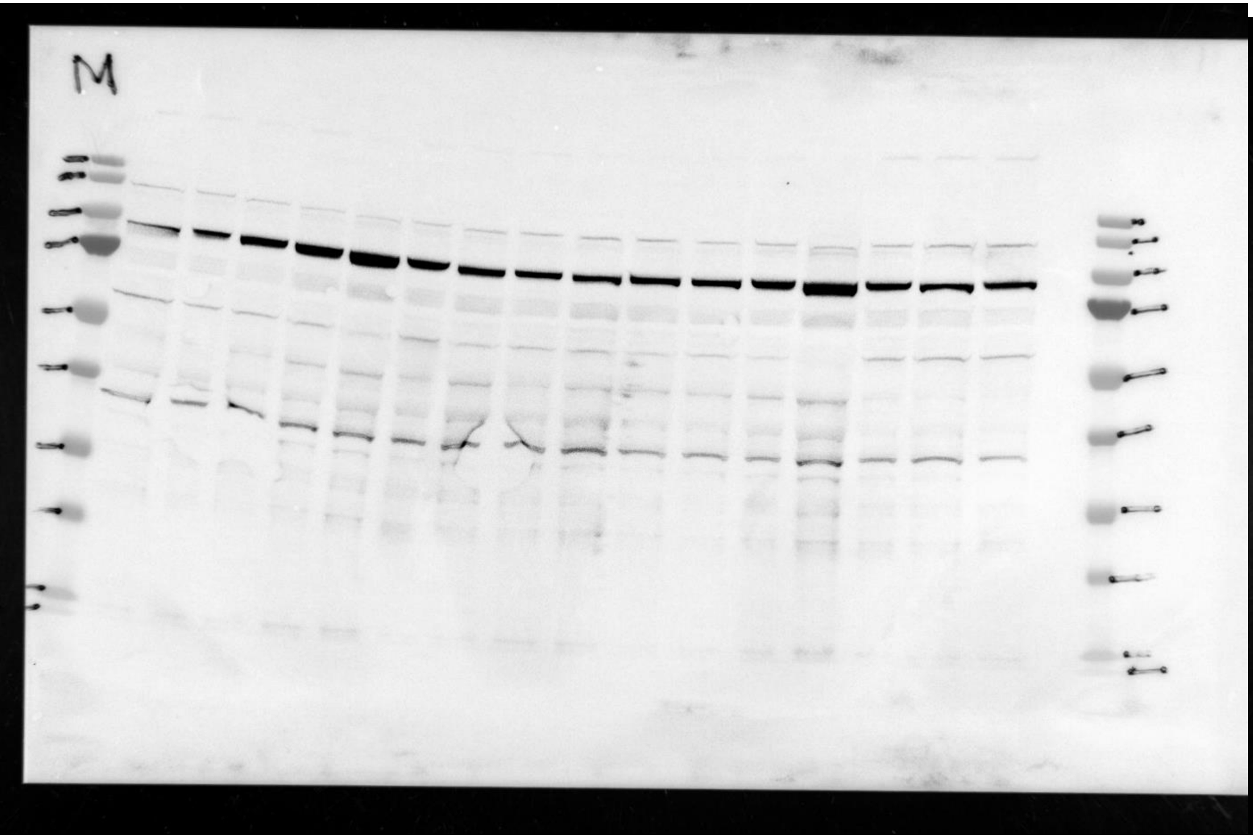

β-actin

Vehicle 3α,5α-THP

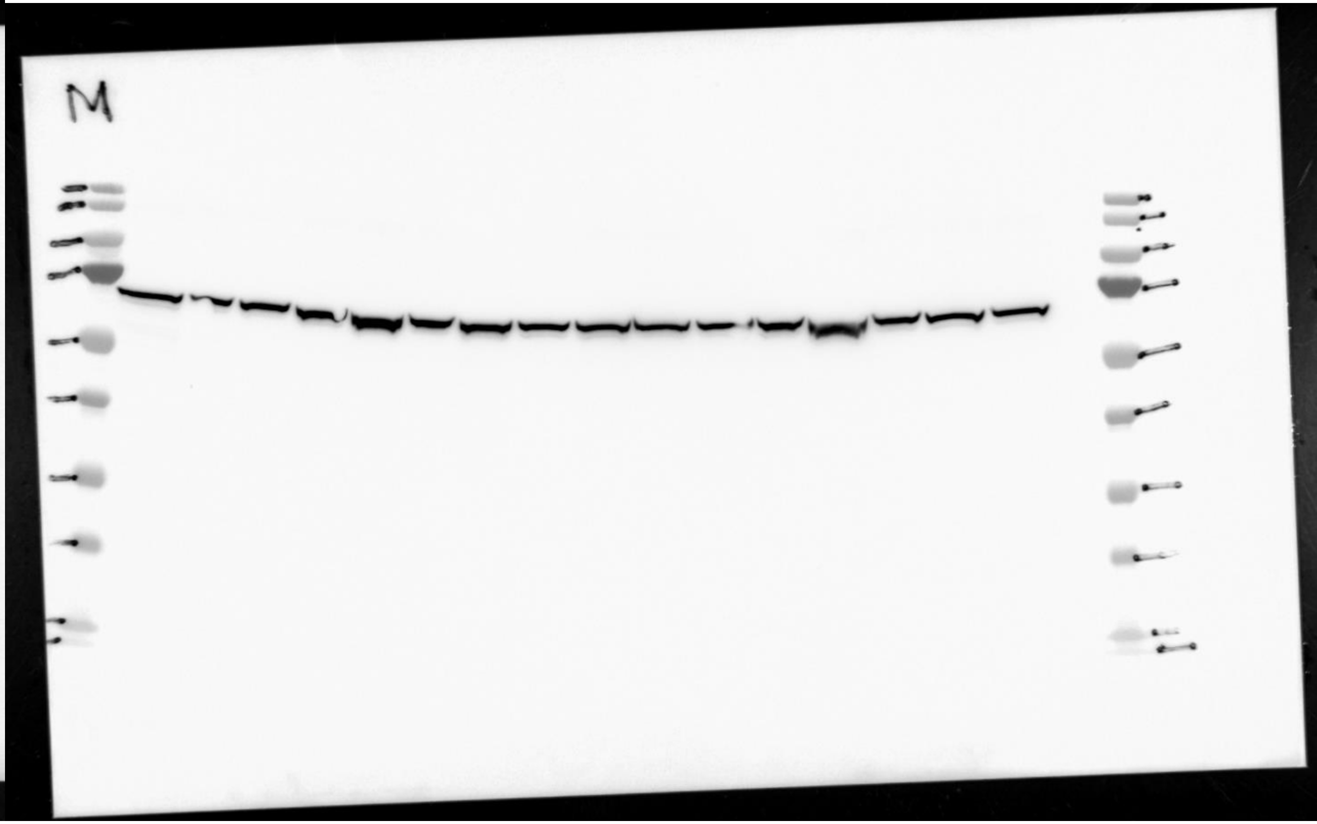

Females: IRAK1 & β-actin

IRAK1

Vehicle **3α,5α-THP**

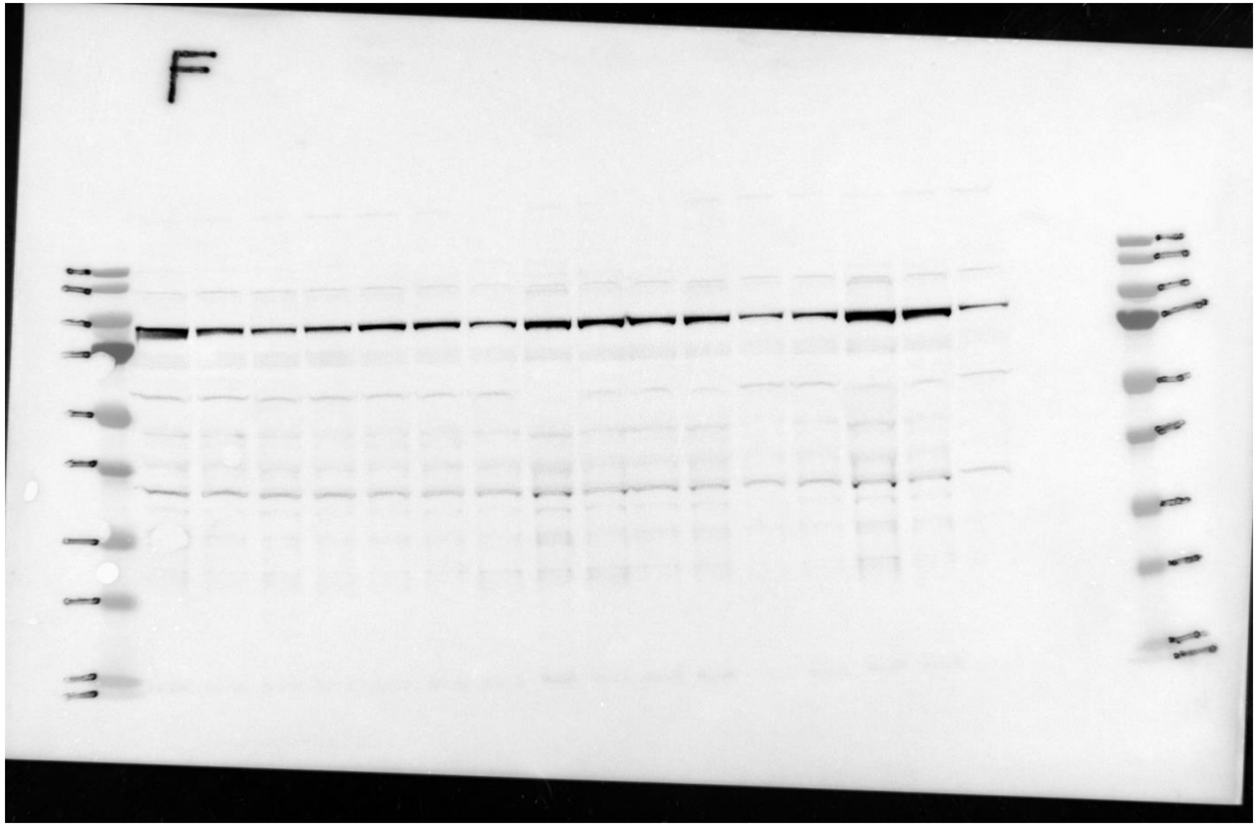

β-actin

Vehicle **3α,5α-THP**

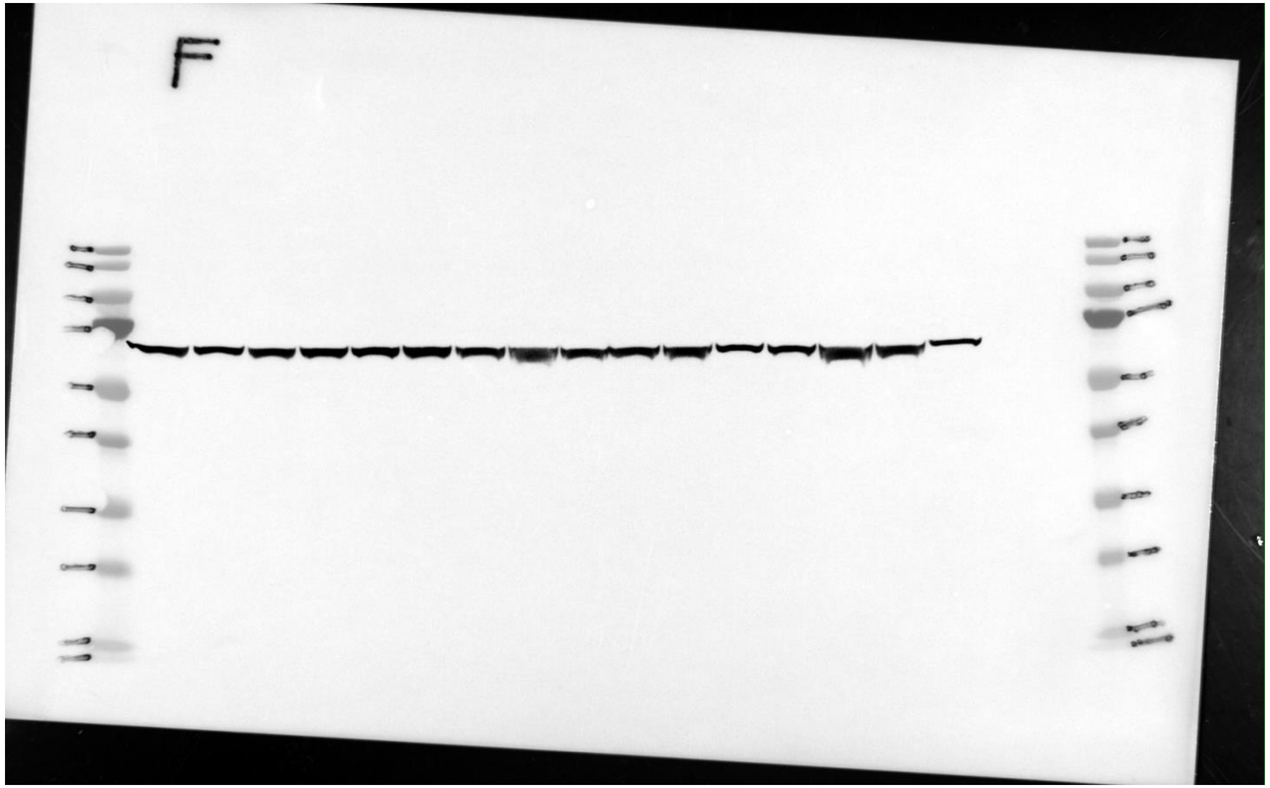

Figure 3 Western Blots

Figure 3A Males: MD-2 & β-actin

MD-2

Vehicle 3α,5α-THP

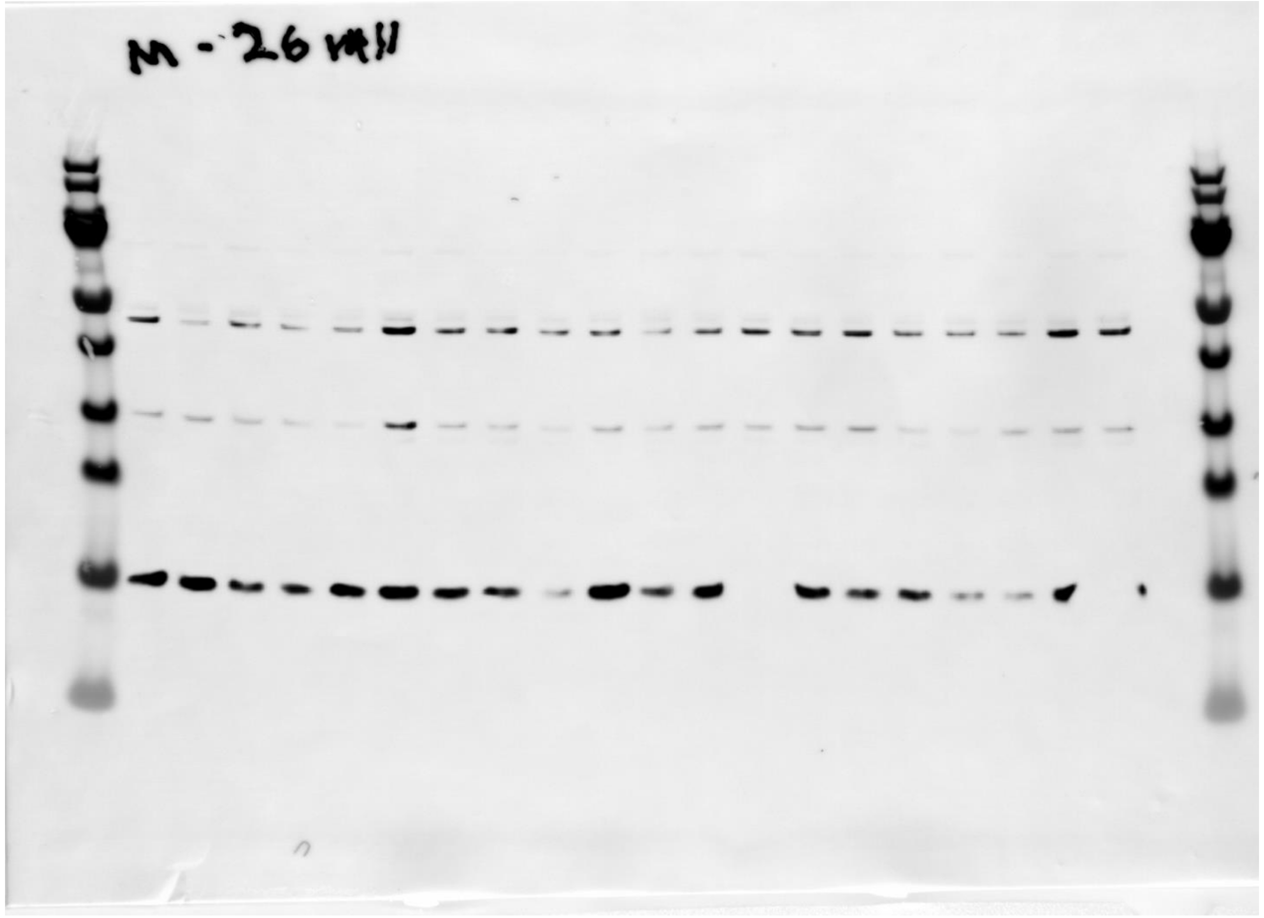

β-actin

Vehicle 3α,5α-THP

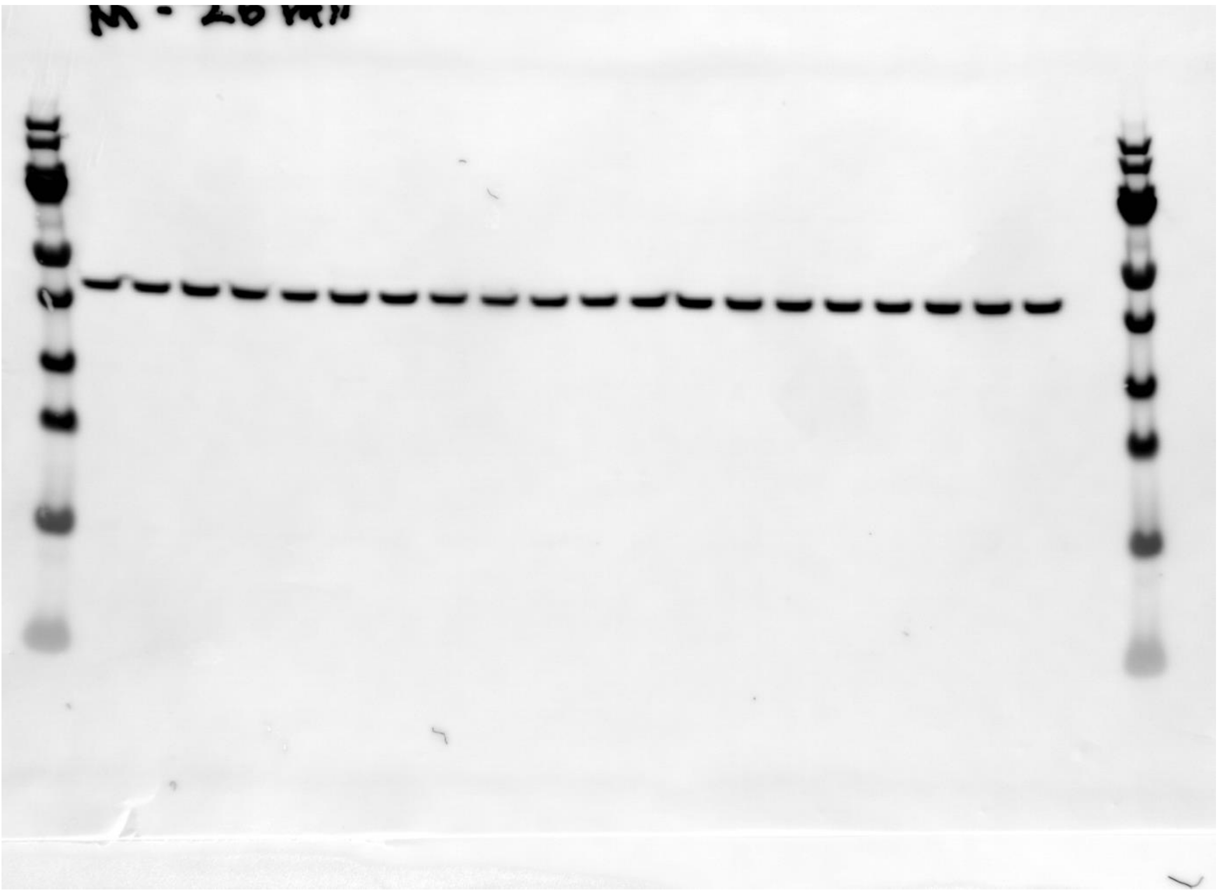

Figure 3A Females: MD-2 & β-actin

MD-2

Vehicle

3α,5α-THP

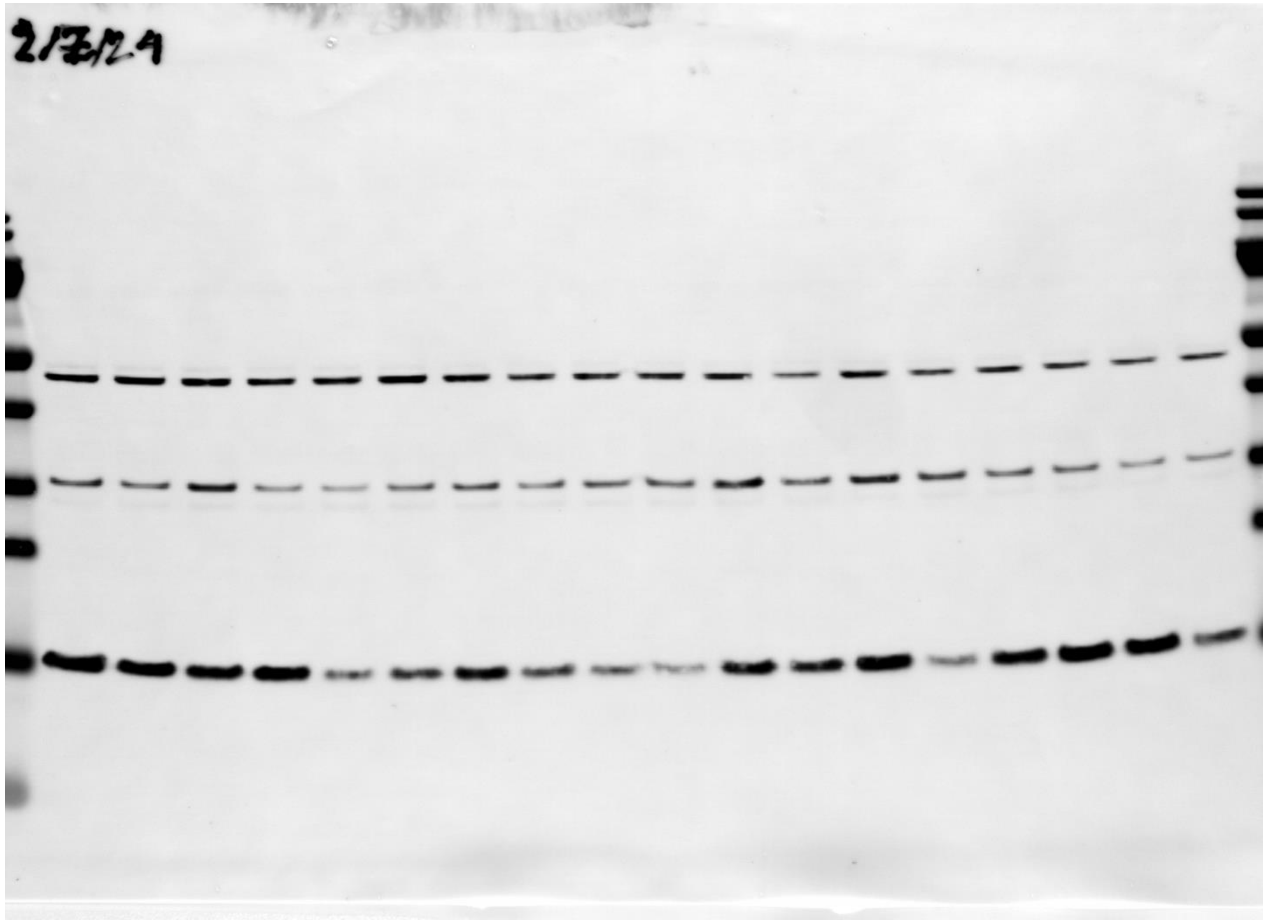

β-actin

Vehicle

3α,5α-THP

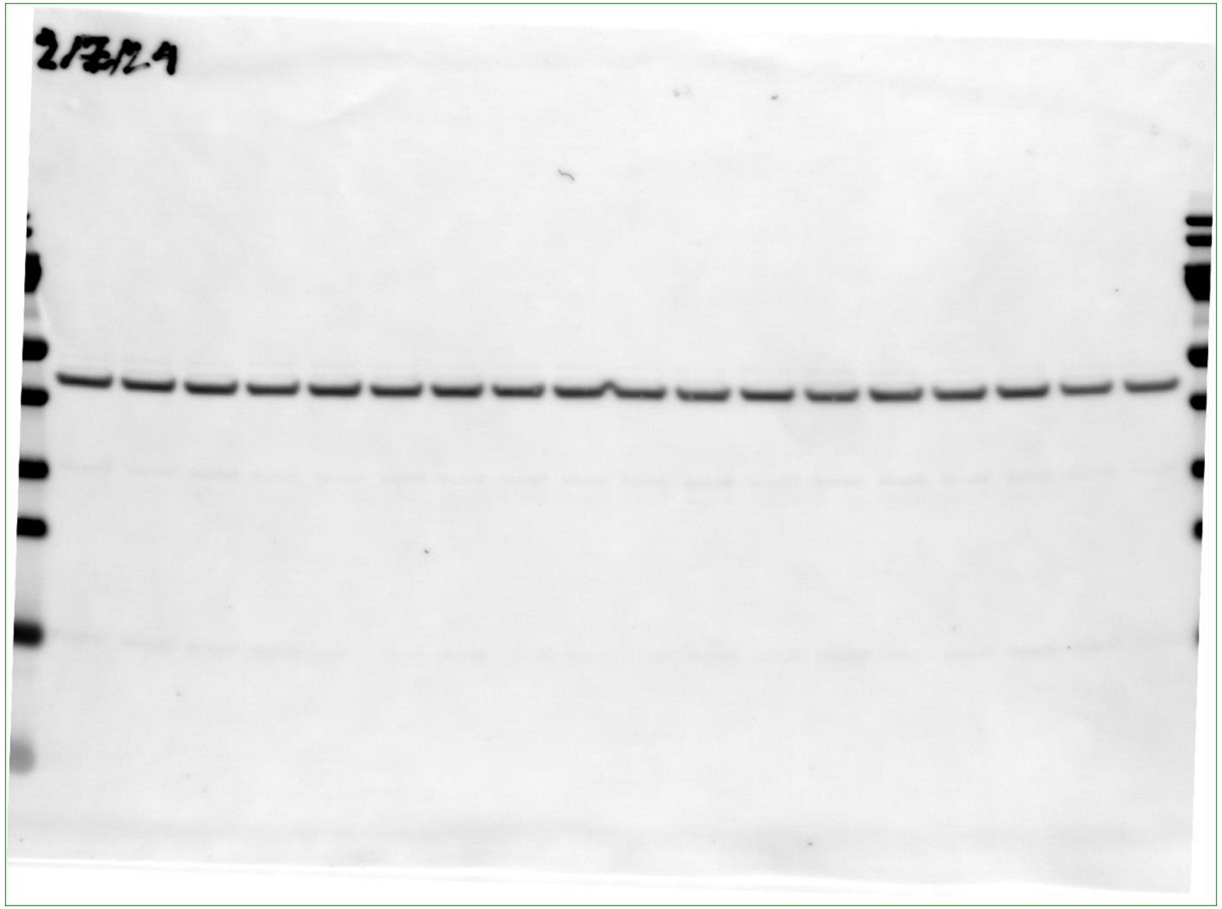

Figure 2C Males: IP TLR4:MD-2

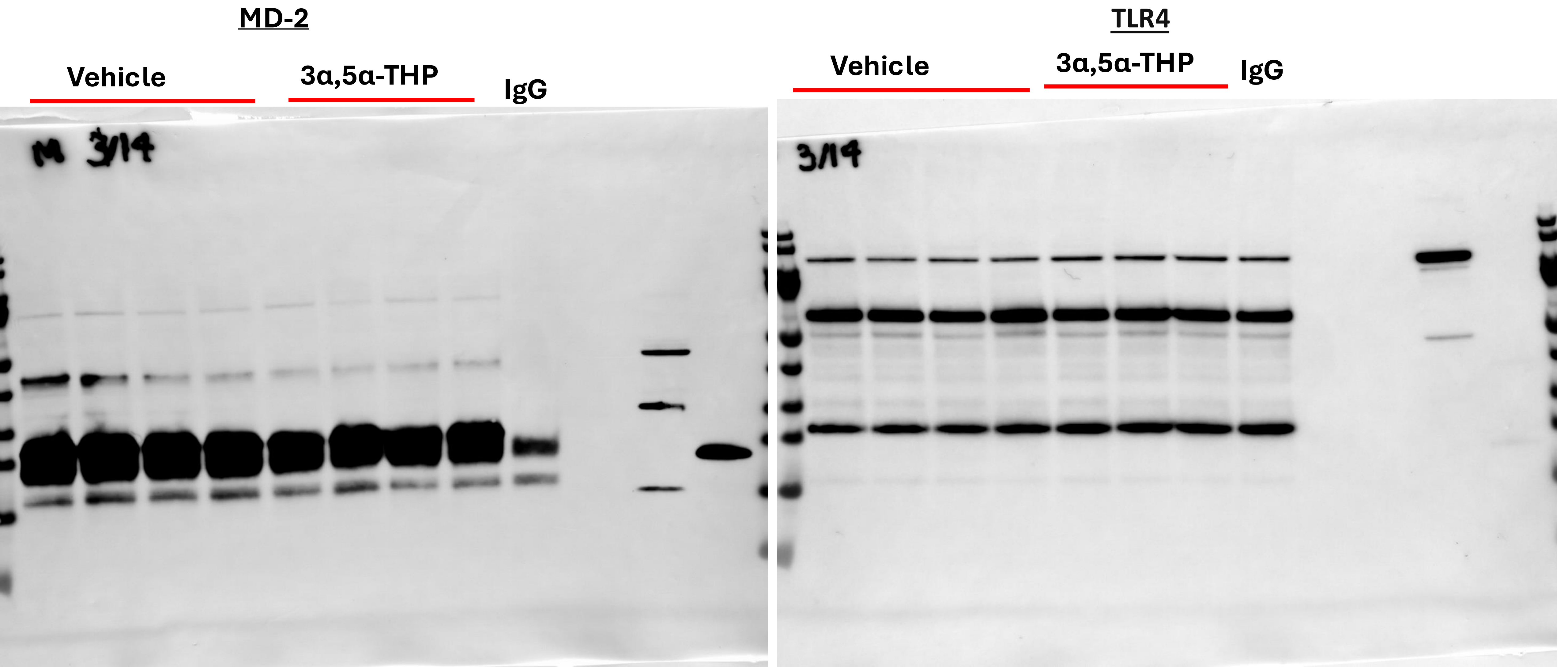

Figure 2E Females: IP TLR4:MD-2

MD-2

Vehicle | **3α,5α-THP** | **IgG**

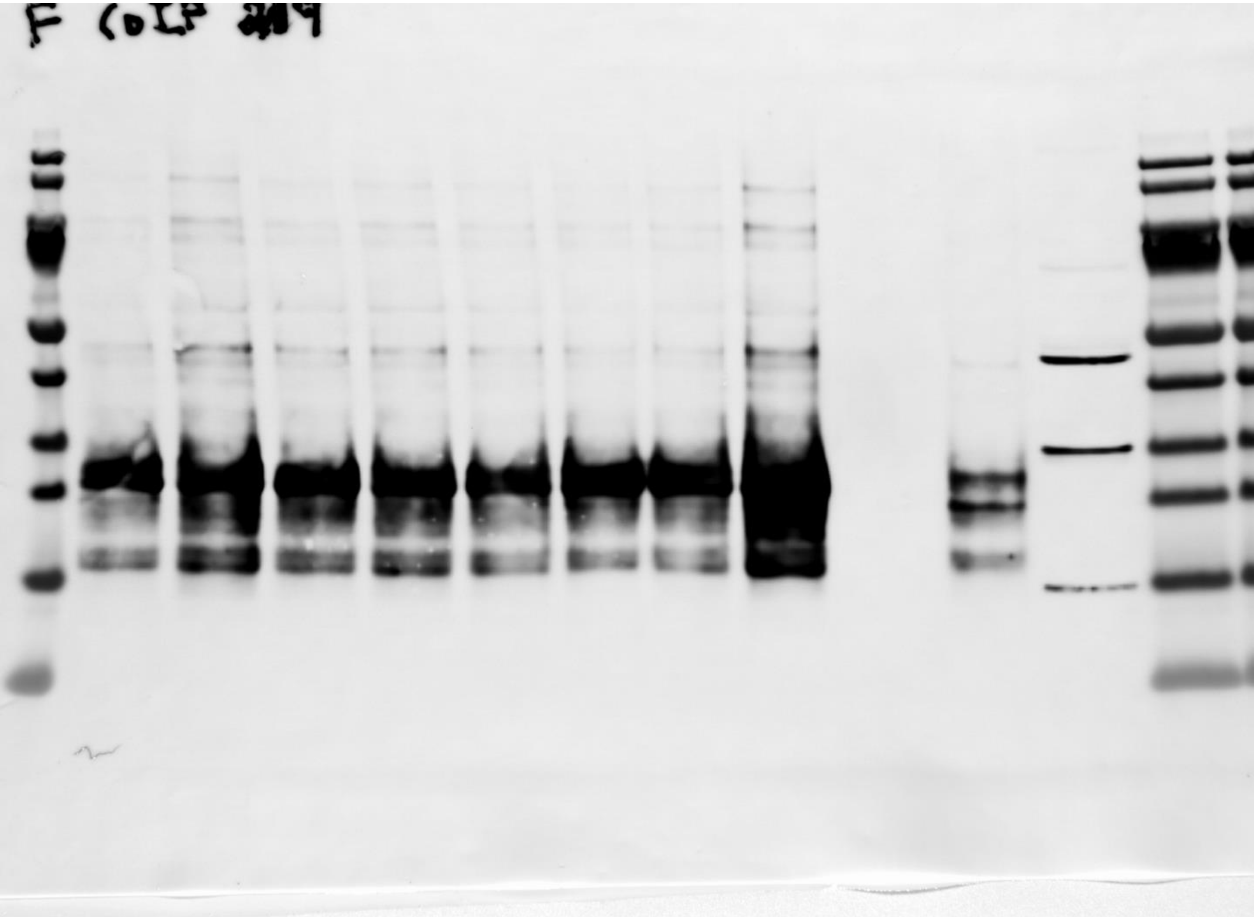

TLR4

Vehicle | **3α,5α-THP** | **IgG**

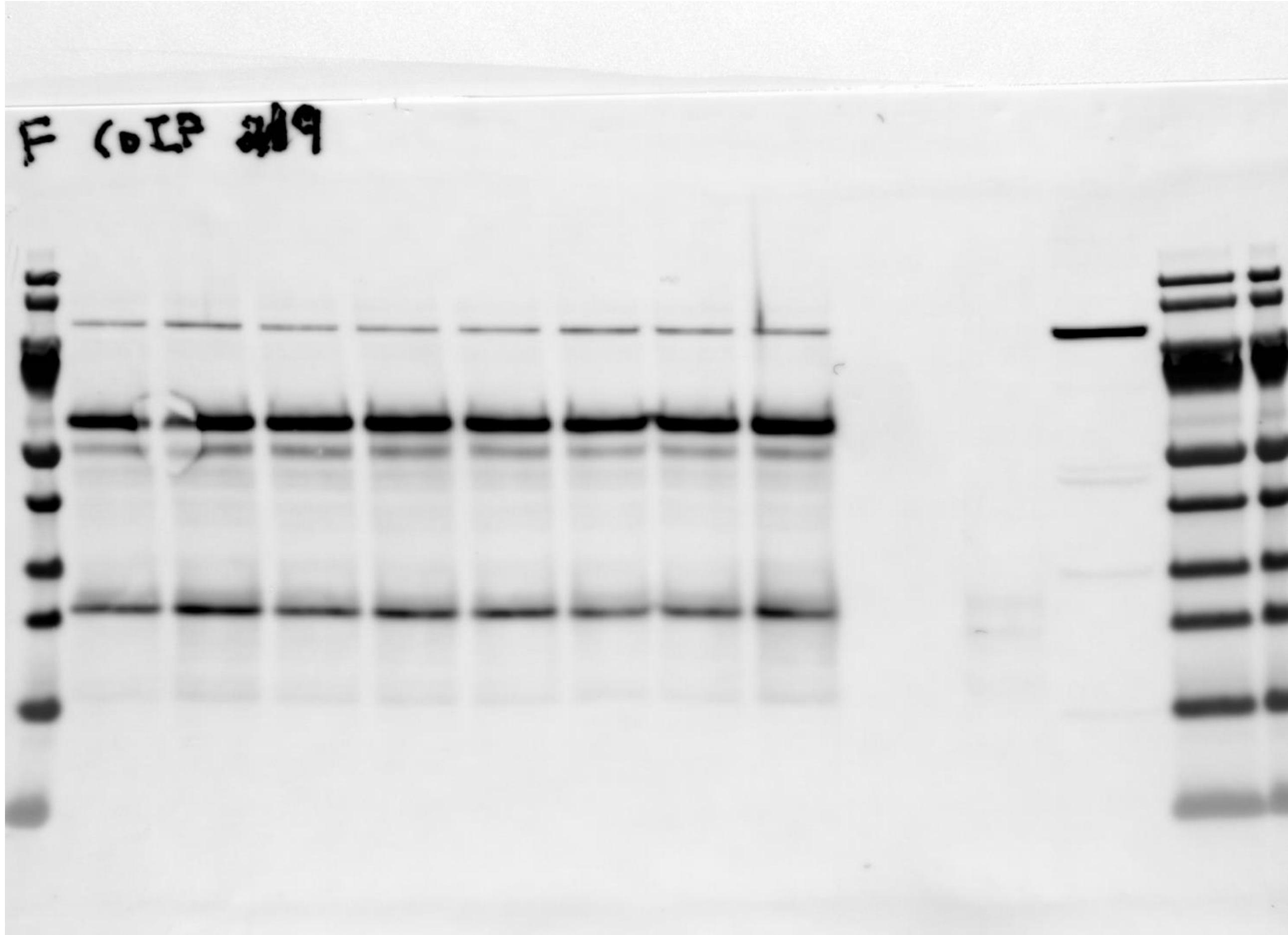

Supplement: Supplementary file 1 [file biomolecules-14-01441-s001.zip › Biomolecules_Data_Images.pdf]
