# Supplementary material for: Novel Inhibitory Actions of Neuroactive Steroid [3α,5α]-3-Hydroxypregnan-20-One on Toll-like Receptor 4-Dependent Neuroimmune Signaling
Source: Biomolecules. 2024 Nov 13;14(11):1441. doi: 10.3390/biom14111441 (PMC11591752; doi:10.3390/biom14111441)

Movie M1: MD trajectory of the apo MD-2 where the protein is shown in cartoon representation.

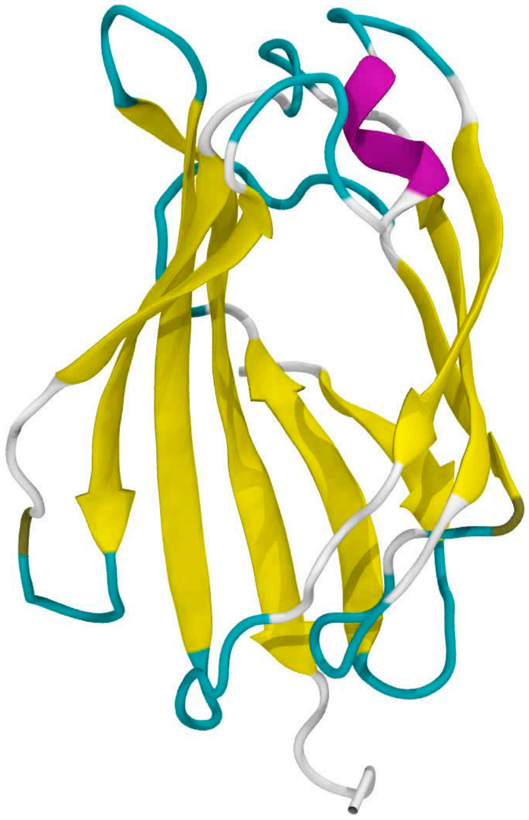

Movie M2: MD trajectory of MD-2 in complex with the neuroactive steroid (3α,5α)-3-hydroxypregnan-20-one (3α,5α-THP). The protein is represented as a cartoon and 3α,5α-THP is depicted in van der Waals representation.

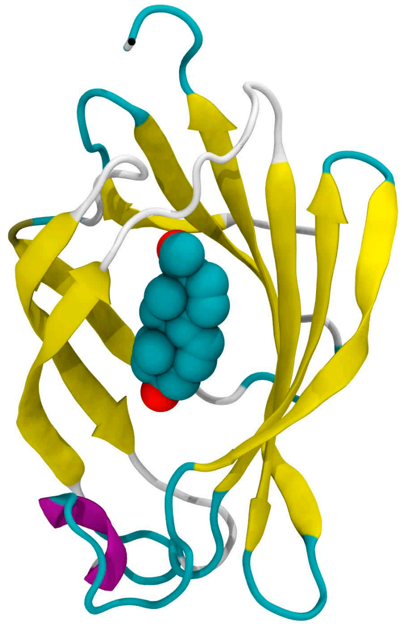

Supplement: Supplementary file 1 [file biomolecules-14-01441-s001.zip › SFigure_Movies.pdf]
